# KAT6B overexpression rescues embryonic lethality in homozygous null KAT6A mice restoring vitality and normal lifespan

**DOI:** 10.1038/s41467-025-57155-4

**Published:** 2025-02-25

**Authors:** Maria. I. Bergamasco, Yuqing Yang, Alexandra L. Garnham, Bilal N. Sheikh, Gordon K. Smyth, Anne. K. Voss, Tim Thomas

**Affiliations:** 1 The Walter and Eliza Hall Institute of Medical Research, Parkville, VIC Australia; 2 Department of Medical Biology, The University of Melbourne, Parkville, VIC Australia; 3 Helmholtz Institute for Metabolic, Obesity and Vascular Research (HI-MAG) of the Helmholtz Center Munich, Leipzig, Germany; 4 Medical Faculty, University of Leipzig, Leipzig, Germany; 5 School of Mathematics and Statistics, University of Melbourne, Parkville, VIC Australia

**Keywords:** Epigenetics, Development, Embryology

## Abstract

Closely related genes typically display common essential functions but also functional diversification, ensuring retention of both genes throughout evolution. The histone lysine acetyltransferases KAT6A (MOZ) and KAT6B (QKF/MORF), sharing identical protein domain structure, are mutually exclusive catalytic subunits of a multiprotein complex. Mutations in either *KAT6A* or *KAT6B* result in congenital intellectual disability disorders in human patients. In mice, loss of function of either gene results in distinct, severe phenotypic consequences. Here we show that, surprisingly, 4-fold overexpression of *Kat6b* rescues all previously described developmental defects in *Kat6a* mutant mice, including rescuing the absence of hematopoietic stem cells. *Kat6b* restores acetylation at histone H3 lysines 9 and 23 and reverses critical gene expression anomalies in *Kat6a* mutant mice. Our data suggest that the target gene specificity of KAT6A can be substituted by the related paralogue KAT6B, despite differences in amino acid sequence, if KAT6B is expressed at sufficiently high levels.

## Abstract (web summary)

KAT6A and KAT6A are epigenetic regulators with critical functions in embryonic development. Mutations in either KAT6A or KAT6B result in distinct congenital disorders in human patients. Surprisingly, overexpression of KAT6B can rescue loss of KAT6A.

## Introduction

In eukaryotic cells, transcription is influenced by the nucleosomal barriers imposed by histone proteins. Post-translational modifications of chromatin, for example histone acetylation, regulate chromatin structure and influence the nucleosome landscape such as to promote or suppress the expression of target loci^1^. Lysine acetylation is catalysed by acetyltransferase enzymes and generally associated with increased gene expression and the regulation of fundamental cellular functions^2,3^ Three families of histone acetyltransferases, with well-described acetylation domains^4,5^, are currently defined based on structural and sequence conservation: the MYST family^6^, the GNAT family^7^ and the p300/CBP family^8^.

Interestingly, histone lysine acetyltransferases typically occur as pairs of highly similar proteins. MYST family members KAT6A (MOZ) and KAT6B (QKF/MORF) share identical domain structure and high sequence similarity across all functional domains^6^. Likewise, both the GNAT family proteins, KAT2A (GCN5) and KAT2B (PCAF)^9^ and the p300/CBP family proteins KAT3A (CBP) and KAT3B (P300)^8,10,11^ share identical domain structure and high sequence similarity across all functional domains. Sequence and structural similarities between family members likely resulted from ancestral gene duplication events and subsequent neofunctionalisation^12^. Indeed, in vivo studies have demonstrated that these proteins have acquired independent functions^13,14^; however, the extent to which one protein within a pair of closely related chromatin regulatory proteins can replace the other, has not been assessed.

KAT6A and KAT6B are mutually exclusive catalytic subunits of a shared multi-protein complex including chromatin adaptor proteins of the BRPF family, primarily BRPF1, ING family, ING5 and ING4, and MEAF^15,16^. Both proteins have been shown to acetylate H3K9 and H3K23 in a range of cell and tissue systems^17–23^. The *KAT6A* and *KAT6B* genes are oncogenes. KAT6A (*MOZ*, monocytic leukaemia zinc finger gene) was originally identified in an aggressive form of acute myeloid leukaemia resulting from a translocation fusing it to KAT3A^24^. A number of other recurrent translocations of the *KAT6A* locus have since been identified in leukaemia with a variety of translocation partners^25^. Interestingly, loss of just one allele of *KAT6A* greatly enhances survival and disease latency in a mouse model of MYC driven lymphoma^26^. Similarly, *KAT6B* translocations have been identified in leukaemia and leiomyomata^27,28^. *KAT6A* and *KAT6B* mRNAs are commonly upregulated across a range of different cancer types^29^. This association with cancer has fuelled the development of drugs targeting both KAT6A and KAT6B proteins^30,31^, which have shown promise in clinical trials^32^.

In human patients, de novo mutations in *KAT6A* or *KAT6B* result in congenital disorders defined by cognitive impairment, developmental delay and craniofacial dysmorphogenesis^33–36^. Heterozygous mutations in the *KAT6A* gene result in Arboleda-Tham syndrome (ARTHS)^33,34^, while mutations in the *KAT6B* gene result in the Say-Barber-Biesecker-Young Simpson variant of Ohdo syndrome (SBBYSS)^35^ or Genitopatellar syndrome (GPS)^36^.

Deletion of the *Kat6a* or *Kat6b* gene in mice has identified their unique functions during development. Most *Kat6b*^*gt/gt*^ mice, deficient in 90% of *Kat6b* mRNA, die at birth on a 129 Sv inbred genetic background, while the 20% surviving *Kat6b*^*gt/gt*^ mice show a failure to thrive, short stature and a squared skull^37,38^. *Kat6b*^*gt/gt*^ mice show abnormalities of the brain, including a reduced number of GAD67-positive interneurons and reduced numbers of layer V pyramidal neurons^37^, as well as fewer neural stem cells (NSCs) in the adult subventricular zone. *Kat6b*^*gt/gt*^ NSCs show reduced proliferation and self-renewal and a reduced capacity to differentiate into neurons^39^. Mice homozgous for a null allele of *Kat6b* show premature ossification and increased bone density in the skull and long bones^40^. *Kat6b*^*+/−*^ show learning and memory defects that can be ameliorated by increasing histone acetylation^41^. These findings suggest that KAT6B function is primarily required for normal brain, craniofacial and skeletal development. Consistently, *Kat6b* is most strongly expressed in the primordia of these tissues during embryonic development^37^.

Loss of KAT6A, either through deletion of the carboxy terminus or deletion of exons 5 to 9^17,18^, results in lethality at embryonic day (E) 14-18, depending on the genetic background^18^, an absence of transplantable haematopoietic stem cells (HSCs)^42,43^, an extensive anterior homeotic transformation^17^, cleft palate^18,44^ and cardiac and large vessel defects^18,45^. The roles of KAT6A within the foetal haematopoietic system, have been shown to depend upon its histone acetyltransferase function^46,47^. These tissues are not affected in mice lacking KAT6B, with the exception of a reduction, but not absence of hematopoietic stem cells in *Kat6b*^*−/−*^ embryos^23^.

The recent development of drugs targeting both KAT6A and KAT6B has highlighted the need to better understand the cellular functions of these chromatin regulators. While it is clear from single knockout studies that one factor within the pair cannot compensate for the other at endogenous levels, it remains to be determined if there are truly unique, non-redundant functions of these proteins. To assess this, we overexpressed *Kat6b* mRNA 4-fold above endogenous levels in mice lacking *Kat6a*. We show here that *Kat6b* overexpression can restore all anomalies previously described in *Kat6a* mutant mice, including the 100% penetrant lethality, gene expression anomalies and histone acetylation deficits at histone lysine targets perturbed by loss of KAT6A. These data indicate that KAT6B can completely replace loss of essential KAT6A functions, if expressed at sufficiently high levels, despite KAT6B not normally regulating critical processes dependent on KAT6A.

## Results

### Structure and expression of KAT6A and KAT6B

**Fig. 1:**
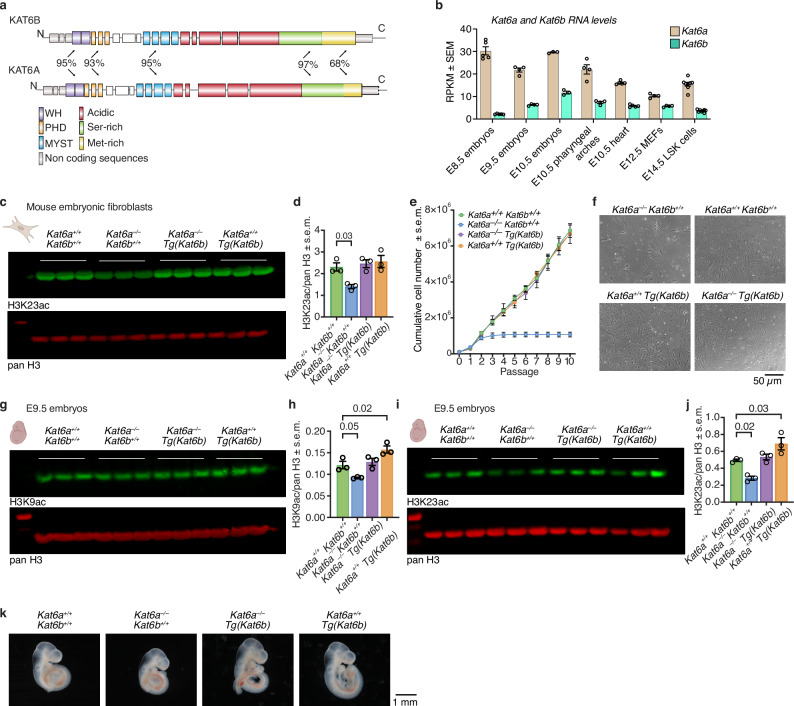
*Kat6b* overexpression restores histone acetylation and cell proliferation in *Kat6a*^–/–^*Tg(Kat6b)* fibroblasts and histone acetylation in *Kat6a*^–/–^*Tg(Kat6b)* embryos. **a** Schematic of *KAT6A* and *KAT6B* mRNA with exon structure and protein domains/regions encoded are shown in colour. White boxes indicate an uncharacterised region. The percentage amino acid sequence similarity between protein domains is indicated. Non-coding exons are indicated in grey. **b** *Kat6a* and *Kat6b* mRNA levels from RNA-seq experiments analysing E8.5 embryos^75^, E9.5 embryos (this work), E10.5 embryos^54^, E10.5 pharyngeal arches^44^, E10.5 heart^75^, E12.5 MEFs^69^, E14.5 LSK cells^76^. **c** Western immunoblot detecting histone H3 acetylated on lysine 23 (H3K23ac) and pan-H3 as a loading control in acid extracted histones from E14.5 mouse embryonic fibroblasts (MEFs). Each lane represents histones from MEFs isolated from an individual E14.5 foetus. 250 ng protein loaded per lane. **d** Quantification of the western immunoblot in (**a**). Each circle represents one lane of the immunoblot in (**a**). **e** Cumulative growth curve of MEFs isolated from *Kat6a*^*+/+*^*Kat6b*^*+/+*^, *Kat6a*^*–/–*^*Kat6b*^*+/+*^, *Kat6a*^*+/+*^*Tg(Kat6b)* and *Kat6a*^*–/–*^*Tg(Kat6b*) E14.5 foetuses, passages 0–10. **f** Representative images of MEFs from *Kat6a*^*+/+*^*Kat6b*^*+/+*^, *Kat6a*^*–/–*^*Kat6b*^*+/+*^, *Kat6a*^*+/+*^*Tg(Kat6b)* and *Kat6a*^*–/–*^*Tg(Kat6b*) E14.5 foetuses at passage 3. Scale bar = 50 μm. **g**–**j** Western immunoblot detecting H3K9ac (**e**) and H3K23ac (**g**) with pan-H3 as a loading on acid extracted histones from E9.5 embryos. Each lane represents histones from an individual E9.5 embryo. 500 ng (**g**) or 250 ng () (**i**) protein loaded per lane. Quantification of (**g**) in (**h**) and of (**i**) in (**j**). **k** Representative images of E9.5 *Kat6b*^*+/+*^*Kat6b*^*+/+*^, *Kat6a*^*–/–*^*Kat6b*^*+/+*^, *Kat6a*^*–/–*^*Tg(Kat6b)* and *Kat6a*^*+/+*^*Tg(Kat6b)* embryos. Scale bar = 1 mm. *N* = 3–8 embryos or foetuses per genotype (**a**), MEF cultures derived from 3 foetuses per genotype (**c**, **d**, **e**, **f**). 3 embryos per genotype (**g**, **h**, **i**, **j**, **k**). Circles represent individual embryos or foetuses (**b**, **d**, **h**, **j**). Data are presented as mean ± s.e.m. and were analysed using a one-way ANOVA with Dunnett post hoc correction (**b**, **g**, **i**) or two-way ANOVA with Sidak post-hoc correction (**c**). Drawings created in BioRender, Bergamasco, M. (2025) https://BioRender.com/m13f247.

KAT6A and KAT6B have identical protein domain structures and high sequence similarity in all functional domains (Fig. 1a). During development, *Kat6a* mRNA is expressed at higher levels than *Kat6b* mRNA (Fig. 1b). Heterozygous mutations in the human *KAT6A* gene result in ARTHS^33,34^, while mutations in the *KAT6B* gene result in the Say-Barber-Biesecker-Young-Simpson variant of Ohdo syndrome (SBBYSS)^35^ or genitopatellar syndrome  (GTPTS)^36^. Despite being clinically distinct, there is notable overlap in the clinical presentation across these disorders (Supplementary Fig. 1b; Supplementary Data 1).

To test if transgenic overexpression of the *Kat6b* gene was able to rescue the phenotypic anomalies resulting from loss of the *Kat6a* gene, we generated transgenic mice overexpressing the *Kat6b* gene from *pBACe3.6* clone *RP23-360F23*. This clone contained all coding exons as well as 21 kb 5 prime and 42 kb 3 prime of the expressed sequence (Supplementary Fig. 1a, b). Seven copies of the *pBACe3.6* inserted into the mouse genome, resulting in the *Tg(Kat6b)* allele, which caused a 4-fold increase in *Kat6b* mRNA levels (Supplementary Fig. 1c). Mice were maintained on an FVB x BALB/c hybrid background as *Kat6b* overexpressing mice were not viable on inbred backgrounds.

To assess the potential of KAT6B to compensate for KAT6A, we crossed *Tg(Kat6b)* heterozygous mice to mice lacking exons 5–9 of *Kat6a* (*Kat6a*^*+/–*^)^17,18^. Embryonic day (E) 9.5 *Tg(Kat6b)* embryos and E14.5 and E18.5, *Tg(Kat6b)* foetuses were present at expected Mendelian ratios (*N* = 15, *p* = 0.2; *N* = 21, *p* = 0.6; *N* = 18, *p* = 0.6; at E9.5, E14.5 and E18.5, respectively; binomial probability *p* values). *Kat6a*^*–/–*^ foetuses were underrepresented at E18.5 (*N* = 16, *p* = 0.8; *N* = 6, *p* = 0.1; *N* = 4, *p* = 0.02; at E9.5, E14.5 and E18.5, respectively). In contrast, *Kat6a*^*–/–*^*Tg(Kat6b)* foetuses that lacked *Kat6a*, but overexpressed *Kat6b*, were obtained at the expected Mendelian ratio (*N* = 9, *p* = 1.0; *N* = 8, *p* = 1.0; *N* = 10, *p* = 1.0; at E9.5, E14.5 and E18.5, respectively).

### *Kat6b* overexpression restored histone acetylation and cell proliferation in *Kat6a*^*–/–*^*Tg(Kat6b)* fibroblasts and histone acetylation in *Kat6a*^*–/–*^*Tg(Kat6b)* embryos

To determine whether KAT6B was capable of compensating for the loss of KAT6A at the biochemical level, histone acetylation levels at previously identified KAT6A lysine targets, H3K9 and H3K23, as well as H3K14, were assessed chromatin-wide by Western blotting in primary mouse embryonic fibroblasts (MEFs) and in whole embryonic day 9.5 (E9.5) embryos.

*Kat6a*^*–/–*^ MEFs had a 40% reduction in acetylation at H3K23 (H3K23ac), relative to wild type controls (*p* = 0.03; Fig. 1c, d). Overexpression of *Kat6b* in *Kat6a*^*–/–*^*Tg(Kat6b)* MEFs returned H3K23ac levels to wild-type levels (Fig. 1c, d). H3K23ac was similar in *Kat6b*^*+/+*^*Tg(Kat6b)* and wild-type *Kat6a*^*+/+*^*Kat6b*^*+/+*^ control MEFs (Fig. 1c, d). Chromatin-wide H3K9 and H3K14 acetylation levels were not affected by KAT6A or KAT6B status in this cell type (Supplementary Fig. 2a–d).

Consistent with previous reports^30,47,48^, *Kat6a*^*–/–*^*Kat6b*^*+/+*^ MEFs underwent cell cycle arrest after only 3 passages in culture (*p* = 0.00007; Fig. 1e, f). Overexpression of *Kat6b* restored cell proliferation in *Kat6a*^*–/–*^*Tg(Kat6b)* MEFs compared to *Kat6a*^*–/–*^*Kat6b*^*+/+*^ cells to levels similar to wild-type *Kat6a*^*+/+*^*Kat6b*^*+/+*^ control MEFs (Fig. 1e, f).

In E9.5 *Kat6a*^*–/–*^*Kat6b*^*+/+*^ embryos, acetylation levels at H3K9 (*p* = 0.046; Fig. 1g, h) and H3K23 (*p* = 0.02; Fig. 1i, j) were 32% and 43% reduced, respectively, compared to *Kat6a*^*+/+*^*Kat6b*^*+/+*^ control embryos. Overexpression of *Kat6b* returned the histone acetylation levels to normal in *Kat6a*^*–/–*^*Tg(Kat6b)* embryos compared to *Kat6a*^*+/+*^*Kat6b*^*+/+*^ control embryos (Fig. 1c, d, g–j). Interestingly, H3K9 and H3K23 were elevated by 41% and 39%, respectively, in *Kat6a*^*+/+*^*Tg(Kat6b)* samples compared to controls (*p* = 0.01–0.03); (Fig. 1g–j). H3K14ac was not affected by KAT6A or KAT6B status in E9.5 embryos (Supplementary Fig. 2e, f). Representative images of E9.5 embryos are shown in (Fig. 1k).

### *Kat6b* overexpression reversed most gene expression changes present in *Kat6a*^*–/–*^ E9.5 embryos

Loss of KAT6A has previously been shown to reduce expression of gene families encoding embryonic patterning transcription factors, most notably *Hox* genes^17^, *Tbx* genes^18^ and *Dlx* genes^44^. To determine how mRNA levels affected by loss of KAT6A were altered when KAT6B was overexpressed, we performed RNA-sequencing of *Kat6a*^*–/–*^*Kat6b*^*+/+*^, *Kat6a*^*+/+*^*Kat6b*^*+/+*^, *Kat6a*^*–/–*^*Tg(Kat6b)* and *Kat6a*^*+/+*^*Tg(Kat6b)* E9.5 embryos (Supplementary Data 2–5). E9.5 was chosen as a timepoint when major patterning genes previously shown to be perturbed by loss of KAT6A are highly expressed.

**Fig. 2:**
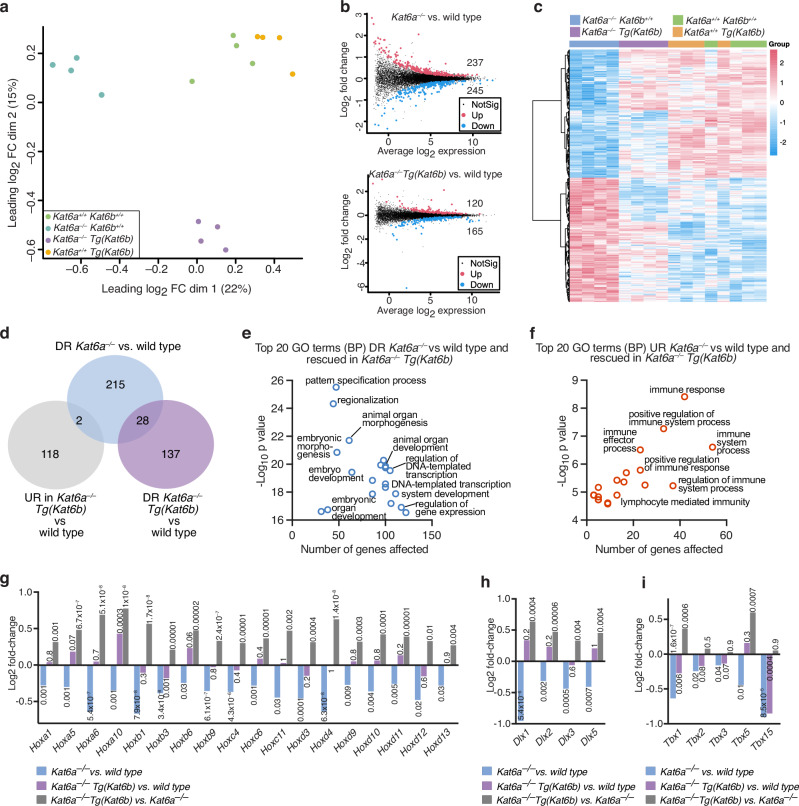
*Kat6b* overexpression reverses gene expression changes present in *Kat6a*^–/–^ E9.5 embryos. **a**–**i** RNA-sequencing data of *Kat6a*^*+/+*^*Kat6b*^*+/+*^, *Kat6a*^*–/–*^*Kat6b*^*+/+*^, *Kat6a*^*+/+*^*Tg(Kat6b)* and *Kat6a*^*–/–*^*Tg(Kat6b*) E9.5 embryos. *N* = 4 embryos per genotype. Data were analysed as described in the ‘methods’ section. A false discovery (FDR) < 0.05 was used as the cut off for significance. **a** Multidimensional scaling plot of the leading gene expression differences between samples in pair-wise comparisons showing *Kat6a*^*+/+*^*Kat6b*^*+/+*^, *Kat6a*^*–/–*^*Kat6b*^*+/+*^, *Kat6a*^*+/+*^*Tg(Kat6b)* and *Kat6a*^*–/–*^*Tg(Kat6b*) E9.5 embryo RNA samples. **b** M (log ratio) and A (mean average) plot showing *Kat6a*^*–/–*^*Kat6b*^*+/+*^ vs. *Kat6a*^*+/+*^*Kat6b*^*+/+*^ and *Kat6a*^*–/–*^*Tg(Kat6b)* vs. *Kat6a*^*+/+*^*Kat6b*^*+/+*^ embryos. The total numbers of upregulated and downregulated genes at FDR < 0.05 are indicated in each comparison. Upregulated genes are represented in red, downregulated in blue and unchanged genes in grey. **c** Heatmap showing genes differentially expressed in the contrast of *Kat6a*^*–/–*^*Kat6b*^*+/+*^ vs. *Kat6a*^*+/+*^*Kat6b*^*+/+*^ embryos but displayed for all genotypes. **d** Venn diagram showing the intersection of genes downregulated (DR) in *Kat6a*^*–/–*^ *Kat6b*^*+/+*^ vs. *Kat6a*^*+/+*^*Kat6b*^*+/+*^ embryos with those downregulated in *Kat6a*^*–/–*^*Tg(Kat6b)* samples vs. *Kat6a*^*+/+*^*Kat6b*^*+/+*^ embryos compared to those upregulated in *Kat6a*^*–/–*^*Tg(Kat6b)* samples vs. *Kat6a*^*+/+*^*Kat6b*^*+/+*^ embryos. **e** Top 20 gene ontology (GO) (BP) terms downregulated in *Kat6a*^*–/–*^*Kat6b*^*+/+*^ vs. *Kat6a*^*+/+*^*Kat6b*^*+/+*^ embryos and rescued in *Kat6a*^*–/–*^*Tg(Kat6b)* when compared to *Kat6a*^*+/+*^*Kat6b*^*+/+*^ embryos. GO terms enriched with *p* < 10^–6^ are shown. **f** Top 20 gene ontology (GO) (BP) terms upregulated in *Kat6a*^*–/–*^*Kat6b*^*+/+*^ vs. *Kat6a*^*+/+*^*Kat6b*^*+/+*^ embryos and rescued in *Kat6a*^*–/–*^*Tg(Kat6b)* when compared to *Kat6a*^*+/+*^*Kat6b*^*+/+*^ embryos. GO terms enriched with *p* < 10^–6^ are shown. **g**–**i** Specific gene families previously reported to be affected by loss of KAT6A^17,18,44^ were examined. Correction for multiple testing was conducted within gene family. FDRs for each comparison are shown above each bar. **g**–**i** Log_2_ fold-change in levels of HOX gene mRNA (**g**), DLX gene mRNA (**h**) and TBX gene mRNA (**i**) across pairwise comparisons *Kat6a*^*–/–*^*Kat6b*^*+/+*^ vs. *Kat6a*^*+/+*^*Kat6b*^*+/+*^ embryos, *Kat6a*^*–/–*^*Tg(Kat6b)* vs. *Kat6a*^*+/+*^*Kat6b*^*+/+*^ embryos and *Kat6a*^*–/–*^*Tg(Kat6b)* vs. *Kat6a*^*–/–*^*Kat6b*^*+/+*^ embryos. FDRs are displayed above or below the bars.

Samples clustered within genotype and segregated between genotypes (Fig. 2a). Interestingly, *Kat6a*^*–/–*^*Tg(Kat6b)* RNA profiles were more closely related to wild-type control profiles in dimension 1, as assessed by multidimensional scaling, compared to *Kat6a*^*–/–*^*Kat6b*^*+/+*^ profiles, but segregated from the other genotypes in dimension 2, indicating that, despite more closely resembling wild-type controls than *Kat6a*^*–/–*^*Kat6b*^*+/+*^samples, *Kat6a*^*–/–*^*Tg(Kat6b)* samples still maintained distinct RNA expression profiles.

A total of 482 genes were differentially expressed in *Kat6a*^*–/–*^*Kat6b*^*+/+*^ vs. *Kat6a*^*+/+*^*Kat6b*^*+/+*^ E9.5 embryos with transcriptome-wide significance (FDR < 0.05); 245 genes were downregulated, and 237 genes were upregulated (Supplementary Data 2). Overexpression of *Kat6b* in *Kat6a*^*–/–*^*Tg(Kat6b)* embryos reduced the number of differentially expressed genes compared to *Kat6a*^*+/+*^*Kat6b*^*+/+*^ embryos to 285 genes, 165 downregulated and 120 upregulated (Fig. 2b; Supplementary Data 3). Other pairwise comparisons are shown in Supplementary Fig. 3a and Supplementary Data 4–7. A heat map shows that *Kat6a*^*–/–*^*Tg(Kat6b)* embryos clustered more closely with wild-type *Kat6a*^*+/+*^*Kat6b*^*+/+*^control embryos than *Kat6a*^*–/–*^*Kat6b*^*+/+*^samples (Fig. 2c).

Of the 245 genes downregulated in *Kat6a*^*–/–*^*Kat6b*^*+/+*^ vs. *Kat6a*^*+/+*^*Kat6b*^*+/+*^, only 28 genes were similarly downregulated in *Kat6a*^*–/–*^*Tg(Kat6b)* samples (Fig. 2d; Supplementary Data 2 and 3), while two of the rescued genes, (*Cdx2, Hoxa10*), were overcompensated, i.e., expressed at higher levels in *Kat6a*^*–/–*^*Tg(Kat6b)* vs. *Kat6a*^*+/+*^*Kat6b*^*+/+*^ E9.5 embryos.

The genes downregulated in *Kat6a*^*–/–*^*Kat6b*^*+/+*^ vs. *Kat6a*^*+/+*^*Kat6b*^*+/+*^ E9.5 embryos were enriched in gene ontology (BP) terms relating to embryonic development, embryo patterning and DNA binding transcription factors regulating these processes (Supplementary Fig. 3b). The genes that displayed a rescue of mRNA levels from downregulated in *Kat6a*^*–/–*^*Kat6b*^*+/+*^ vs. *Kat6a*^*+/+*^*Kat6b*^*+/+*^ E9.5 embryos to normal levels in *Kat6b* in *Kat6a*^*–/–*^*Tg(Kat6b)* vs. *Kat6a*^*+/+*^*Kat6b*^*+/+*^ E9.5 embryos were enriched for similar developmental processes (Fig. 2e, f). Genes upregulated in *Kat6a*^*–/–*^*Kat6b*^*+/+*^ vs. *Kat6a*^*+/+*^*Kat6b*^*+/+*^ E9.5 embryos showed no specificity to embryonic development (Supplementary Fig. 3c).

Among the rescued genes were the major families of embryonic patterning genes previously reported as downregulated in *Kat6a*^*–/–*^ vs. *Kat6a*^*+/+*^ embryos; *Hox* genes^17^, *Tbx* genes^18^ and *Dlx* genes^44^. The major downregulation of *Hox* genes observed in *Kat6a*^*–/–*^*Kat6b*^*+/+*^ vs. *Kat6a*^*+/+*^*Kat6b*^*+/+*^ E9.5 embryos (FDR < 10^−6^ to 0.03 within gene family; Supplementary Fig. 3d, e; Data 8) was no longer present in *Kat6a*^*–/–*^*Tg(Kat6b)* vs. *Kat6a*^*+/+*^*Kat6b*^*+/+*^ E9.5 embryos (Fig. 2g), indicating that KAT6B can promote expression of *Hox* genes in the absence of KAT6A. Only *Hoxb3* mRNA levels were not fully returned to wild-type levels, but still were significantly elevated in *Kat6a*^*–/–*^*Tg(Kat6b)* vs. *Kat6a*^*–/–*^*Kat6b*^*+/+*^ embryos (FDR = 10^−5^; Fig. 2g), indicating a partial rescue. Similarly, *Dlx* genes downregulated in *Kat6a*^*–/–*^*Kat6b*^*+/+*^ vs. *Kat6a*^*+/+*^*Kat6b*^*+/+*^ embryos (FDR = 6 × 10^−5^ to 0.004 within gene family; Supplementary Fig. 3d, e, Data 8) were no longer downregulated in *Kat6a*^*–/–*^*Tg(Kat6b)* vs. *Kat6a*^*+/+*^*Kat6b*^*+/+*^ (FDR = 0.2 to 1; Fig. 2h). *Tbx* genes were downregulated *Kat6a*^*–/–*^*Kat6b*^*+/+*^ vs. *Kat6a*^*+/+*^*Kat6b*^*+/+*^ embryos (FDR < 10^−6^ to 0.04 within gene family; Supplementary Data 8) and were upregulated in *Kat6a*^*–/–*^*Tg(Kat6b)* vs. *Kat6a*^*–/–*^*Kat6b*^*+/+*^, except *Tbx15* (Fig. 2i; Supplementary Fig. 3d, e, Supplementary Data 8). This upregulation returned *Tbx2, Tbx3 and Tbx5* mRNA levels in *Kat6a*^*–/–*^*Tg(Kat6b)* embryos to comparable levels to *Kat6a*^*+/+*^*Kat6b*^*+/+*^ embryos (FDR = 0.06 to 0.3). The mRNA levels of *Tbx1* were significantly elevated in *Kat6a*^*–/–*^*Tg(Kat6b)* embryos above *Kat6a*^*–/–*^*Kat6b*^*+/+*^ embryos (FDR = 0.0006), but still lower than in *Kat6a*^*+/+*^*Kat6b*^*+/+*^ embryos (FDR = 0.006), indicating a partial rescue. Overall, 217 (89%) of 245 genes downregulated and 222 (94%) of the 237 upregulated genes in *Kat6a*^*–/–*^*Kat6b*^*+/+*^ vs. *Kat6a*^*+/+*^*Kat6b*^*+/+*^ E9.5 embryos were rescued by overexpression of *Kat6b* in *Kat6a*^*–/–*^*Tg(Kat6b)* vs. *Kat6a*^*+/+*^*Kat6b*^*+/+*^ embryos, indicating that KAT6B overexpression substantially restores expression of genes, which were reduced in the absence of KAT6A (Fig. 2d; Supplementary Fig. 4a, b). Examining the gene expression not rescued in more detail we found the majority of these 28 downregulated and 15 upregulated genes showed relatively small differential gene expression changes, less than 2-fold, (Supplementary Fig. 4c, d), which were associated with GO terms relating to transcription in the case of downregulated genes and cell adhesion in the case of upregulated genes (Supplementary Fig. 4e, f). Some 236 genes were uniquely differentially expressed in *Kat6a*^*–/–*^*Tg(Kat6b)* vs. *Kat6a*^*+/+*^*Kat6b*^*+/+*^ embryos (Supplementary Fig. 4g–i) and a comparatively small number of genes (75) were differentially expressed when embryos overexpressing *Kat6b* were compared to wild type (Supplementary Fig. 4j–l).

### *Kat6b* overexpression restored transplantable haematopoietic stem cells that are absent in *Kat6a*^*–/–*^ mice

KAT6A is essential for development of HSCs. Germline deletion of the *Kat6a* gene results in a complete failure to form definitive HSCs during foetal development^42,43^, and deletion of *Kat6a* in adult HSCs causes their complete loss^49^.

**Fig. 3:**
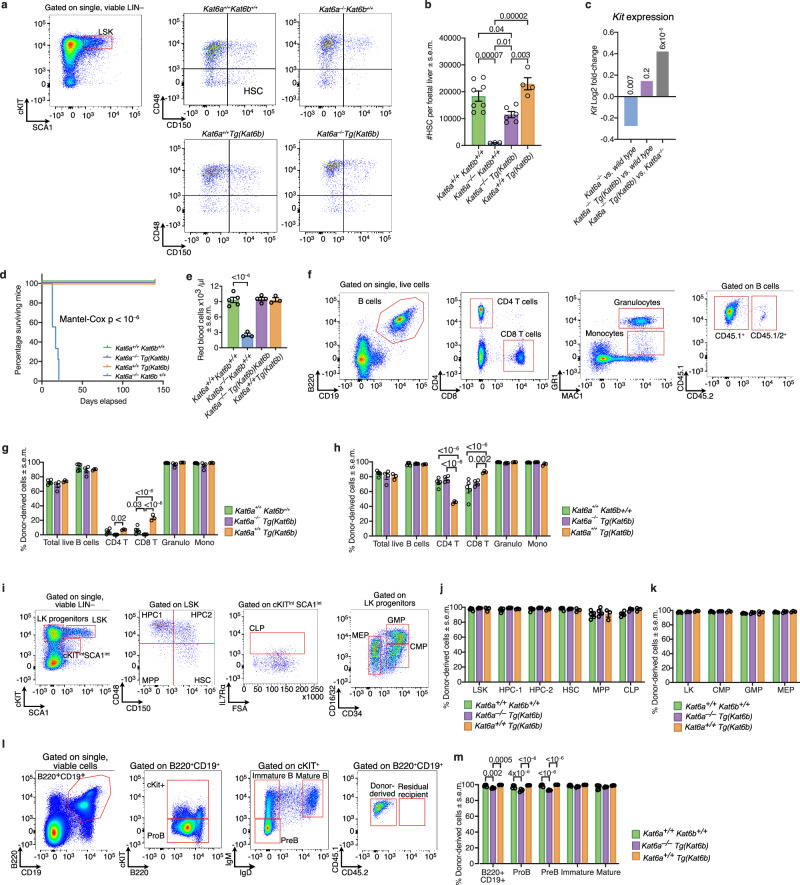
*Kat6b* overexpression restores transplantable haematopoietic stem cells that are absent in *Kat6a*^–/–^ mice. **a** Flow cytometry gating strategy for the assessment of haematopoietic stem cells (HSCs) in E14.5 foetal livers. HSCs were identified as the CD48^–^CD150^+^ cell population within the lineage marker negative (LIN^–^) cKIT and SCA1 positive cell population (LSK; gated on single, viable cells, lacking expression of lineage (LIN) markers B220, CD19, CD4, CD8, GR1 and TER119 and expressing SCA1 and cKIT). Representative plots for HSCs are shown for each genotype. **b** Total number of HSCs per foetal liver of *N* = 3 *Kat6a*^*–/–*^*Kat6b*^*+/+*^, *N* = 8 *Kat6a*^*+/+*^*Kat6b*^*+/+*^, *N* = 6 *Kat6a*^*–/–*^*Tg(Kat6b)* and *N* = 4 *Kat6a*^*+/+*^*Tg(Kat6b)* foetuses. **c** Log_2_ fold-change in *Kit* mRNA levels in E9.5 embryos as assessed by RNA-sequencing across the comparisons *Kat6a*^*–/–*^*Kat6b*^*+/+*^ vs. *Kat6a*^*+/+*^*Kat6b*^*+/+*^, *Kat6a*^*–/–*^*Tg(Kat6b)* vs. *Kat6a*^*+/+*^*Kat6b*^*+/+*^ and *Kat6a*^*–/–*^*Tg(Kat6b)* vs. *Kat6a*^*–/–*^*Kat6b*^*+/+*^ embryos. *N* = 4 embryos per genotype. The FDRs are shown above the bars. Data were analysed as described in the ‘methods’ section under *RNA sequencing and analysis*. **d** Percentage survival of irradiated recipient mice after transplantation of 1 × 10^6^ foetal liver cells from *N* = 3 *Kat6a*^*–/–*^*Kat6b*^*+/+*^, *N* = 4 *Kat6a*^*–/–*^*Tg(Kat6b)*, *N* = 3 *Kat6a*^*+/+*^*TgKat6b)* or *N* = 5 *Kat6a*^*+/+*^*Kat6b*^*+/+*^ E14.5 mouse foetuses; each foetal liver sample was transplanted into 3 lethally irradiated recipients. Data are displayed in a Kaplan-Meier plot and were analysed using a Mantel-Cox test. **e** Automated haematological analyser (ADVIA) assessment of red blood cells per μl peripheral blood in recipient mice of E14.5 foetal liver donor cells from *N* = 5 *Kat6a*^*+/+*^*Kat6b*^*+/+*^, *N* = 3 *Kat6a*^*+/+*^*Tg(Kat6b)*, *N* = 4 *Kat6a*^*–/–*^*Tg(Kat6b)* or *N* = 3 *Kat6a*^*–/–*^*Kat6b*^*+/+*^ foetuses, at a time 3–4 weeks after transplantation when the recipient mice of *Kat6a*^*–/–*^*Kat6b*^*+/+*^ donor cells in each set reached the ethical end-point. Each foetal liver sample was transplanted into 3 recipient mice. Each circle represents the average of the recipient mice of an individual foetal liver donor. Data are displayed as mean ± s.e.m. and were analysed using a one-way ANOVA with Dunnett post-hoc correction. **f** Gating strategy for the assessment of peripheral blood of foetal liver transplant recipient mice. B cells were defined as B220^+^CD19^+^, T cells as CD4^+^ or CD8^+^, granulocytes as GR1^hi^ MAC1^+^ and monocytes as GR1^lo^MAC1^+^. Foetal liver donor-derived cells (CD45.1^+^) were distinguished from residual recipient cells (CD45.1/2^+^). **g**, **h** Contribution of foetal liver (donor)-derived cells to peripheral blood populations at 4 weeks (**f**) and 20 weeks (**g**) post transplantation of the recipients described in (**e**). **i** Gating strategy for the assessment of stem and progenitor cells in the bone marrow of foetal liver transplant recipient mice. Cell types as indicated on the plots were distinguished by CD48 vs. CD150, CD34 vs. CD16.32 or IL7Rα expression. (j–k) Contribution of foetal liver (donor)-derived cells to stem and progenitor populations in the bone marrow of foetal liver cell recipients at 20 weeks post-transplantation in CD48 vs. CD150 (**i**), CD34 vs CD16/32 (**j**) cell populations. **l** Gating strategy for the assessment of B cell progenitors in the bone marrow of foetal liver transplant recipient mice. Cells were gated on single, viable cells co-expressing B220 and CD19 and distinguished by cKIT, IgM and IgD expression to identify the cell populations indicated in the plots. **m** Contribution of foetal liver (donor)-derived cells to bone marrow B cell progenitors 20 weeks post-transplant. Circles represent individual foetuses (**b**) or the mean of three recipients of a single foetal liver donor (**g**, **h**, **j**, **k**, **m**). Data are presented as mean ± s.e.m. and were analysed using a one-way ANOVA (**b**) or two-way ANOVA with Tukey post hoc correction (**g**, **h**, **j**, **k**, **m**). HSCs, haematopoietic stem cells; LK; lineage marker negative cKIT^+^ cells; LSK, lineage marker negative SCA1^+^cKIT^+^ cells; HPC-1 (haematopoietic progenitor 1), HPC-2 (haematopoietic progenitor 2), MPP (multipotent progenitor), CLP (common lymphoid progenitor), CMP (common myeloid progenitor), GMP (granulocyte macrophage progenitors), MEP (megakaryocyte/erythroid progenitor). Granulo (granulocytes), Mono (monocytes); superscript: int, intermediate.

The numbers of HSCs in *Kat6a*^*+/+*^*Kat6b*^*+/+*^, *Kat6a*^*+/+*^*Tg(Kat6b)*, *Kat6a*^*–/–*^*Kat6b*^*+/+*^ and *Kat6a*^*–/–*^*Tg(Kat6b)* foetal livers were assessed by flow cytometry (Fig. 3a, b). Analysis was performed at E14.5, at the peak of foetal haematopoiesis in mice^50,51^ and HSCs were identified as CD48^–^ CD150^+^, as described^52^. As previously described^42,43^, *Kat6a*^*–/–*^*Kat6b*^*+/+*^ foetal livers showed a 95% reduction in cells with an HSC cell surface phenotype compared to wild-type *Kat6a*^*+/+*^*Kat6b*^*+/+*^ control foetal livers (*p* = 0.00007; Fig. 3b). Overexpression of *Kat6b* in *Kat6a*^*–/–*^*Tg(Kat6b)* foetuses elevated the number of HSC 11.5-fold, compared to *Kat6a*^*–/–*^*Kat6b*^*+/+*^ foetuses (*p* = 0.01; Fig. 3b), however, HSCs were still reduced in *Kat6a*^*–/–*^*Tg(Kat6b)* foetuses compared to *Kat6a*^*+/+*^*Kat6b*^*+/+*^ control foetuses (*p* = 0.04; Fig. 3b). Consistently, expression of the haematopoietic marker gene *Kit*^50,53^ was downregulated in *Kat6a*^*–/–*^*Kat6b*^*+/+*^ vs. *Kat6a*^*+/+*^*Kat6b*^*+/+*^ E9.5 embryos (FDR = 0.007; Fig. 3c), rescued in *Kat6a*^*–/–*^*Tg(Kat6b)* vs. wild-type embryos (FDR = 0.2) and upregulated in *Kat6a*^*–/–*^*Tg(Kat6b)* vs. *Kat6a*^*–/–*^*Kat6b*^*+/+*^ embryos (FDR = 6 × 10^–5^; Fig. 3c).

To assess the effect of KAT6B overexpression on the function of HSCs lacking KAT6A, 1 × 10^6^ foetal liver cells were transplanted into irradiated recipients, as previously described^43^. Recipients of *Kat6a*^*–/–*^*Kat6b*^*+/+*^ foetal liver cells reached the ethical endpoint within 21 days (*p* < 10^–6^; Fig. 3d), exhibiting anaemia (*p* = 10^–6^; Fig. 3e) and minimal contribution of the foetal liver donor cells to the recipient peripheral blood (Supplementary Fig. 5a). In contrast, recipients of *Kat6a*^*–/–*^*Tg(Kat6b)* cells survived beyond 150 days, as did recipients of wild type or *Kat6a*^*+/+*^*Tg(Kat6b)* cells (Fig. 3d). At 4 weeks post-transplantation, *Kat6a*^*–/–*^*Tg(Kat6b)* donor cell contribution to peripheral blood cells was similar to *Kat6a*^*+/+*^*Kat6b*^*+/+*^ donor cells, except for a reduction in CD8 T cells (*p* = 0.03; Fig. 3f, g). At 20 weeks post-transplantation, contribution to all peripheral cell types was identical between *Kat6a*^*–/–*^*Tg(Kat6b)* and *Kat6a*^*+/+*^*Kat6b*^*+/+*^ donor cells (Fig. 3h). Interestingly, compared to wild type controls, *Kat6a*^*+/+*^*Tg(Kat6b)* donor cells showed an elevated contribution to CD8 T cells at 4 weeks, (*p* < 10^–6^; Fig. 3g) and 20 weeks (*p* = 0.00005; Fig. 3h), with a reduced contribution to CD4 T cells at 20 weeks (*p* < 10^–6^; Fig. 3h). In the bone marrow at 20 weeks post-transplantation, recipients of *Kat6a*^*–/–*^*Tg(Kat6b)*, *Kat6a*^*+/+*^*Kat6b*^*+/+*^ and *Kat6a*^*+/+*^*Tg(Kat6b)* donor cells showed comparable contributions to all stem and progenitor cell populations, except for a small reduction (less than 10%) in *Kat6a*^*–/–*^*Tg(Kat6b)* contribution to the B220^+^CD19^+^, ProB and PreB cell populations (*p* < 10^–6^ to 0.002; Fig. 3i–m). No difference was observed between recipients of *Kat6a*^*+/+*^*Kat6b*^*+/+*^ and *Kat6b*^*+/+*^*Tg(Kat6b)* recipients across bone marrow populations analysed (Fig. 3i–m).

### *Kat6b* overexpression rescued the anterior homeotic transformation seen in *Kat6a*^*–/–*^ mice

**Fig. 4:**
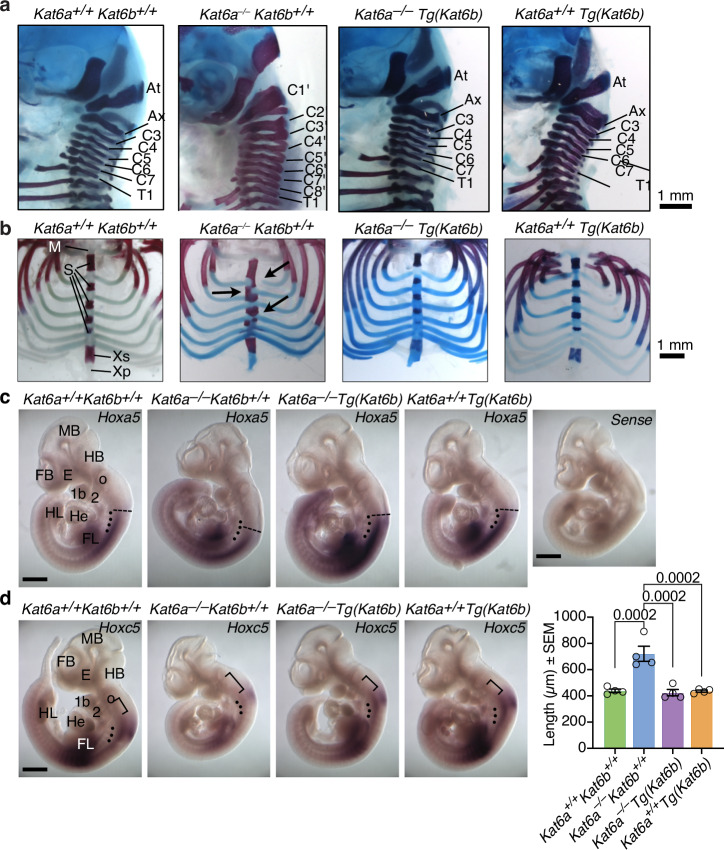
*Kat6b* overexpression rescues the anterior homeotic transformation seen in *Kat6a*^–/–^ mice. **a**, **b** Alizarin red (bone) and Alcian blue (cartilage) stained skeletal preparations of *N* = 9 *Kat6a*^*+/+*^*Kat6b*^*+/+*^, 1 *Kat6a*^*–/–*^*Kat6b*^*+/+*^, 4 *Kat6a*^*–/–*^*Tg(Kat6b)* and 5 *Kat6a*^*+/+*^*Tg(Kat6b)* E18.5 foetuses with labelled cervical vertebrae (**a**) and sternum (**b**), arrow indicates abnormal rib attachment (**b**). Note that embryos homozygous for this allele of *Kat6a* rarely survive until E18.5. Representative images are shown. Representative images of RNA/RNA whole-mount situ hybridisation to detect *Hoxa5* (**c**) and *Hoxc5* (**d**) mRNA in *Kat6a*^*+/+*^*Kat6b*^*+/+*^*, Kat6a*^*–/–*^*Kat6b*^*+/+*^*, Kat6a*^*–/–*^*Tg(Kat6b)* and *Kat6a*^*+/+*^*Tg(Kat6b)* E10.5 embryos, with anterior expression boundary indicated. Note no staining using the sense control probe (*n* = 3 wild type embryos). The 3–4 most cranial somites are indicated by a dot (**c**, **d**). The anterior expression boundary of *Hoxa5* is indicated by a stippled line (**c**). The distance between the anterior boundary of the *Hoxc5* expression domain in the neural tube and the caudal boundary of the otic vesicle is indicated by a bracket (**d**). The distance was measured and is displayed in the bar graph in (**d**). *Hoxa5* *N* = 3, *Kat6a*^*+/+*^*Kat6b*^*+/+*^, *N* = 3, *Kat6a*^*–/–*^*Kat6b*^*+/+*^, *N* = 3, *Kat6a*^*–/–*^*Tg(Kat6b)* and *N* = 3, *Kat6a*^*+/+*^*Tg(Kat6b)* were used. *Hoxc5* *N* = 4, *Kat6a*^*+/+*^*Kat6b*^*+/+*^, *N* = 4, *Kat6a*^*–/–*^*Kat6b*^*+/+*^, *N* = 4, *Kat6a*^*–/–*^*Tg(Kat6b)* and *N* = 4, *Kat6a*^*+/+*^*Tg(Kat6b)* were used. This experiment was repeated a second time with each probe with *N* = 3 embryos per genotype. Data are presented as means ± s.e.m. and were analysed by one-way ANOVA with Tukey’s multiple comparison test. Circles in the bar graph (**d**) represent individual embryos. 1b, mandibular region of the first pharyngeal arch; 2, second pharyngeal arch; At, atlas = 1st cervical vertebra, C1; Ax, axis = 2nd cervical vertebra, C2; C1 to C7, 1st to 7th cervical vertebrae; C1’ to C8’, 1st to 8th abnormal *Kat6a*^*–/–*^*Kat6b*^*+/+*^ cervical vertebrae, whereby C8’ is supernumerary; E eye, FB forebrain, FL forelimb, HB hindbrain, He heart, HL hindlimb, M manubrium, MB midbrain, o otic vesicle, S sternebrae, T1 1st thoracic vertebra (rib bearing). Xp xiphoid process, Xs xiphisternum. Scale bar = 1 mm (**a**, **b**), 600 µm (**c**), 680 µm (**d**).

**Fig. 5:**
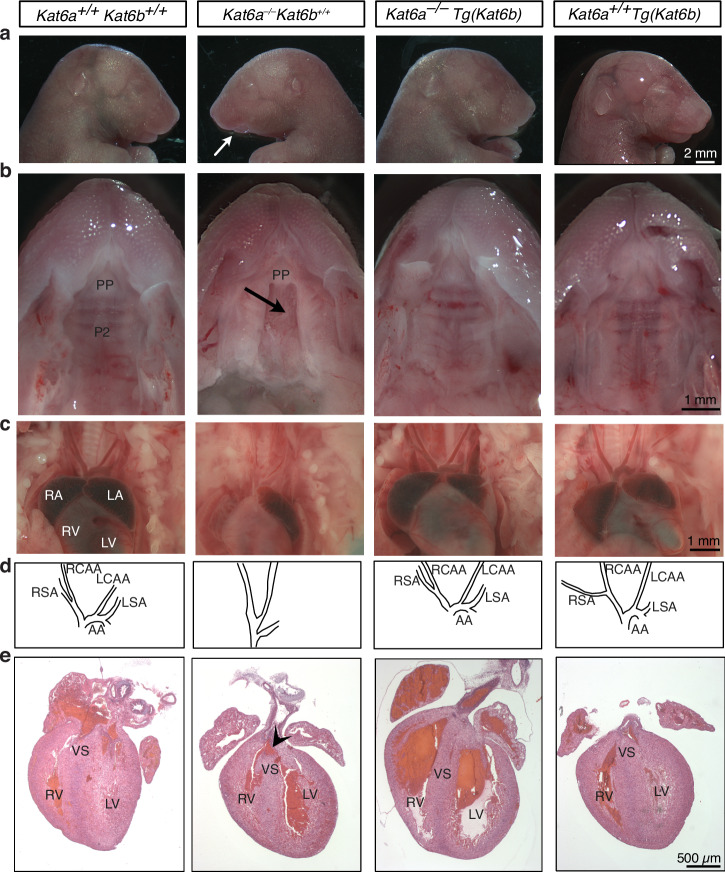
Kat6b overexpression rescues the cleft palate, aortic arch and cardiac defects observed in *Kat6a*^–/–^ mice. **a**–**e** Examination of *N* = 7 *Kat6a*^*+/+*^*Kat6b*^*+/+*^, *N* = 1 *Kat6a*^*–/–*^*Kat6b*^*+/+*^, *N* = 5 *Kat6a*^*–/–*^*Tg(Kat6b)* and *N* = 6 *Kat6a*^*+/+*^*Tg(Kat6b)* foetuses at E18.5 during dissection and by histopathology. Note that embryos homozygous for this allele of KAT6a rarely survive until E18.5. **a** Lateral view of the head and neck of E18.5 *Kat6a*^*+/+*^*Kat6b*^*+/+*^*, Kat6a*^*–/–*^*Kat6b*^*+/+*^*, Kat6a*^*–/–*^*Tg(Kat6b)* and *Kat6a*^*+/+*^*Tg(Kat6b)* foetuses. The arrow indicates an underdeveloped lower jaw in the *Kat6a*^*–/–*^*Kat6b*^*+/+*^ mouse. **b** Ventral view of the palate of E18.5 *Kat6a*^*+/+*^*Kat6b*^*+/+*^*, Kat6a*^*–/–*^*Kat6b*^*+/+*^*, Kat6a*^*–/–*^*Tg(Kat6b)* and *Kat6a*^*+/+*^*Tg(Kat6b)* foetuses. The arrow indicates a cleft palate in the *Kat6a*^*–/–*^*Kat6b*^*+/+*^ mouse. **c**, **d** Images of the heart and aortic arch of *Kat6a*^*+/+*^*Kat6b*^*+/+*^*, Kat6a*^*–/–*^*Kat6b*^*+/+*^*, Kat6a*^*–/–*^*Tg(Kat6b)* and *Kat6a*^*+/+*^*Tg(Kat6b)* E18.5 foetuses (**c**). Traces of the aortic arch and arteries (**d**). **e** H&E sections of the hearts of *Kat6a*^*+/+*^*Kat6b*^*+/+*^*, Kat6a*^*–/–*^*Kat6b*^*+/+*^*, Kat6a*^*–/–*^*Tg(Kat6b)* and *Kat6a*^*+/+*^*Tg(Kat6b)* E18.5 foetuses. Arrowhead indicates the ventricular septal defect in *Kat6a*^*–/–*^*Kat6b*^*+/+*^ animals. AA aortic arch, LA left atrium, LCCA left common carotid artery, LSA left subclavian artery, LV left ventricle, P2 secondary palate, PP primary palate, RA right atrium, RCCA right common carotid artery, RSA right subclavian artery, RV right ventricle, VS ventricular septum. Scale bars are 2 mm (**a**), 1 mm (**b**, **c**), 500 μm (**e**).

Germline deletion of the *Kat6a* gene results in duplication of the first cervical vertebra, the atlas, accompanied by an extensive and complete homeotic transformation of 19 body segments. This anterior homeotic transformation is caused by a posterior shift of the anterior expression boundary and expression levels of *Hox* genes, including *Hoxa3, Hoxa4, Hoxb3* and *Hoxb4*^17^. Consistent with this previous work^17^ and the reduced *Hox* gene expression in E9.5 embryos shown here (Fig. 2g), *Kat6a*^*–/–*^*Kat6b*^*+/+*^ pups displayed an anterior homeotic transformation at E18.5 (Fig. 4a; Supplementary Fig. 6). In addition, we saw disrupted sternum sections, sternebrae and incorrect alignment of ribs at the sternum in *Kat6a*^*–/–*^*Kat6b*^*+/+*^ pups (Fig. 4b). Congruent with a rescue of the *Hox* gene expression profile at E9.5 (Fig. 2g), the vertebral segment identity, sternebrae structure and rib attachment were rescued in *Kat6a*^*–/–*^*Tg(Kat6b)* pups at E18.5 (Fig. 4a, b). The cervical vertebrae, sternebrae and rib attachment were normal in *Kat6a*^*+/+*^*Kat6b*^*+/+*^ and *Kat6a*^*+/+*^*Tg(Kat6b)* pups (Fig. 5a, b). No obvious effects of KAT6A or KAT6B genotype was observed on lumbar and sacral elements (Supplementary Fig. 7).

To examine the pattern of *Hox* gene expression more closely we performed whole mount in situ hybridisation. We have previously shown that not only is *Hox* gene expression down regulated in embryos but also that the anterior boundary is shifted posteriorly, which leads to an anterior homeotic transformation^17^. We examined the expression patterns of *Hoxa3*, *Hoxa5* and *Hoxc5*, as we have previously shown that the expression of these genes most clearly demonstrates the effect of *Kat6a* loss on the anterior boundary of *Hox* gene expression^17,54^. As previously reported, the *Hoxa5* and *Hoxc5* expression is shifted posteriorly and expressed at lower levels in *Kat6a*^*–/–*^*Kat6b*^*+/+*^ embryos (Fig. 4c, d; Supplementary Fig. 7). The anterior boundary and level of expression of both *Hoxa5* and *Hoxc5* in rescued in *Kat6a*^*–/–*^*Tg(Kat6b)* embryos to the wild type position. Interestingly, the anterior boundary of *Hoxa5* expression in *Kat6a*^*+/+*^*Tg(Kat6b)* embryos is not different to wild type littermate embryos. Similarly, *Hoxa3* expression, reduced and posteriorly shifted in *Kat6a*^*–/–*^*Kat6b*^*+/+*^ embryos, is restored in *Kat6a*^*–/–*^*Tg(Kat6b)* embryos to the wild type pattern of expression (Supplementary Fig. 7).

### *Kat6b* overexpression rescued the cleft palate, cardiac and aortic arch defects observed in *Kat6a*^*–/–*^ mice

*Kat6a*^*–/–*^ mice have previously been shown to have cleft palate^18,44^, ventricular septum defects^18,45^ and aortic arch defects^18^. Congruent with these previous studies, cleft palate (Fig. 5a, b), aortic arch defects (Fig. 5c, d) and ventricular septal defects (Fig. 5e) were observed in *Kat6a*^*–/–*^*Kat6b*^*+/+*^ mice (Fig. 5a–e). Overexpression of *Kat6b* in *Kat6a*^*–/–*^*Tg(Kat6b)* mice rescued each of these major phenotypic anomalies (Fig. 5a–e).

### *Kat6b* overexpression rescued the pattern of H3K23 acetylation at *Kat6a* target loci

**Fig. 6:**
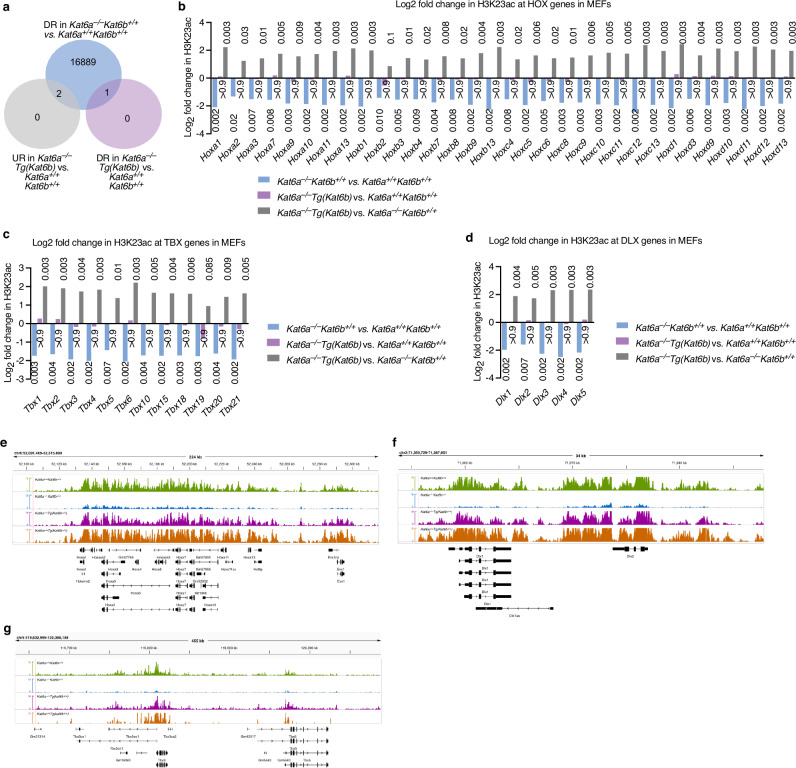
Decrease in H3K23ac in *Kat6a*^–/–^ cells restored to normal in *Kat6a*^–/–^*Tg(Kat6b)* cells. **a**–**g** CUT&Tag results detecting histone H3 lysine 23 acetylation in (H3K23ac) in primary mouse embryonic fibroblasts isolated from E14.5 *Kat6a*^*+/+*^*Kat6b*^*+/+*^*, Kat6a*^*–/–*^*Kat6b*^*+/+*^*, Kat6a*^*–/–*^*Tg(Kat6b)* and *Kat6a*^*+/+*^*Tg(Kat6b)* E10.5 foetuses. *N* = 4 foetuses per genotype. Data were analysed as described in the ‘methods’ section. A false discovery rate (FDR) of less than 0.05 was considered significant. **a** Venn diagram showing number of genes with reduced H3K23ac levels in the indicated samples compared to wild type controls. In comparison to wild type cells *Kat6a*^*–/–*^ cells show a global reduction in H3K23ac which is restored to normal in *Kat6a*^*–/–*^*Tg(Kat6b)* cells. **b**–**d** Log_2_ fold changes in H3K23ac levels at HOX genes (**b**), TBX genes (**c**) and DLX genes (**e**). Note that the reduction in H3K23ac in *Kat6a*^*–/–*^ MEFs is restored to normal in *Kat6a*^*–/–*^*Tg(Kat6b)* MEFs. The FDR is indicated above or below each bar. **e**–**g** Read depth plots of H3K23ac in the HOXA gene cluster (**e**), at the *Dlx1/Dlx2* locus (**f**) and at the *Tbx3/Tbx5* locus. Note that the characteristic distribution of H3K23ac in wild type cells is restored in *Kat6a*^*–/–*^*Tg(Kat6b)* cells.

Western blot analysis showed that loss of KAT6A function results in a global reduction in H3K23ac in E9.5 embryos and MEFs, and that this is restored in the *Kat6a*^*–/–*^*Tg(Kat6b)* condition. To examine specific genomic loci in more detail, in particular *Hox* clusters, *Tbx* and *Dlx* genes, we performed Cleavage Under Targets and Tagmentation (CUT&Tag) sequencing for H3K23ac. Since H3K23ac is one of the most abundant histone acetylation modifications and is widespread throughout the genome we used a *Drosophila* spike-in control to allow direct comparison between samples. Across samples H3K23ac showed a characteristic peak at the transcription start site (TSS) and a large difference between *Kat6a*^*–/–*^ vs. *Kat6b*^*+/+*^ in a multidimensional scaling plot (Supplementary Fig. 8a–c; Data 9). As expected, H3K23ac levels were reduced across the genome at 16889 loci in *Kat6a*^*–/–*^ samples compared to wild type controls (Fig. 6a; Supplementary Fig. 8d). This reduction in H3K23ac was completely reversed in cells prepared from *Kat6a*^*–/–*^*Tg(Kat6b)* embryos (Fig. 6a; Supplementary Fig. 8e–g; Data 10–12). Specifically examining all *Hox* genes we found that H3K23ac was reduced in *Kat6a*^*–/–*^ cells compared to wild type and normal levels were restored in *Kat6a*^*–/–*^*Tg(Kat6b)* cells (Fig. 6b). Similarly at both *Tbx* and *Dlx* genes, H3K23ac was reduced in *Kat6a*^*–/–*^ cells and restored in *Kat6a*^*–/–*^*Tg(Kat6b)* cells (Fig. 6c, d). Mapping individual reads across *Hox*, *Tbx* and *Dlx* loci (Fig. 6e–g) showed that H3K23ac was distributed across the gene body with distinctive peaks at promoters and other genomic features (Supplementary Fig. 8c). Unfortunately, we were unable to correlate the distribution of KAT6A or KAT6B with H3K23ac peaks as no CUT&Tag capable antibodies with the necessary specificity are available. The wild type pattern of peaks was faithfully reproduced in *Kat6a*^*–/–*^*Tg(Kat6b)* cells (Fig. 6e–g).

### *Kat6b* overexpression rescued the lethality of *Kat6a*^*–/–*^ mice

Overexpression of KAT6B rescued histone acetylation, gene expression patterns, transplantable HSCs, segment identity defects, heart and aortic arch defects and cleft palate previously described in *Kat6a*^*–/–*^*Kat6b*^*+/+*^ mice. This demonstrates a complete rescue of all previously described developmental anomalies resulting from loss of KAT6A, including those that were incompatible with survival. To assess if perinatal lethality was rescued, *Kat6a*^*–/–*^ *Tg(Kat6b)* mice were allowed to develop to birth and were monitored in early life.

**Fig. 7:**
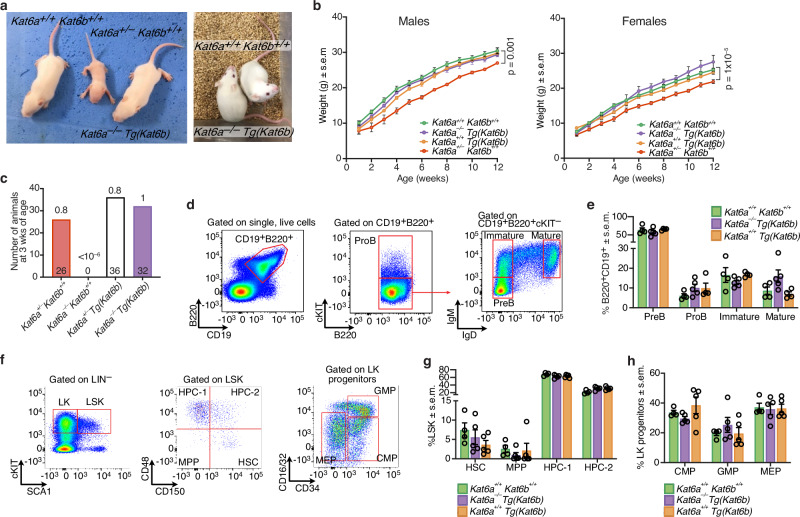
*Kat6b* overexpression rescues the perinatal lethality of *Kat6a*^–/–^ mice. **a** Representative photographs of wild type, *Kat6a*^*+/–*^*Kat6b*^*+/+*^ and *Kat6a*^*–/–*^*Tg(Kat6b)* mice at postnatal day 7 (left) and *Kat6a*^*+/+*^*Kat6b*^*+/+*^ and *Kat6a*^*–/–*^*Tg(Kat6b)* mice at 12 weeks of age (right). **b** Weights of *Kat6a*^*+/+*^*Kat6b*^*+/+*^ (23 female; 16 male), *Kat6a*^*+/–*^*Kat6b*^*+/+*^ (16 female; 4 male), *Kat6a*^*–/–*^*Tg(Kat6b)* (4 female; 4 male) and *Kat6a*^*+/+*^*Tg(Kat6b)* (4 female; 4 male) mice from postnatal week 1 to week 12. Data presented as mean ± s.e.m. and were analysed using a two-way ANOVA with Holm-Sidak post-hoc correction. **c** Genotypes at 3 weeks of age of offspring (*N* = 94) of matings with the parental genotypes *Kat6a*^*+/–*^*Kat6b*^*+/+*^ x *Kat6a*^*–/–*^*Tg(Kat6b)*. The observed genotype frequency was compared to the expected Mendelian frequencies, *p* values of the binomial probability testing if a genotype was observed differed from the expected frequency are shown (two sided). **d**–**h** Flow cytometry analysis of the bone marrow of *N* = 4 adult *Kat6a*^*+/+*^*Kat6b*^*+/+*^, 5 *Kat6a*^*–/–*^*Tg(Kat6b)* and 5 *Kat6a*^*+/+*^*Tg(Kat6b)* mice. Flow cytometry gating strategies defining haematopoietic cell population using cell surface markers as described in Fig. 4. Each circle represents an individual mouse. Data in (**e**, **g**, **h**) are presented as mean ± s.e.m. Each circle represents an individual mouse. Data were analysed using a two-way ANOVA with Tukey post-hoc correction. Abbreviations as defined in Fig. 4. Flow cytometry gating strategy (**d**) and analysis (**e**) of B cell progenitor populations in the bone marrow. Gating strategy (**f**) and analysis (**g**, **h**) of haematopoietic stem and progenitor population in the bone marrow.

*Kat6a*^*–/–*^*Kat6b*^*+/+*^mice die between E14.5-E18.5 depending on the genetic background^17,18^. Overexpression of *Kat6b* completely rescued this foetal to perinatal lethality. *Kat6a*^*–/–*^*Tg(Kat6b)* mice developed normally in the postnatal period, even compared to *Kat6a* heterozygous (*Kat6a*^*+/–*^) mice, which were notably runted in early life (Fig. 7a, b). Furthermore, *Kat6a*^*–/–*^*Tg(Kat6b)* mice showed normal weight gain over the first 3 weeks of life, compared to reduced weight gain observed in both male and female *Kat6a*^*+/–*^ mice (*p* = 0.001 and 1 × 10^–5^; Fig. 7b). Remarkably, *Kat6a*^*–/–*^ *Tg(Kat6b)* mice of both sexes reached adulthood and were healthy and fertile. Among the offspring of *Kat6a*^*–/–*^*Tg(Kat6b)* x *Kat6a*^*+/–*^*Kat6b*^*+/+*^ matings, *Kat6a*^*–/–*^*Kat6b*^*+/+*^ mice were completely absent (*N* = 0 of 94; *p* < 10^–6^; Fig. 7c). In contrast, *Kat6a*^*–/–*^*Tg(Kat6b)* mice were present at the expected Mendelian ratio at weaning (*N* = 32 of 94; *p* = 1; Fig. 7c). This demonstrates that KAT6B overexpression in the absence of KAT6A not only rescued the effects of the homozygous loss of *Kat6a*, but improved development in early life compared to *Kat6a* heterozygosity.

Given the requirement for KAT6A in the adult haematopoietic system^49,55^, bone marrow stem and progenitor populations were assessed in adult *Kat6a*^*–/–*^*Tg(Kat6b)* mice at 12 weeks of age. All stem and progenitor populations showed similar frequencies as in wild-type control mice (Fig. 7d–h), indicating that haematopoietic development in adulthood is normal in *Kat6a*^*–/–*^*Tg(Kat6b)* animals.

## Discussion

In this study we demonstrate that KAT6B, expressed at 4-fold above endogenous levels in *Kat6a*^*–/–*^ mice, rescues the previously described developmental defects resulting from homozygous loss of KAT6A. Remarkably, *Kat6a*^*–/–*^*Tg(Kat6b)* mice are born, show normal development to adulthood and normal blood cell development in adulthood.

In addition to rescuing the anatomical defects resulting from loss of KAT6A, KAT6B overexpression reverted ~90% of the changes in gene expression caused by loss of KAT6A and re-established global acetylation levels at H3K9 and H3K23 in whole E9.5 embryos and H3K23 in MEFs. Examining locus-specific acetylation we found complete restoration of the normal pattern of H3K23ac down to the level of individual peaks within transcription units. The rescue of genes downregulated in *Kat6a*^*–/–*^*Kat6b*^*+/+*^ mice by KAT6B overexpression is particularly interesting, as KAT6B is not ordinarily required for expression of many KAT6A-dependent genes. In particular, while KAT6A is essential for the normal expression the *Hox*, gene families, KAT6B has no role in regulation of these genes and the axial skeleton develops normally without KAT6B. The capacity of overexpressed KAT6B to carry out the role of promoting their expression in the absence of KAT6A indicates that any target gene specificity of KAT6A arising from the differences in amino acid sequence between KAT6A and KAT6B, and perhaps affecting protein-protein interaction, can be overcome by higher levels of KAT6B.

*In utero* treatment with retinoic acid, an activator of *Hox* gene expression^56,57^, rescues the anterior homeotic transformation in *Kat6a*^*–/–*^ mice, but not other developmental defects^17^. Indeed, *in utero* retinoic acid treatment causes cardiac defects in *Kat6a*^*+/–*^ mice^18^. Conversely, the cardiac septum defect of *Kat6a*^*–/–*^ mice was rescued by overexpressing the *Tbx1* gene^18^. Body segment identity has been restored in *Kat6a*^*–/–*^ mice by additionally deleting *Bmi1*^54^. BMI1 is a polycomb repressor protein that represses *Hox* gene expression and, when deleted, causes a posterior homeotic transformation^58,59^. Combined deletion of *Kat6a* and *Bmi1* genes was found to restore the respective anterior and posterior homeotic transformations observed in single mutant mice^54^, but did not rescue the haematopoietic defects of *Kat6a*^*–/–*^ mice^55^. Collectively, these results suggest that KAT6A does not act as a binary on-off switch, but rather the level of KAT6A activity acts to balance the activity of repressors to generate an appropriate level of gene expression in a cell-type specific context.

Our data suggest that KAT6B overexpression in the absence of KAT6A may result in a more favourable stoichiometry of complex subunits to allow KAT6B to take on the role of KAT6A, compared to endogenous levels of KAT6B or KAT6B overexpression in the presence of KAT6A. In the absence of KAT6A, the limitation of auxiliary complex member availability may permit KAT6B, when overexpressed, to perform its own typical roles, as well as those of KAT6A, without spurious effects resulting from an overabundance of KAT6B. This is evident at the level of global histone acetylation, as acetylation levels at H3K9 and H3K23 in *Kat6a*^*–/–*^*Tg(Kat6b)* E9.5 embryos were similar to wild type controls, while these residues were hyperacetylated in *Kat6a*^*+/+*^*Tg(Kat6b)* samples.

Notwithstanding the profound rescue of the expression levels of genes downregulated in *Kat6a*^*–/–*^*Kat6b*^*+/+*^ embryos at E9.5 when *Kat6b* is overexpressed, some effects of the loss of KAT6A persisted. When only *Tbx* genes were considered (as opposed to transcriptome-wide analyses), *Tbx1* and *15* were still significantly downregulated in *Kat6a*^*–/–*^*Tg(Kat6b)* embryos compared to wild type control embryos. However, *Tbx1* was significantly increased in *Kat6a*^*–/–*^*Tg(Kat6b)* embryos compared to *Kat6a*^*–/–*^*Kat6b*^*+/+*^ samples. This partial rescue was sufficient to rescue the cardiac defects of *Kat6a*^*–/–*^*Kat6b*^*+/+*^ mice, consistent with the rescue of heart defects in *Kat6a*^*–/–*^ mice by transgenic overexpression of *Tbx1*^18^.

In addition to rescuing congenital defects resulting from loss of KAT6A, KAT6B overexpression restored the formation of definitive HSCs capable of reconstituting the haematopoietic system of irradiated recipient mice. Paralleling a previous report that loss of KAT6A reduces CD8 cell surface expression^60^, we found that KAT6B overexpression promoted CD8 T cell formation at the expense of CD4 T cell development in transplant recipient mice, and that this effect of overexpressed KAT6B was modulated by the loss of KAT6A.

Recently, inhibitors have been developed for both CBP/p300^61^ and KAT6A/KAT6B^30^ protein pairs with the view of developing novel cancer therapeutics. Currently, the KAT6A/KAT6B inhibitors are in clinical trials for the treatment of solid cancers^31,32,62^. In the process of developing these inhibitors it has become clear that it would be unlikely that any inhibitor would differentially inhibit one and not the other of each of these protein pairs. It is therefore relevant to determine the functional equivalence of the proteins within pairs. The combined deletion of these proteins only reveals how similar their functions are at endogenous expression levels. In contrast, our data on the rescue of the *Kat6a*^*–/–*^ mice by overexpression of KAT6B indicate that KAT6B can replace KAT6A so completely that it can rescue the 100% lethality, producing healthy and fertile mice that lack KAT6A. These results suggest that simultaneous inhibition of both KAT6A and KAT6B function is likely to be beneficial in treating cancers dependent on KAT6 activity.

## Methods

### Mice

All animal experiments were conducted with approval of the WEHI Animal Ethics Committee and according to the Australian code for the care and use of animals for scientific purposes. Mice were kept in a 14 h light/10 h dark cycle. Noon of the day the vaginal copulation plug was first observed was defined as embryonic day 0.5 (E0.5).

### Mouse alleles

The *Kat6a* null allele used in this study lacked exons 5 to 9 and has been described previously^17^. KAT6B overexpression transgenic mice were generated by microinjected into mouse pronuclei using bacterial artificial chromosome (BAC) *pBACe3.6* clone *RP23-360F23* to produce a germline founder. BAC clone *RP23-360F23* includes the complete wild-type *Kat6b* gene, as well as 21 kb 5’ and 42 kb 3’ containing regulatory sequences. Seven copies of the *BAC* inserted into the mouse genome to result in an ~4-fold increase in *Kat6b* expression as described previously^23^. Mice were maintained on a *FVB* x *BALB/c* hybrid background as *Kat6b* overexpressing mice were not viable on inbred backgrounds. Mice were genotyped by genomic 3-way PCR for the *Kat6a* allele and by simple PCR to detect the *SacB* gene in the backbone of *pBACe3.6* clone *RP23-360F23* using the primers listed in Supplementary Table 1.

### Primary mouse embryonic fibroblast (MEF)

MEFs were derived from E14.5 foetuses and grown in Dulbecco’s modified Eagle medium (Gibco, 11995) supplemented with 100 U/ml penicillin/streptomycin (Gibco, 15140122) and 10% foetal calf serum. Cells were cultured in at 37 °C, 5% CO_2_ and 3% O_2_. Cell counts were determined at each passage using a Countess^TM^ cell counter (ThermoFisher).

### Acid histone extraction

MEFs were washed in DPBS (Gibco, 14190144) containing 0.5 mM sodium butyrate (Sigma, B5887) and cOmplete™ EDTA-free protease inhibitor cocktail (Roche, 11873580001), scraped using a cell scraper (Fisher Scientific, 08-100-241) and collected by centrifugation (200 × *g*, 5 min). Whole E9.5 embryos were dissected and photographed under a dissecting microscope (Zeiss) and placed into a 1.5 ml Eppendorf tube containing 100 μl DPBS with 0.5 mM sodium butyrate (Sigma, B5887) and cOmplete™ EDTA-free protease inhibitor cocktail (Roche, 11873580001). Samples were lysed in Histone acid lysis buffer (10 mM HEPES pH 7.9, 1.5 mM MgCl_2_, 10 mM KCl and 0.5 mM DTT) for 30 min at 4 °C on a roller, collected by centrifugation (10,000 × *g*, 10 min) and resuspended in 0.2 M H_2_SO_4_. Samples were incubated on ice for 1–2 h before being dialysed in dialysis tubing (Spectrum^TM^ Spectra/Por Dialysis Membrane Tubing; molecular weight cut-off 20 kDa; Fisher Scientific, 08-607-067) against 0.1 M acetic acid (Sigma, A6283) for 1 h at 4 °C and MQ-H_2_O overnight at 4 °C. Protein concentrations were determined using a bicinchoninic acid (BCA) assay (ThermoFisher, 23225).

### Western immunoblotting

Acid extracted histones were run on 4–12% Bis-Tris gels (ThermoFisher, NP0322) and transferred onto nitrocellulose membranes (Licor, 926-31090). Membranes were blocked for 1 h at room temperature (RT) on a roller in blocking buffer (Intercept® (PBS); Licor, 927-70001) and probed with antibodies again H3K9ac (Epicypher, 13-0033; dilution 1:5000), H3K14ac (Abcam, ab52946; 1:1000) or H3K23ac (Millipore, 07-355; 1:5000) and pan H3 (Abcam, 10799; 1:5000) overnight at 4 °C. The following morning membranes were washed in PBS + 0.1% Tween-20 (Sigma, P1379) and incubated with goat anti-mouse IgG secondary (IRDye® 800 CW; LI-COR Biosciences 926-32210; 1:10,000) and goat anti-rabbit IgG (IRDye®; LI-COR Biosciences, 926-68071; 1:10,000) secondary antibodies for 1 h at RT on a roller. Samples were imaged and analysed using an automated western blot imager software (Odyssey Imager; LI-COR Biosciences).

### Histology

E18.5 hearts were dissected, washed in PBS and fixed overnight in 10% neutral buffered formalin. Hearts were embedded in agarose to control orientation, then dehydrated and infused with paraffin and embedded for histological sectioning and haematoxylin and eosin (H&E) staining.

### Skeletal preparations

Skin and internal organs were removed from E18.5 pups under a dissecting microscope (Zeiss). Pups were fixed in 4% PFA (Sigma, 158127) overnight at RT on a roller, briefly rinsed in 95% EtOH and stained overnight at RT in a solution containing 5 ml 0.4% Alcian Blue 8 GX (w/v), 5 ml glacial acetic acid, 70 ml 95% EtOH, 20 ml MQ-H_2_O and 100 µl 0.5% Alizarin red (w/v). Samples were washed in MQ-H_2_O. Soft tissues were dissolved in 2% (w/v) KOH (Sigma, 221473) in H_2_O for 24 h at RT on a roller. Following digestion, skeletal preparations were washed in 0.25% (w/v) KOH in H_2_O for 30 min at RT on a roller, followed by ascending concentrations of glycerol (20%, 33%, 50%) in 0.25% (w/v) KOH in H_2_O, for 1 h, 1 h and overnight, respectively at RT on a roller. Prepared skeletons were stored in 50% (w/v) glycerol (Sigma, G5516) in ddH_2_O.

### Whole mount in situ hybridization

Embryos used for whole-mount in situ hybridisation were fixed in 4% paraformaldehyde overnight then dehydrated through a methanol series and stored at −20 °C. After genotyping selected embryos were rehydrated through a methanol series, washed in phosphate buffer saline/Tween20, then bleached in hydrogen peroxide, washed in phosphate buffer saline/Tween20, then treated with proteinase K which was stopped by the addition of 1 M glycine. Embryos were then prehybridised a solution of 50% Formamide/5x SSC ph4.5/ 1% SDS/ 50 µg/ml yeast RNA/ 50 µg/ml heparin for 1 h. Embryos were then transferred to a fresh aliquot of the hybridisation buffer containing in vitro transcribed (Roche 11175025910), digoxigenin-labelled cRNA of the *Hox* gene under investigation and incubated at 55 °C overnight. Then embryos were washed extensively, treated with RnaseA, blocking reagent (Roche 10057177103) and foetal bovine serum, incubated with alkaline phosphatase- labelled anti-digoxigenin antibody (Roche 11214667001) overnight, washed extensively, followed by alkaline phosphatase reaction with NBT-BCIP (Roche 11681451001) for colour development. Finally, embryos were washed and then cleared in glycerol^63^. Sense and antisense probes for *Hoxa3*, *Hoxa5* and *Hoxc5* have been previously described^17,54^.

### Foetal liver transplantation

E14.5 foetal livers were dissected and passed through a 40 μm cell sieve (Corning, 431750). 1 × 10^6^ cells, as determined using an automated haematology analyser (Advia 2120i, Siemens Healthineers), were injected into the tail vein of 3x irradiated recipients (2 × 550 rad, 3 h apart)^49^.

### Flow cytometry

Erythrocytes were lysed by washing samples 2 × 10 ml in a hypotonic solution (150 mM NH_4_Cl, 0.1 mM EDTA, 12 mM NaHCO_3_, pH 7.2). Cells were resuspended in a FACS buffer (150 nM NaCl, 3.7 mM KCl, 2.5 mM CaCl_2_2H_2_O, 1.2 mM MgSO4•7H_2_O, 0.8 mM K_2_HPO_4_, 1.2 mM KH_2_PO_4_, 11.5 mM HEPES, pH 7.4) supplemented with 2% foetal calf serum and stained with conjugated antibodies (Supplementary Table 2) for 1 h on ice. Samples were washed in 3–4 ml FACS buffer and analysed on a flow cytometer (BD LSRFortessa^TM^ X-20, BD) at <7500 events/sec. Data were analysed using flow cytometry analysis software (FlowJo version 10.7, Tree Star Inc.). Cell surface markers used to identify individual cell types are shown in Supplementary Table 3.

### RNA isolation and sequencing

Total RNA from whole E9.5 embryos was extracted using an RNA extraction kit (Qiagen RNeasy mini kit; Qiagen, 74104), according to the manufacturer’s instructions and including the optional DNaseI digestion step. RNA quality and quantity were assessed on an automated analyser (Tapestation 4200; Agilent, G2991BA), and 500 ng RNA used to generate libraries using a library construction kit (TruSeq RNA prep kit v2; Illumina, RS-122-2002), according to the manufacturer’s instructions. Samples were run on a sequencing machine (NextSeq 2000; Illumina) to give 66 bp paired end reads.

### RNA sequencing analysis

Reads were aligned to the Mus musculus (mm39) genome using Rsubread^64^. Differential expression (DE) analyses were performed using the edgeR and limma software packages^65^. Library sizes were normalised using the trimmed mean of *M*-values (TMM) method^66^ and the surrogate variable approach^67^ was used to adjust for unwanted variation in the data. The false discovery rate (FDR) was controlled below 0.05. R version 4.2.2 was used for all analyses.

### Cut&Tag detection of H3K23 acetylation

CUT&Tag sequencing was performed on 50,000 MEFs combined with 50,000 *Drosophila melanogaster* S2 cells, as described in Kaya-Okur et al.^68^, with minor modifications as described in ref. ^69^. All buffers were prepared fresh with complete EDTA-free protease inhibitors (Roche, 11873580001) and 0.5 mM sodium butyrate (Sigma, B5887). Per sample, 10 µl concanavalin-A beads (Bangs Laboratories, BP531) were washed twice in 10 volumes binding buffer (20 mM HEPES pH 7.5 (Sigma, 83264), 10 mM KCl (Sigma, 60142), 1 mM CaCl_2_ (Sigma, 21115) and 1 mM MnCl_2_ (Sigma, M1787) and resuspended in 10 µl binding buffer. Combined MEFs and S2 *D. melanogaster* cells were washed twice in 1 ml wash buffer (20 mM HEPES pH 7.5, 150 mM NaCl (Sigma, 71386), 0.5 mM spermidine (Sigma, S0266) and resuspended in 90 µl wash buffer. Concanavalin-A beads and samples were combined and incubated (RT, 10 min), immobilised using a magnetic rack (ThermoFisher, MR02) and beads resuspended in 100 µl ice-cold antibody buffer (wash buffer supplemented with 0.05 % (w/v) digitonin (Merck, 300410), 2 mM EDTA (Invitrogen, 15575020) and 0.1 % (w/v) BSA (Sigma, A8577) containing 1:100 H3K23ac (Millipore, 07-355) primary antibody. Samples were incubated overnight at 4 °C. The following morning, beads were resuspended in 100 µl wash buffer supplemented with 0.05% (w/v) digitonin containing 1:100 secondary antibody (Guinea pig anti-rabbit IgG, Antibodies Online, ABIN101961), incubated (RT, 1 h), washed thrice and resuspended in 100 µl ice-cold dig-300 buffer (20 mM HEPES pH 7.5, 300 mM NaCl, 0.5 mM spermidine and 0.01 % (w/v) digitonin), supplemented with 1 2.5 µl pAG-Tn5 (EpiCypher, 15-1017). Samples were incubated (RT, 1 h), washed thrice, resuspended in 100 µl tagmentation buffer (wash buffer supplemented with 0.01 mM MgCl2 (Sigma, 63069) and incubated at 37 °C for 1 h. Tagmentation was stopped using 3.34 µl 0.5 M EDTA, 1 µl 10% (w/v) SDS (Sigma, 71736), and 0.83 µl 20 mg/ml thermolabile proteinase K (NEB, P8111S), incubating at 37 °C for 1 h and 800 rpm, followed by heat inactivation at 55 °C for 10 min. DNA was extracted using Ampure XP beads (Beckman, A63880), eluting in 25 µl 10 mM Tris-HCl pH = 8.0 (Invitrogen, 15568025), 1 mM EDTA and 25 µg/ml RNAse A (ThermoFisher, EN0531) at 37 °C for 10 min. 10 µl sample DNA elutes were used to generate sequencing libraries, which were amplified using PCR for 13 cycles. PCR products were cleaned up using 30 µl Ampure XP beads and eluted in 25 µl 10 mM Tris-HCl pH 8.0. Cleaned libraries were analysed using High Sensitivity D1000 gels (Agilent, 5067- 5584) on an Agilent 2200 tape station. Libraries were sequenced using an Illumina NextSeq2000.

### Analysis of CUT&Tag data

All samples were composed of *Mus musculus* (test) and *Drosophila melanogaster* RNA (spike-in for normalisation^70^). Furthermore, the transgenic BAC backbone samples contained the *sacB* gene. For alignment an index was built using Rsubread v2.18.0^71^ containing the mouse genome (mm39), the *Drosophila* genome (R655) and the *sacB* gene sequence. All samples were aligned to this combined genome index using Rsubread’s align function reporting uniquely mapped reads only. All PCR duplicate reads were then marked using sambamba v0.6.6. The data was then summarised at both the species and mouse gene level. Data around mouse genes was summarised at −1 kbp upstream of the TSS to the TSS, transcription end site (TES) to +1kbp downstream of the TES, and the gene body (TSS to TES). Fragment counts were produced using Rsubread’s featureCounts function. Fragments were counted if they were not a PCR duplicate and non-chimeric. Mouse genes were identified using RefSeq annotation to the mm39 genome. The analysis of each region was restricted to protein coding genes with official gene symbols. Riken, Gm (predicted), and pseudogenes were also removed. To avoid sex-based biases, the Xist gene and Y-chromosome were removed. Analysis of each region was then conducted independently. Differential analyses were conducted using the limma^65^ and edgeR^66^ software packages, versions 3.60.4 and 4.2.1 respectively.

For each analysis, lowly abundant regions were filtered using edgeR’s filterByExpr function with default parameters. The samples were then normalised to Drosophila melanogaster content using the following method:

1. Calculate total *drosophila* content for each sample.
2. Divide the above by the total filtered mouse counts for that sample.
3. Divide the resulting numbers by the product of all values calculated in step 2 to the power of (1/number of samples).

Differential analyses between genotypes were then conducted as follows.

TSS region: the data was transformed to log_2_-counts per million (CPM) and sample weights were calculated using limma’s arrayWeights function using the genebygene method^72^. Linear models were fit to each region and robust empirical bayes moderated t-statistics with a trended prior variance were then applied to identify differential regions (robust limma-trend pipeline with sample weights)^73^.

Gene body region: the data was transformed to log_2_CPM with associated precision weights using voom. Linear models were then fit to each genomic region, differences between groups were assessed using robust empirical bayes moderated t-statistics (robust limma-voom pipeline)^73^.

TES region: Limma’s voomWithQualityWeights function was applied to simultaneously transform the data to log_2_CPM with associated precision weights and estimate sample level weights^74^. Linear models were then fit to each genomic region, differences between groups were assessed using robust empirical bayes moderated t-statistics (robust limma-voomWithQualityWeights pipeline).

For each analysis the false discovery rate (FDR) was controlled below 5% using the Benjamini and Hochberg method.

### Statistics

The statistical analysis methods for the RNA-sequencing and CUT&Tag data are provided under the *RNA-sequencing analysis* and *Analysis of CUT&Tag data* section. Other data are presented as mean ± s.e.m. In all graphs circles represent individual mice or the average of transplant recipients that received cells from a single foetal liver donor. Statistical analyses were performed in Prism Graphpad Version 8.3.1 for Mac (GraphPad Software) and R version 4.2.2. Statistical tests employed and the number of biological replicates are stated in the figure legends.

### Reporting summary

Further information on research design is available in the Nature Portfolio Reporting Summary linked to this article.

## Supplementary information

Supplementary Information

Description of Additional Supplementary Files

Supplementary Data 1

Supplementary Data 2

Supplementary Data 3

Supplementary Data 4

Supplementary Data 5

Supplementary Data 6

Supplementary Data 7

Supplementary Data 8

Supplementary Data 9

Supplementary Data 10

Supplementary Data 11

Supplementary Data 12

Reporting Summary

## Source data

Source Data

Transparent Peer Review file

## Data Availability

All RNA sequencing and CUT&Tag data has been deposited in the NCBI GEO database under accession numbers: GSE287243 and GSE287244 [https://www.ncbi.nlm.nih.gov/geo/query/acc.cgi?acc=GSE287244]. Source data are provided with this paper.
