# Supplementary material for: KAT6B overexpression rescues embryonic lethality in homozygous null KAT6A mice restoring vitality and normal lifespan
Source: Nat Commun. 2025 Feb 25;16:1958. doi: 10.1038/s41467-025-57155-4 (PMC11861323; doi:10.1038/s41467-025-57155-4)
Supplement: Supplementary file 1 — Supplementary Information [file 41467_2025_57155_MOESM1_ESM.pdf]

Supplementary Data and Supplementary information

KAT6B overexpression rescues embryonic lethality in homozygous null KAT6A mice restoring vitality and normal lifespan

Maria. I. Bergamasco[1,2], Yuqing Yang[1,2], Alexandra L. Garnham[1,2], Bilal N. Sheikh[3,4], Gordon K. Smyth[1,5], Anne. K. Voss[1,2,*,#] and Tim Thomas[1,2,*,#]

[1] The Walter and Eliza Hall Institute of Medical Research, Parkville, Victoria 3052, Australia

[2] Department of Medical Biology, The University of Melbourne, Parkville, Victoria 3052, Australia

[3]Helmholtz Institute for Metabolic, Obesity and Vascular Research (HI-MAG) of the Helmholtz Center Munich, Leipzig, Germany

[4]Medical Faculty, University of Leipzig, Leipzig, Germany

[5]School of Mathematics and Statistics, University of Melbourne, Parkville, Victoria 3010, Australia.

*These authors contributed equally and share senior authorship

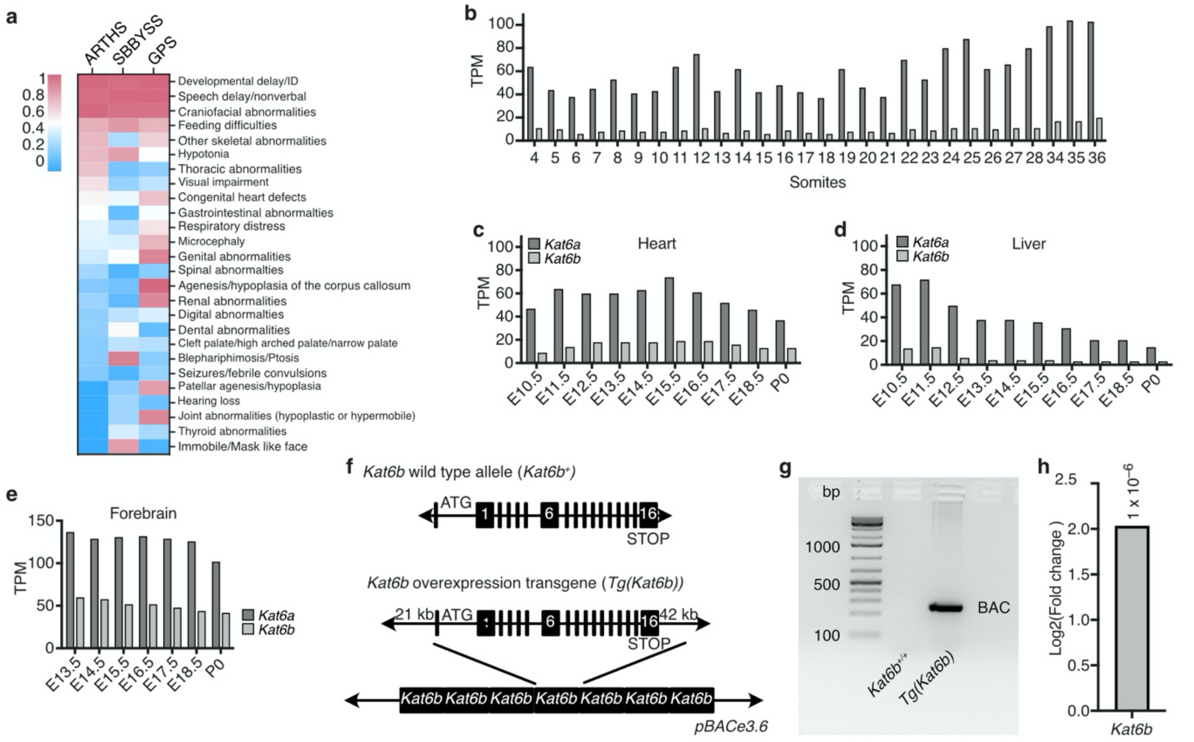

Supplementary Figure 1: Comparison of human disorders caused by heterozygous mutations in the *KAT6A* or *KAT6B* gene, *Kat6a* and *Kat6b* gene expression, and generation of mice overexpressing *Kat6b*

(a) Heat map of the proportion (0-1) of commonly described traits in Arboleda-Tham syndrome (ARTHS), Say-Barber-Biesecker-Young-Simpson (SBBYSS) and Genitopatellar syndrome (GPS) patients. Related data shown in Supplementary Table 1.

(b) Comparison of *Kat6a* and *Kat6b* mRNA levels (TPM counts) in early mouse embryos, staged by somite count. Based on data collected from Expression Atlas[1]

(c) Comparison of *Kat6a* and *Kat6b* mRNA levels (TPM counts) during pre-natal heart development, based on data collected from Gene Expression Database (GXD), Mouse Genome Informatics website, based on sequencing data from[2]

(d) Comparison of *Kat6a* and *Kat6b* mRNA levels (TPM counts) during pre-natal liver development collected from Gene Expression Database (GXD), Mouse Genome Informatics website, based on sequencing data from[2]

(e) Comparison of *Kat6a* and *Kat6b* mRNA levels (TPM counts) in foetal forebrain development collected from Gene Expression Database (GXD), Mouse Genome Informatics website, based on sequencing data from[2]. Note that the relative levels of *Kat6b* compared to *Kat6a* in the brain are higher than other tissues where *Kat6a* dominates.

(f) Schematic drawing of the wild type *Kat6b* allele and the *pBACe3.6* clone *RP23-360F23* that contains the mouse *Kat6b* gene, as well as 21 kb 5' and 42 kb 3' of the coding exons, inserted as seven copies into the mouse genome to form the *Tg(Kat6b)* transgene.

(g) Genotyping PCR to detect SacB in the BAC backbone of the *Tg(Kat6b)* transgene.

(h) Log_2 fold change in *Kat6b* expression in *Tg(Kat6b)* E9.5 embryos compared to wild type controls. FDR = 1 x 10^{-6}. N = 4 embryos per genotype.

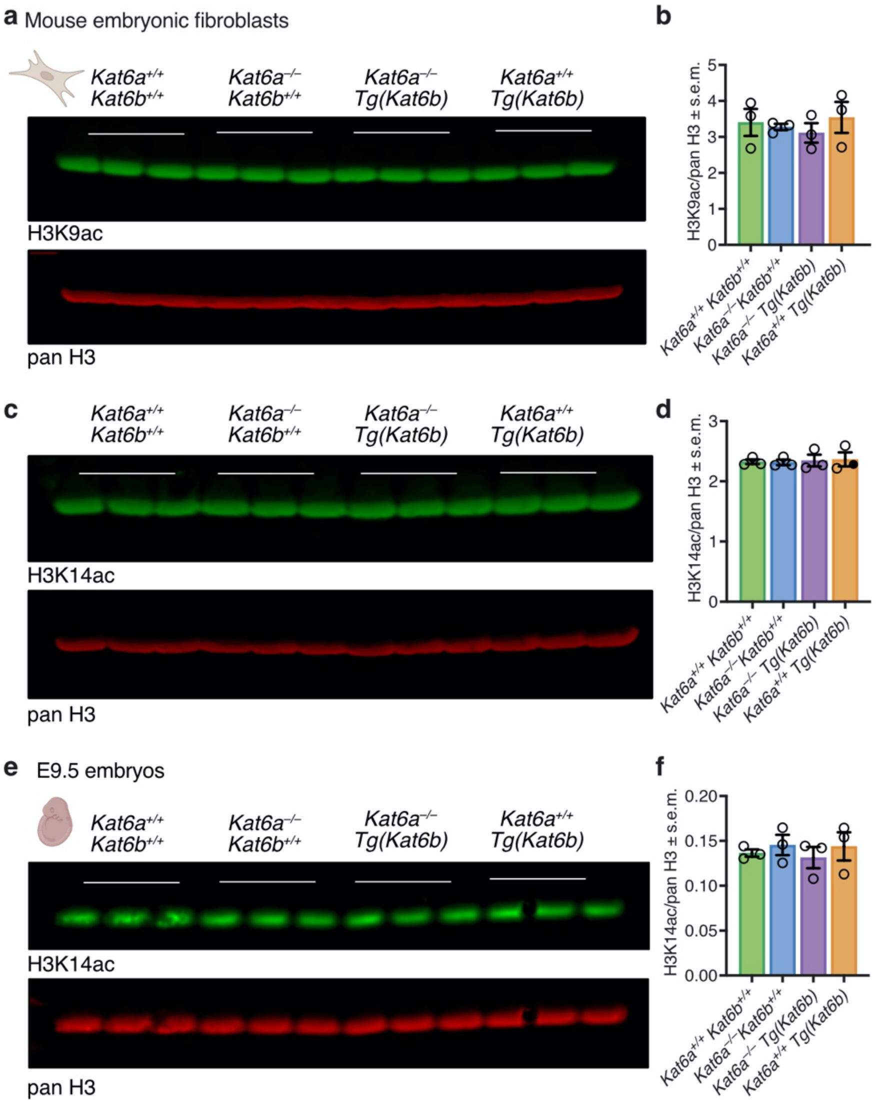

Supplementary Figure 2: KAT6A and KAT6B do not affect H3K9 or H3K14 acetylation in MEFs or H3K14 acetylation in E9.5 embryos

(a-d) Western immunoblot of histone H3 lysine 9 (H3K9; a, b) and H3K14 (c, d) acetylation, with pan-H3 as a loading control, in mouse embryonic fibroblasts (MEFs). Densitometry (b, d) of the immunoblots in (a, c).

(e,f) Western immunoblot of H3K14 acetylation with pan-H3 as a loading control (e) in whole E9.5 embryos, with densitometry in (f).

N = MEFs isolated from 3 foetuses per genotype (a-d) or 3 embryos per genotype (e-f). 500 ng (a) or 2 µg (c, e) acid extracted histone protein loaded per sample. Each lane on the Western blots represents a MEF culture derived from an individual E14.5 embryo (a-d) or individual

E9.5 embryo (e,f). Data are presented as mean ± s.e.m. and were analysed using a one-way ANOVA with Dunnett post hoc correction (b, d, f).

Created in BioRender. Bergamasco, M. (2025) https://BioRender.com/m13f247

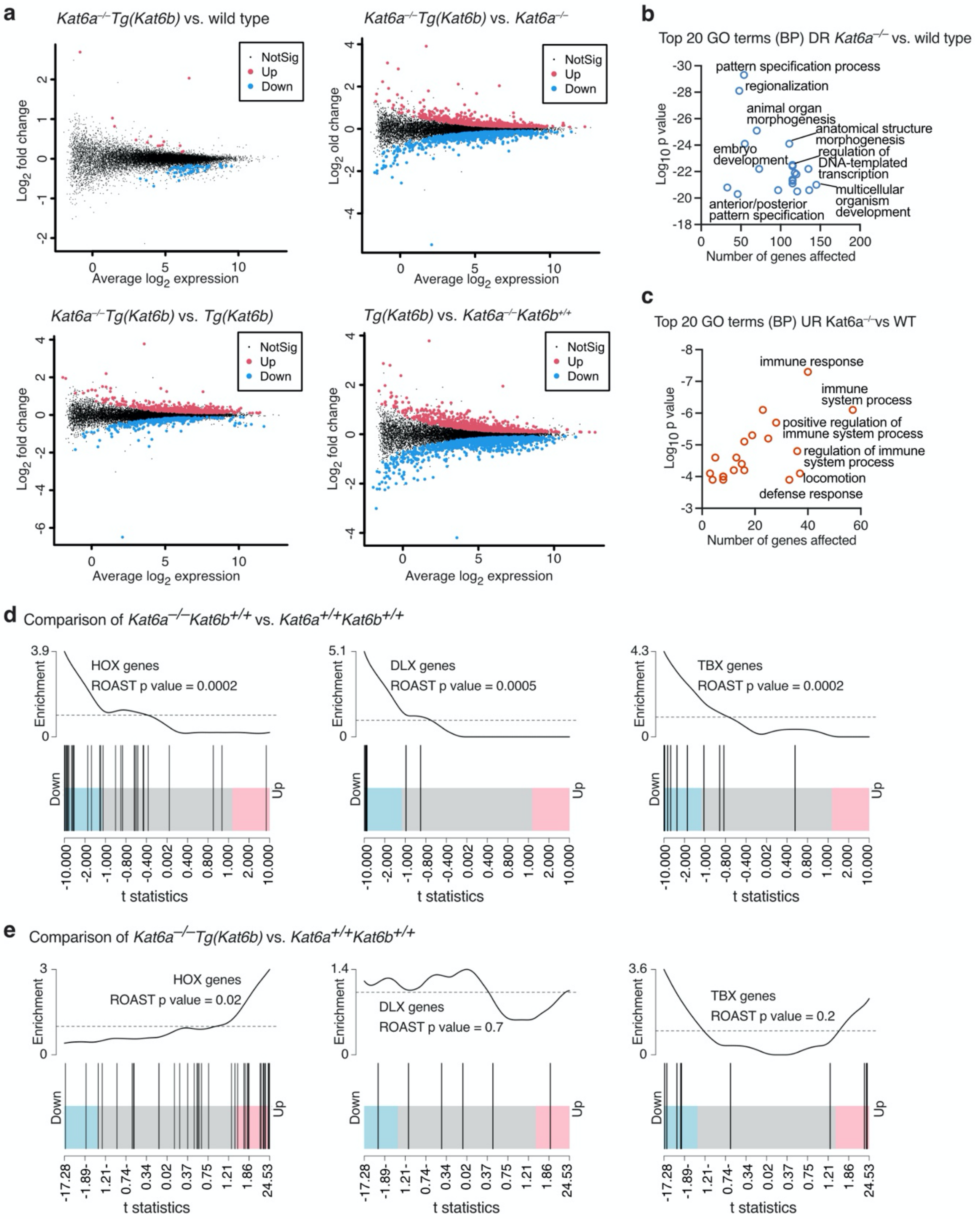

Supplementary Figure 3: Deletion of the *Kat6a* gene results in reduced expression of development control genes and increased expression of less specific genes in E9.5 embryos

(a-c) RNA-sequencing data of *Kat6a*$^{+/+}$*Kat6b*$^{+/+}$, *Kat6a*$^{-/-}$*Kat6b*$^{+/+}$, *Kat6a*$^{+/+}$*Tg(Kat6b)* and *Kat6a*$^{-/-}$*Tg(Kat6b)* E9.5 embryos. N = 4 embryos per genotype. Data were analysed as

described in the methods section. A false discovery (FDR) < 0.05 was used as the cut off for significance.

(a) M (log_2 ratio) and A (mean average, log_2 CPM) plots showing genes significantly (FDR < 0.05) upregulated (UR) in red, genes significantly downregulated (DR) in blue and unchanged genes in grey.

(b,c) Top 20 Gene Ontology (GO) terms (BP) enriched in genes downregulated (b) or upregulated (c) in *Kat6a*$^{-/-}$*Kat6b*$^{+/+}$ vs. *Kat6a*$^{+/+}$*Kat6b*$^{+/+}$ E9.5 embryos. GO terms enriched with $p < 10^{-6}$ are shown.

(d,e) Barcode plots showing the distribution of HOX, DLX and TBX genes as indicated among genes expressed in *Kat6a*$^{-/-}$ *Kat6b*$^{+/+}$ vs. *Kat6a*$^{+/+}$*Kat6b*$^{+/+}$ embryos (d) and *Kat6a*$^{-/-}$ *Tg(Kat6b)* vs. *Kat6a*$^{+/+}$*Kat6b*$^{+/+}$ embryos (e) ordered from down-regulated, left, to up-regulated, right, with the t-statistic on the x axis. The vertical lines represent HOX, DLX and TBX genes. The worms indicate the enrichment of these gene families either among the downregulated genes (all in d) or among the upregulated genes (HOX genes in e) or the lack of enrichment, namely relatively even distribution among all expressed genes (e.g., DLX genes in e). The p values from the rotation gene set tests (ROAST) enrichment analysis are displayed in the plots.

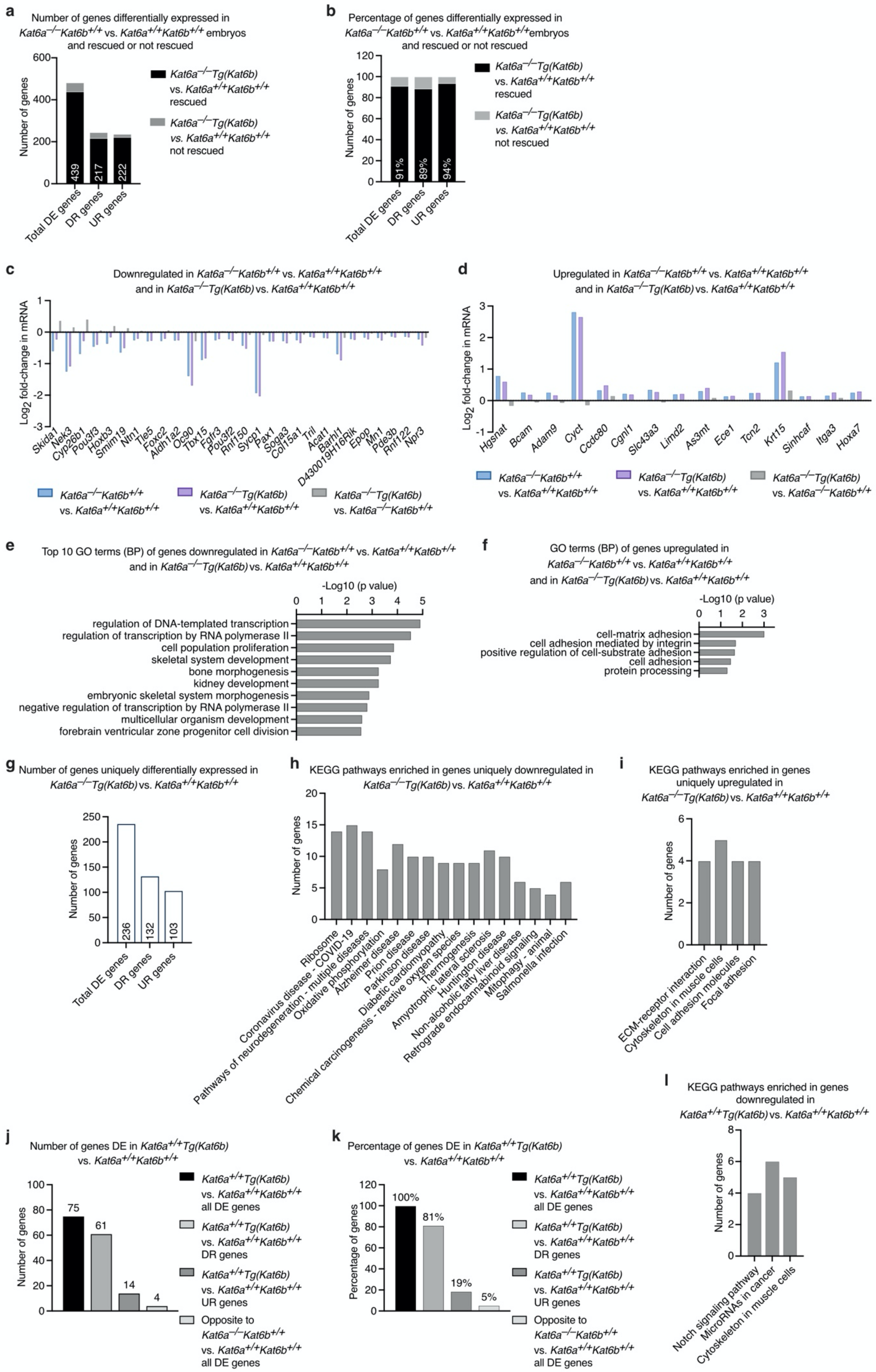
a
Number of genes differentially expressed in Kat6a−/−Kat6b+/+ vs. Kat6a+/+Kat6b+/+ embryos and rescued or not rescued
Number of genes
Kat6a−/−Tg(Kat6b) vs. Kat6a+/+Kat6b+/+ rescued
Kat6a−/−Tg(Kat6b) vs. Kat6a+/+Kat6b+/+ not rescued
439
217
222
Total DE genes
DR genes
UR genes
b
Percentage of genes differentially expressed in Kat6a−/−Kat6b+/+ vs. Kat6a+/+Kat6b+/+embryos and rescued or not rescued
Number of genes
Kat6a−/−Tg(Kat6b) vs. Kat6a+/+Kat6b+/+ rescued
Kat6a−/−Tg(Kat6b) vs. Kat6a+/+Kat6b+/+ not rescued
91%
89%
94%
Total DE genes
DR genes
UR genes
c
Downregulated in Kat6a−/−Kat6b+/+ vs. Kat6a+/+Kat6b+/+ and in Kat6a−/−Tg(Kat6b) vs. Kat6a+/+Kat6b+/+
Log2 fold-change in mRNA
Skida1 Nek3 Cyp26b1 Pou3f3 Hoxb3 Smim19 Ntn1 Tle5 Foxc2 Aldh1a2 Oc90 Tbx15 Fgfr3 Pou3f2 Rnf150 Sycp1 Pax1 Soga3 Col15a1 Tril Acat1 Barhl1 D430019H16Rik Epop Mn1 Pde3b Rnf122 Npr3
Kat6a−/−Kat6b+/+ vs. Kat6a+/+Kat6b+/+
Kat6a−/−Tg(Kat6b) vs. Kat6a+/+Kat6b+/+
Kat6a−/−Tg(Kat6b) vs. Kat6a−/−Kat6b+/+
d
Upregulated in Kat6a−/−Kat6b+/+ vs. Kat6a+/+Kat6b+/+ and in Kat6a−/−Tg(Kat6b) vs. Kat6a+/+Kat6b+/+
Log2 fold-change in mRNA
Hgsnat Bcam Adam9 Cyct Ccdc80 Cgnl1 Slc43a3 Limd2 As3mt Ece1 Tcn2 Krt15 Sinhcaf Itga3 Hoxa7
Kat6a−/−Kat6b+/+ vs. Kat6a+/+Kat6b+/+
Kat6a−/−Tg(Kat6b) vs. Kat6a+/+Kat6b+/+
Kat6a−/−Tg(Kat6b) vs. Kat6a−/−Kat6b+/+
e
Top 10 GO terms (BP) of genes downregulated in Kat6a−/−Kat6b+/+ vs. Kat6a+/+Kat6b+/+ and in Kat6a−/−Tg(Kat6b) vs. Kat6a+/+Kat6b+/+
-Log10 (p value)
regulation of DNA-templated transcription
regulation of transcription by RNA polymerase II
cell population proliferation
skeletal system development
bone morphogenesis
kidney development
embryonic skeletal system morphogenesis
negative regulation of transcription by RNA polymerase II
multicellular organism development
forebrain ventricular zone progenitor cell division
f
GO terms (BP) of genes upregulated in Kat6a−/−Kat6b+/+ vs. Kat6a+/+Kat6b+/+ and in Kat6a−/−Tg(Kat6b) vs. Kat6a+/+Kat6b+/+
-Log10 (p value)
cell-matrix adhesion
cell adhesion mediated by integrin
positive regulation of cell-substrate adhesion
cell adhesion
protein processing
g
Number of genes uniquely differentially expressed in Kat6a−/−Tg(Kat6b) vs. Kat6a+/+Kat6b+/+
Number of genes
236
132
103
Total DE genes
DR genes
UR genes
h
KEGG pathways enriched in genes uniquely downregulated in Kat6a−/−Tg(Kat6b) vs. Kat6a+/+Kat6b+/+
Number of genes
Ribosome
Coronavirus disease - COVID-19
Pathways of neurodegeneration - multiple diseases
Oxidative phosphorylation
Alzheimer disease
Prion disease
Parkinson disease
Diabetic cardiomyopathy
Chemical carcinogenesis - reactive oxygen species
Thermogenesis
Amyotrophic lateral sclerosis
Huntington disease
Non-alcoholic fatty liver disease
Retrograde endocannabinoid signaling
Mitophagy - animal
Salmonella infection
i
KEGG pathways enriched in genes uniquely upregulated in Kat6a−/−Tg(Kat6b) vs. Kat6a+/+Kat6b+/+
Number of genes
ECM-receptor interaction
Cytoskeleton in muscle cells
Cell adhesion molecules
Focal adhesion
j
Number of genes DE in Kat6a+/+Tg(Kat6b) vs. Kat6a+/+Kat6b+/+
Number of genes
75
61
14
4
Kat6a+/+Tg(Kat6b) vs. Kat6a+/+Kat6b+/+ all DE genes
Kat6a+/+Tg(Kat6b) vs. Kat6a+/+Kat6b+/+ DR genes
Kat6a+/+Tg(Kat6b) vs. Kat6a+/+Kat6b+/+ UR genes
Opposite to Kat6a−/−Kat6b+/+ vs. Kat6a+/+Kat6b+/+ all DE genes
k
Percentage of genes DE in Kat6a+/+Tg(Kat6b) vs. Kat6a+/+Kat6b+/+
Percentage of genes
100%
81%
19%
5%
Kat6a+/+Tg(Kat6b) vs. Kat6a+/+Kat6b+/+ all DE genes
Kat6a+/+Tg(Kat6b) vs. Kat6a+/+Kat6b+/+ DR genes
Kat6a+/+Tg(Kat6b) vs. Kat6a+/+Kat6b+/+ UR genes
Opposite to Kat6a−/−Kat6b+/+ vs. Kat6a+/+Kat6b+/+ all DE genes
l
KEGG pathways enriched in genes downregulated in Kat6a+/+Tg(Kat6b) vs. Kat6a+/+Kat6b+/+
Number of genes
Notch signaling pathway
MicroRNAs in cancer
Cytoskeleton in muscle cells

Supplementary Figure 4. Characterisation of gene expression not rescued by overexpression of *Kat6b* in *Kat6a-/-* embryos and genes differentially expressed in *Tg(Kat6b)* compared to wild type controls.

(a-l) RNA-sequencing data of *Kat6a*$^{+/+}$*Kat6b*$^{+/+}$, *Kat6a*$^{-/-}$*Kat6b*$^{+/+}$, *Kat6a*$^{+/+}$*Tg(Kat6b)* and *Kat6a*$^{-/-}$*Tg(Kat6b)* E9.5 embryos. N = 4 embryos per genotype. Data were analysed as described in the methods section. A false discovery (FDR) < 0.05 was used as the cut off for significance.

(a,b) The number (a) and percentage (b) of genes differentially expressed in *Kat6a*$^{-/-}$*Kat6b*$^{+/+}$ vs. *Kat6a*$^{+/+}$*Kat6b*$^{+/+}$ embryos and rescued vs. not rescued by overexpression of *Kat6b* in the *Kat6a*$^{-/-}$*Tg(Kat6b)* vs. *Kat6a*$^{+/+}$*Kat6b*$^{+/+}$ embryos. The number (a) and percentage (b) of the rescued genes are indicated within the black bars.

(c) Downregulated genes in *Kat6a*$^{-/-}$ compared to wild type together with downregulated genes in *Kat6a*$^{-/-}$*Tg(Kat6b)* compared to wild type controls.

(d) Upregulated genes in *Kat6a*$^{-/-}$ compared to wild type together with upregulated genes in *Kat6a*$^{-/-}$*Tg(Kat6b)* compared to wild type controls.

(e,f) GO terms associated with downregulated genes (e) and with upregulated genes (f) in *Kat6a*$^{-/-}$ compared to wild type that are not rescued in *Kat6a*$^{-/-}$*Tg(Kat6b)* compared to wild type controls. Note that these analyses are limited by the small number of genes that are not rescued.

(g) Genes uniquely differentially expressed in *Kat6a*$^{-/-}$*Tg(Kat6b)* compared to wild type controls. The number of genes uniquely differentially expressed are indicated within the white bars.

(h) KEGG pathways enriched with genes uniquely downregulated in *Kat6a*$^{-/-}$*Tg(Kat6b)* compared to wild type controls.

(i) KEGG pathways enriched with genes uniquely upregulated in *Kat6a*$^{-/-}$*Tg(Kat6b)* compared to wild type controls. Note that only 3 KEGG pathways are enriched and this driven by less than 10% of the DE genes.

(j,k) The number (j) and percentage (k) of genes differentially expressed in *Kat6a*$^{+/+}$*Tg(Kat6b)* vs. *Kat6a*$^{+/+}$*Kat6b*$^{+/+}$ embryos. The number (j) and percentage (k) of genes are indicated above the bars.

(l) KEGG pathways enriched with genes downregulated in *Tg(Kat6b)* compared to wild type controls.

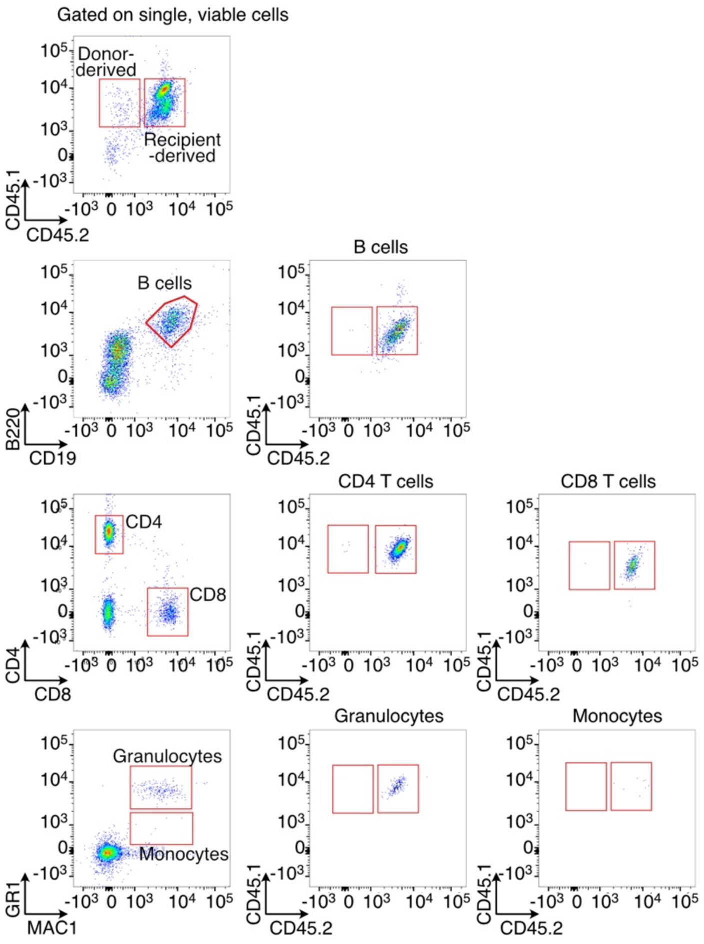

Supplementary Figure 5: *Kat6a*$^{-/-}$ E14.5 foetal liver cells fail to reconstitute irradiated recipients.

Representative flow cytometry profiles of a recipient of *Kat6a*$^{-/-}$*Kat6b*$^{+/+}$ foetal liver cells 3 weeks after transplantation. Foetal liver donor (CD45.1^{+}) cells vs. recipient (CD45.1/2^{+}) are shown for the total live cell population and each peripheral blood cell population analysed. Note the absence of foetal liver donor (CD45.1^{+})-derived peripheral white blood cells.

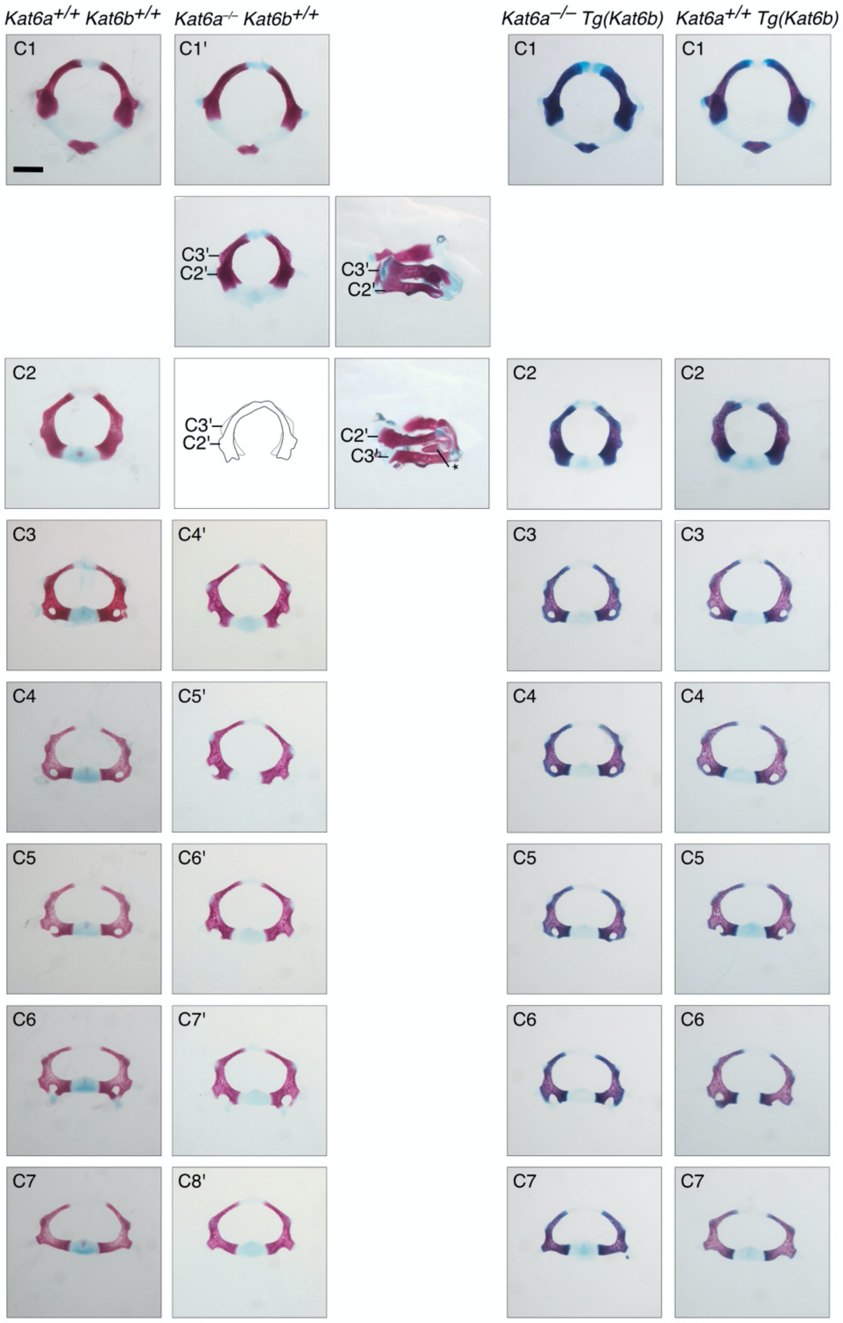

Supplementary Figure 6: Wild type, *Kat6a*$^{-/-}$, *Kat6a*$^{-/-}$*Tg(Kat6b* and *Tg(Kat6b* cervical vertebrae

Representative images of alizarin red (bone) and alcian blue (cartilage) stained cervical vertebrae preparations dissected from N = 9 *Kat6a*$^{+/+}$*Kat6b*$^{+/+}$, 1 *Kat6a*$^{-/-}$*Kat6b*$^{+/+}$, 4 *Kat6a*$^{-/-}$ *Tg(Kat6b)* and 5 *Kat6a*$^{+/+}$*Tg(Kat6b)* E18.5 foetuses. Note that the *Kat6a*$^{-/-}$*Kat6b*$^{+/+}$ foetus has a total 8 cervical vertebrae with C2 and C3 fused, whereas all other genotypes have the normal number of seven cervical vertebrae.

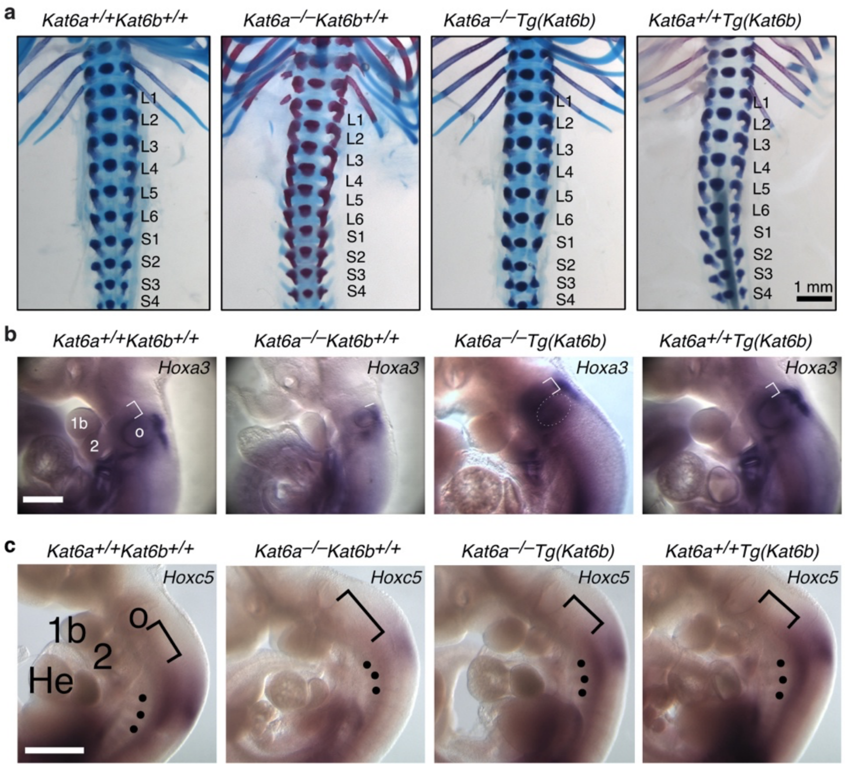

Supplementary Figure 7: Lumber skeleton and Hoxa3 whole mount in situ hybridization from wild type ***Kat6a*** $^{+/+}$**,** ***Kat6a*** $^{-/-}$**,** ***Kat6a*** $^{-/-}$ ***Tg(Kat6b*** **and** ***Tg(Kat6b.***

(a) Representative images of alizarin red (bone) and alcian blue (cartilage) stained skeletal preparations of N = 9 *Kat6a*$^{+/+}$*Kat6b*$^{+/+}$, 1 *Kat6a*$^{-/-}$*Kat6b*$^{+/+}$, 4 *Kat6a*$^{-/-}$*Tg(Kat6b)* and 5 *Kat6a*$^{+/+}$*Tg(Kat6b)* from E18.5 foetuses. Note that the homeotic transformation present in *Kat6a*$^{-/-}$*Kat6b*$^{+/+}$ foetuses in the 19 cervical and thoracic vertebrae does not extend to the lumber regions.

(b) Representative images of whole-mount RNA/RNA in situ hybridisation detecting *Hoxa3* mRNA (purple) in E10.5 *Kat6a*$^{+/+}$*Kat6b*$^{+/+}$, *Kat6a*$^{-/-}$*Kat6b*$^{+/+}$, *Kat6a*$^{-/-}$*Tg(Kat6b)* and *Kat6a*$^{+/+}$*Tg(Kat6b)* embryos. The brackets mark the distance from the anterior expression boundary of *Hoxa3* to the otic vesicle. Scale bar = 370 µm

b) High magnification images of Fig. 4d whole-mount RNA/RNA in situ hybridisation detecting *Hoxc5* mRNA (purple). E10.5 *Kat6a*$^{+/+}$*Kat6b*$^{+/+}$, *Kat6a*$^{-/-}$*Kat6b*$^{+/+}$, *Kat6a*$^{-/-}$

Tg(Kat6b) and *Kat6a*$^{+/+}$*Tg(Kat6b)* embryos. The brackets mark the distance from the anterior expression boundary of *Hoxc5* to the otic vesicle. Scale bar = 680 µm

1b, mandibular portion of the first pharyngeal arch; 2, second pharyngeal arch; He, heart; o, otic vesicle. Scale bar = 370 um in b.

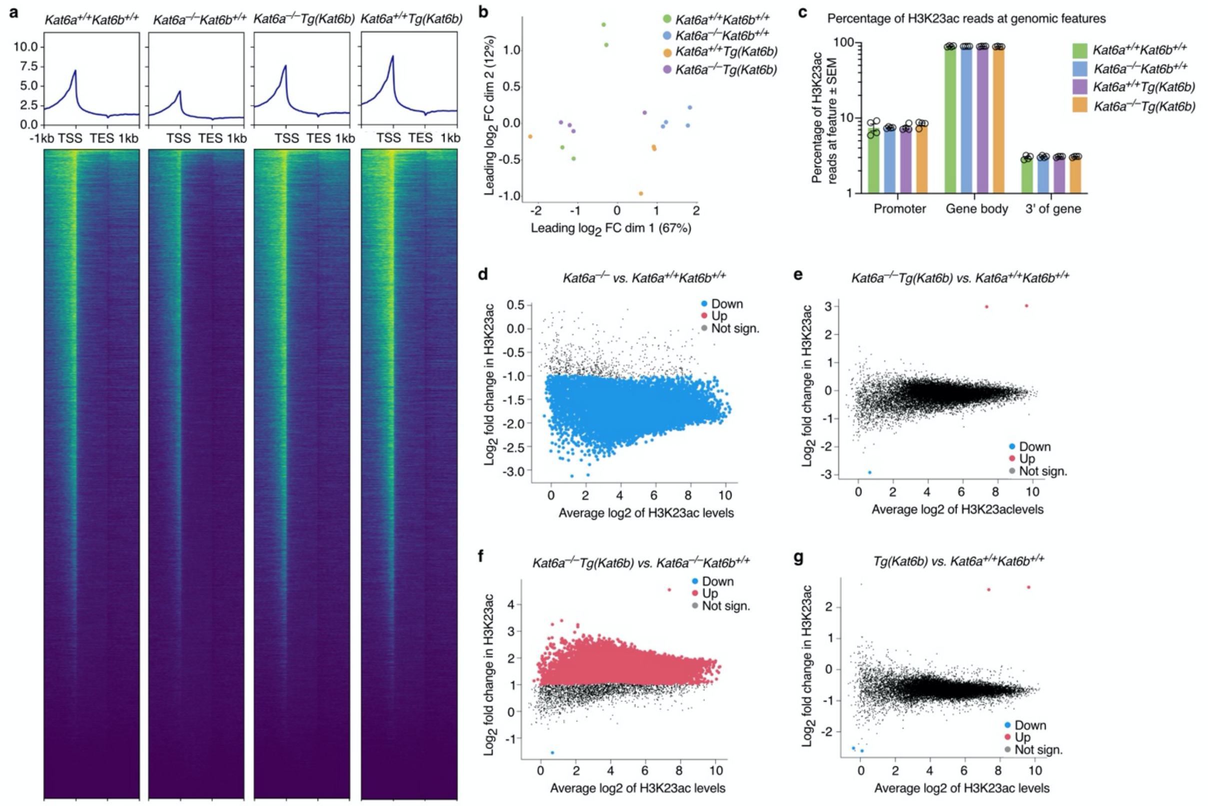

Supplementary Figure 8: CUT&Tag analysis of H3K23ac in *Kat6a*$^{+/+}$, *Kat6a*$^{–/–}$, *Kat6a*$^{–/–}$ *Tg(Kat6b* and *Tg(Kat6b)* cells.

(a-g) CUT&Tag results detecting histone H3 lysine 23 acetylation in (H3K23ac) in primary mouse embryonic fibroblasts isolated from E14.5 *Kat6a*$^{+/+}$*Kat6b*$^{+/+}$, *Kat6a*$^{–/–}$*Kat6b*$^{+/+}$, *Kat6a*$^{–/–}$*Tg(Kat6b)* and *Kat6a*$^{+/+}$*Tg(Kat6b)* E10.5 foetuses. N = 4 foetuses per genotype. Data were analysed as described in the methods section. A false discovery rate (FDR) of less than 0.05 was considered significant.

(a) Coverage plot and probe alignment plot showing high H3K23ac occupancy near the transcription start site.

(b) Multidimensional scaling plot of the leading pairwise comparisons in H3K23ac levels in gene bodies showing *Kat6a*$^{+/+}$*Kat6b*$^{+/+}$, *Kat6a*$^{–/–}$*Kat6b*$^{+/+}$, *Kat6a*$^{+/+}$*Tg(Kat6b)* and *Kat6a*$^{–/–}$*Tg(Kat6b)* MEF samples.

(c) Percentage of H3K23ac reads at the genomic features promoter (-1 kb to TSS), gene body and TES to 1 kb downstream of TES. Note that the distribution of H3K23ac occupancy is similar between genotypes.

(d-g) M (log_2 ratio) and A (mean average, log_2 CPM) plots showing gene bodies decorated with H3K23ac in MEFs isolated from *Kat6a*$^{-/-}$ vs. *Kat6a*$^{+/+}$*Kat6b*$^{+/+}$ (d), *Kat6a*$^{-/-}$*Tg(Kat6b)* vs. *Kat6a*$^{+/+}$*Kat6b*$^{+/+}$ (e), *Kat6a*$^{-/-}$*Tg(Kat6b)* vs. *Kat6a*$^{-/-}$*Kat6b*$^{+/+}$ (f) and *Tg(Kat6b)* vs. *Kat6a*$^{+/+}$*Kat6b*$^{+/+}$ foetuses . Genes with a reduction in H3K23ac are shown in blue, increase in red, unchanged in black. Note the decrease in H3K23ac *Kat6a*$^{-/-}$ vs. *Kat6a*$^{+/+}$*Kat6b*$^{+/+}$ MEFs at almost all gene bodies (d) and that overexpression of *Kat6b* on a *Kat6a* null background restores normal acetylation patterns at H3K23 (e). Comparison of *Kat6a*$^{-/-}$ *Tg(Kat6b)* vs. *Kat6a*$^{-/-}$*Kat6b*$^{+/+}$ shows an increase in H3K23ac at most loci (f). Comparison of *Tg(Kat6b)* vs. *Kat6a*$^{+/+}$*Kat6b*$^{+/+}$ shows that expression of the *Kat6b* transgene has little effect on wild type cells (g).

References

1 Moreno, P. *et al.* Expression Atlas update: gene and protein expression in multiple species. *Nucleic Acids Res* **50**, D129-D140 (2022).

2 Cardoso-Moreira, M. *et al.* Gene expression across mammalian organ development. *Nature* **571**, 505-509 (2019).

Supplementary Table 1: PCR genotyping oligonucleotides

Oligonucleotides	Source	Identifier
Tg(Kat6b) genotyping primers		
5’-CAACTCAATCGACAGCTGGA-3’	This study	SacB L
5’-GGCTTTGTTTGCCGTAATGT-3’		SacB R
Kat6a genotyping primers		
5’-TTCTTGACCTCTGTGTCGTGTGC-3’	Voss et al., 2009	MozA
5’-AGAAGTACAGTGCTTTGGTTTCC-3’		MozB
5’-ATAGGAACTTCATCAGTCAGGTAC-3’		MozC

Supplementary Table 2: Antibodies used for the Flow Cytometry

Antibody	Source	Clone or catalogue number
anti-B220-A700	WEHI	Clone RA3-6B2
anti-CD19-A700	WEHI	Clone 1D3
anti-CD19-PECY7	BD	552854
anti-CD19-Pacific Blue	Biolegend	115523
anti-CD4-A700	WEHI	Clone GK1.5
anti-CD4-APC	BD	553730
anti-CD8-A700	WEHI	Clone 53.6.7
anti-CD8-PE	WEHI	Clone 56.3.7
anti-GR1-A700	WEHI	Clone RB6-8C5
anti-GR1-A594	WEHI	Clone 1A8
anti-Ter119-A700	WEHI	Clone TER-119
anti-LyG6-A700	WEHI	Clone LyG6
anti-SCA1-A594	WEHI	Clone E13
anti-cKIT-PerCPCy5.5	BD	560557
anti-CD48-PECY7	BD	560731
anti-CD150-A647	Biolegend	115918
anti-CD34-FITC	eBioscience	11-03410-82
anti-CD16/32-PECY7	BD	01317
anti-CD16/32-APC	ThermoFisher	17-0161-82
anti-CD127/IL7Rα-PE	WEHI	Clone A7R34
anti-CD45.1-BV650	BD	56754
anti-CD45.1-PECy7	Biolegend	110730
anti-CD45.2-FITC	WEHI	
anti-CD45.2-A647	WEHI	Clone s450-15-2
anti-CD45.2-PECy7	ThermoFisher	25-0454-82
anti-IgM-FITC	WEHI	Clone 5.1
anti-IgM A647	WEHI	
anti-IgD-PE	Biolegend	405706
Fluorogold (viability)	Milipore	AB153-I

Supplementary Table 3: Cell surface markers used for flow cytometry

Tissue type	Cell type	Markers
Bone marrow and foetal liver	LSK	$Lineage^{-}Sca1^{+}c\text{-}Kit^{+}$
	HPC1	$Lineage^{-}Sca1^{+}c\text{-}Kit^{+}CD48^{+}CD150^{-}$
	HPC2	$Lineage^{-}Sca1^{+}c\text{-}Kit^{+}CD48^{+}CD150^{+}$
	HSC	$Lineage^{-}Sca1^{+}c\text{-}Kit^{+}CD48^{-}CD150^{+}$
	MPP	$Lineage^{-}Sca1^{+}c\text{-}Kit^{+}CD48^{-}CD150^{-}$
Bone marrow	LK	$Lineage^{-}Sca1^{-}c\text{-}Kit^{+}$
	CMP	$Lineage^{-}Sca1^{-}c\text{-}Kit^{+}$CD34+CD16/32–
	GMP	$Lineage^{-}Sca1^{-}c\text{-}Kit^{+}$CD34+CD16/32+
	MEP	$Lineage^{-}Sca1^{-}c\text{-}Kit^{+}$CD34–CD16/32–
	CLP	$Lineage^{-}$ $Sca1^{int}$ $c\text{-}Kit^{int}$ $IL7R\alpha^{+}$
	PreB	$B220^{+}CD19^{+}cKIT^{-}$
	ProB	$B220^{+}CD19^{+}cKIT^{+}IgM^{-}IgD^{-}$
	Immature B	$B220^{+}CD19^{+}cKIT^{-}IgM^{+}IgD^{-}$
	Mature B	$B220^{+}CD19^{+}cKIT^{-}IgM^{+}IgD^{+}$
Peripheral blood	B-cell	$B220^{+}CD19^{+}$
	CD4 T-cell	$CD4^{+}CD8^{-}$
	CD8 T-cell	$CD4^{-}CD8^{+}$
	Neutrophil	$Gr1^{hi}Mac1^{+}$
	Monocyte	$Gr1^{med}Mac1^{+}$

* Lineage negative refers to B220, CD19, CD4, CD8, GR1, LYG6 and TER119; superscripts: int, intermediate; hi, high; med, medium.
