# Supplementary material for: KAT6B overexpression rescues embryonic lethality in homozygous null KAT6A mice restoring vitality and normal lifespan
Source: Nat Commun. 2025 Feb 25;16:1958. doi: 10.1038/s41467-025-57155-4 (PMC11861323; doi:10.1038/s41467-025-57155-4)
Supplement: Supplementary file 2 — Description of Additional Supplementary Files [file 41467_2025_57155_MOESM2_ESM.pdf]

Description of Additional Supplementary Files:

Supplementary Data 1: Trait frequencies described in Arboleda-Tham (KAT6A), SayBarber-Biesecker-Young-Simpson (KAT6B) and Genitopatellar syndrome (KAT6B) patients, separated by study.

Supplementary Data 2: Related to Figure 2. RNA-sequencing data, genes differentially expressed in $Kat6a^{-/-}Kat6b^{+/+}$ vs. $Kat6a^{+/+}Kat6b^{+/+}$ E9.5 embryos.

Supplementary Data 3: Related to Figure 2. RNA-sequencing data, genes differentially expressed in $Kat6a^{-/-}$*Tg(Kat6b)* vs. $Kat6a^{+/+}Kat6b^{+/+}$ E9.5 embryos.

Supplementary Data 4: Related to Figure 2. RNA-sequencing data, genes differentially expressed in $Kat6a^{+/+}$*Tg(Kat6b)* vs. $Kat6a^{+/+}Kat6b^{+/+}$ E9.5 embryos.

Supplementary Data 5: Related to Figure 2. RNA-sequencing data, genes differentially expressed in $Kat6a^{-/-}$*Tg(Kat6b)* vs. $Kat6a^{-/-}Kat6b^{+/+}$ E9.5 embryos

Supplementary Data 6: Related to Figure 2. RNA-sequencing data, genes differentially expressed in $Kat6a^{-/-}$*Tg(Kat6b)* vs. $Kat6a^{+/+}$*Tg(Kat6b)* E9.5 embryos.

Supplementary Data 7: Related to Figure 2. RNA-sequencing data, genes differentially expressed in $Kat6a^{+/+}$*Tg(Kat6b)* vs. $Kat6a^{-/-}Kat6b^{+/+}$ E9.5 embryos.

Supplementary Data 8: Related to Figure 2. RNA-sequencing data, genes differentially expressed in $Kat6a^{-/-}Kat6b^{+/+}$ vs. $Kat6a^{+/+}Kat6b^{+/+}$, $Kat6a^{-/-}$*Tg(Kat6b)* vs. $Kat6a^{+/+}Kat6b^{+/+}$ and $Kat6a^{-/-}$*Tg(Kat6b)* vs. $Kat6a^{-/-}Kat6b^{+/+}$ E9.5 embryos, selected gene families only.

Supplementary Data 9: Related to Figure 6. CUT&Tag analysis of H3K23ac in $Kat6a^{-/-}$ $Kat6b^{+/+}$ vs. $Kat6a^{+/+}Kat6b^{+/+}$ MEFs.

Supplementary Data 10: Related to Figure 6. CUT&Tag analysis of H3K23ac in $Kat6a^{-/-}$ *Tg(Kat6b)* vs. $Kat6a^{+/+}Kat6b^{+/+}$ MEFs.

Supplementary Data 11: Related to Figure 6. CUT&Tag analysis of H3K23ac *Kat6a*$^{+/+}$*Tg(Kat6b)* vs. *Kat6a*$^{+/+}$*Kat6b*$^{+/+}$ MEFs.

Supplementary Data 12: Related to Figure 6. CUT&Tag analysis of H3K23ac in *Kat6a*$^{-/-}$ *Tg(Kat6b)* vs. *Kat6a*$^{-/-}$*Kat6b*$^{+/+}$ MEFs.
