# Supplementary material for: KAT6B overexpression rescues embryonic lethality in homozygous null KAT6A mice restoring vitality and normal lifespan
Source: Nat Commun. 2025 Feb 25;16:1958. doi: 10.1038/s41467-025-57155-4 (PMC11861323; doi:10.1038/s41467-025-57155-4)
Supplement: Supplementary file 15 — Reporting Summary [file 41467_2025_57155_MOESM15_ESM.pdf]

nature portfolio

Corresponding author(s): Tim Thomas

Last updated by author(s): Jan 30, 2025

Reporting Summary

Nature Portfolio wishes to improve the reproducibility of the work that we publish. This form provides structure for consistency and transparency in reporting. For further information on Nature Portfolio policies, see our Editorial Policies and the Editorial Policy Checklist.

Statistics

For all statistical analyses, confirm that the following items are present in the figure legend, table legend, main text, or Methods section.

n/a	Confirmed	
☐	☒	The exact sample size (*n*) for each experimental group/condition, given as a discrete number and unit of measurement
☒	☐	A statement on whether measurements were taken from distinct samples or whether the same sample was measured repeatedly
☐	☒	The statistical test(s) used AND whether they are one- or two-sided<br>*Only common tests should be described solely by name; describe more complex techniques in the Methods section.*
☐	☒	A description of all covariates tested
☐	☒	A description of any assumptions or corrections, such as tests of normality and adjustment for multiple comparisons
☐	☒	A full description of the statistical parameters including central tendency (e.g. means) or other basic estimates (e.g. regression coefficient) AND variation (e.g. standard deviation) or associated estimates of uncertainty (e.g. confidence intervals)
☒	☐	For null hypothesis testing, the test statistic (e.g. *F*, *t*, *r*) with confidence intervals, effect sizes, degrees of freedom and *P* value noted<br>*Give P values as exact values whenever suitable.*
☒	☐	For Bayesian analysis, information on the choice of priors and Markov chain Monte Carlo settings
☒	☐	For hierarchical and complex designs, identification of the appropriate level for tests and full reporting of outcomes
☒	☐	Estimates of effect sizes (e.g. Cohen's *d*, Pearson's *r*), indicating how they were calculated

Our web collection on statistics for biologists contains articles on many of the points above.

Software and code

Policy information about availability of computer code

Data collection	No software was used to collect data.
Data analysis	RNA-seq: reads were aligned using Rsubread. Differential expression analyses were performed using the edgeR and limma software packages.<br>CUT&Tag: Coverage plots were generated using deepTools. Differential abundance between groups was the assessed using limma and voom software packages.<br>These are previously published, appriately cited and open software sources.

For manuscripts utilizing custom algorithms or software that are central to the research but not yet described in published literature, software must be made available to editors and reviewers. We strongly encourage code deposition in a community repository (e.g. GitHub). See the Nature Portfolio guidelines for submitting code & software for further information.

Data

Policy information about availability of data

All manuscripts must include a data availability statement. This statement should provide the following information, where applicable:

- Accession codes, unique identifiers, or web links for publicly available datasets
- A description of any restrictions on data availability
- For clinical datasets or third party data, please ensure that the statement adheres to our policy

All primary RNA-seq and CUT&Tag data has been submitted to the NCBI GEO database: GSE287243; GSE287244

Research involving human participants, their data, or biological material

Policy information about studies with human participants or human data. See also policy information about sex, gender (identity/presentation), and sexual orientation and race, ethnicity and racism.

Reporting on sex and gender	No human subjects were used in this study.
Reporting on race, ethnicity, or other socially relevant groupings	No human subjects were used in this study.
Population characteristics	No human subjects were used in this study.
Recruitment	No human subjects were used in this study.
Ethics oversight	No human subjects were used in this study.

Note that full information on the approval of the study protocol must also be provided in the manuscript.

Field-specific reporting

Please select the one below that is the best fit for your research. If you are not sure, read the appropriate sections before making your selection.

- [x] Life sciences
- [ ] Behavioural & social sciences
- [ ] Ecological, evolutionary & environmental sciences

For a reference copy of the document with all sections, see nature.com/documents/nr-reporting-summary-flat.pdf

Life sciences study design

All studies must disclose on these points even when the disclosure is negative.

Sample size	Pilot experiments were performed to determine sample size needed for statisitically valid results.
Data exclusions	No data were excluded.
Replication	We used multiple biological replicates to ensure that the data are replicable.
Randomization	Embryos and foetuses were collected as they occurred in their litters, therefore random. Dissection and examination occurred before genotyping and therefore the operator was blinded to genotypes for these assessments. After genotyping, test embryos were assigned for further experiments with developmental stage-matched controls. Test foetuses were used with littermate controls. Where possible, assays were performed using automated quantitation, e.g., western blots (Odyssey Imaging System, Li-COR) and flow cytometry analysis (same gates used on each sample, FlowJo analysis software). Bioinformatics was performed by an independent bioinformatics facility at our institute.
Blinding	Dissection and examination of embryos and foetuses occurred before genotyping and therefore the operator was blinded to genotypes for these assessments. Blinding was not possible in cases were the embryos needed to be genotyped prior to the experiment being performed. In these cases test embryos were used with developmental stage-matched to controls. Bioinformatic analyses were performed independently by our institute's bioinformatics core facility. Automated methods were used in other experiments where possible.

Reporting for specific materials, systems and methods

We require information from authors about some types of materials, experimental systems and methods used in many studies. Here, indicate whether each material, system or method listed is relevant to your study. If you are not sure if a list item applies to your research, read the appropriate section before selecting a response.

Materials & experimental systems

n/a	Involved in the study	
	x	Antibodies
x		Eukaryotic cell lines
x		Palaeontology and archaeology
	x	Animals and other organisms
x		Clinical data
x		Dual use research of concern
x		Plants

Methods

n/a	Involved in the study	
x		ChIP-seq
	x	Flow cytometry
x		MRI-based neuroimaging

Antibodies

Antibodies used	H3K9ac (Epicypher, 13-0033; dilution 1:5000),<br>H3K14ac (Abcam, ab52946; 1:1000)<br>H3K23ac (Millipore, 07-355; 1:5000)<br>pan H3 (Abcam, 10799; 1:5000)<br>goat anti-mouse IgG secondary (IRDye® 800 CW; LI-COR Biosciences 926-32210; 1:10,000)<br>goat anti-rabbit IgG (IRDye®; LI-COR Biosciences, 926-68071; 1:10,000)<br>anti-B220-A700, WEHI Clone RA3-6B2<br>anti-CD19-A700, WEHI Clone 1D3<br>anti-CD19-PECY7 BD 552854<br>anti-CD19-Pacific Blue Biolegend 115523<br>anti-CD4-A700 WEHI Clone GK1.5<br>anti-CD4-APC BD 553730<br>anti-CD8-A700 WEHI Clone 53.6.7<br>anti-CD8-PE WEHI Clone 56.3.7<br>anti-GR1-A700 WEHI Clone RB6-8C5<br>anti-GR1-A594 WEHI Clone 1A8<br>anti-Ter119-A700 WEHI Clone TER-119<br>anti-LyG6-A700 WEHI Clone LyG6<br>anti-SCA1-A594 WEHI Clone E13<br>anti-cKIT-PerCPCy5.5 BD 560557<br>anti-CD48-PECY7 BD 560731<br>anti-CD150-A647 Biolegend 115918<br>anti-CD34-FITC eBioscience 11-03410-82<br>anti-CD16/32-PECY7 BD 01317<br>anti-CD16/32-APC ThermoFisher 17-0161-82<br>anti-CD127/IL7R-PE WEHI Clone A7R34<br>anti-CD45.1-BV650 BD 5 6754<br>anti-CD45.1-PECy7 Biolegend 110730<br>anti-CD45.2-FITC WEHI<br>anti-CD45.2-A647 WEHI Clone s450-15-2<br>anti-CD45.2-PECy7 ThermoFisher 25-0454-82<br>anti-IgM-FITC WEHI Clone 5.1<br>anti-IgM A647 WEHI<br>anti-IgD-PE Biolegend 405706
Validation	All histone antibodies were validated by the manufacturer. FACs antibodies are all standard diagnostic monoclonal antibodies sold by multiple companies as well as being produced in house.

Animals and other research organisms

Policy information about studies involving animals; ARRIVE guidelines recommended for reporting animal research, and Sex and Gender in Research

Laboratory animals	Mus Musculus, Kat6a-/- mice and Kat6b transgenic mice
Wild animals	We did not use wild animals
Reporting on sex	Equal numbers of male and female mouse embryos and foetuse were used in order of occurrance in the litters dissected. No differences in male or female Kat6a–/– Kat6b transgenic rescue were observed. Sex based analysis was performed in Figure 7b as indicated. In RNA-seq experiments and CUT&Tag Xist was used to determine/confirm sex of embryos as indicated in the methods section.
Field-collected samples	No samples were collected in the field.
Ethics oversight	All animal experiments were conducted with approval of the WEHI Animal Ethics Committee and according to the Australian code for the care and use of animals for scientific purposes.

Note that full information on the approval of the study protocol must also be provided in the manuscript.

Plants

Seed stocks	No plants were used in this study.
Novel plant genotypes	No plants were used in this study.
Authentication	No plants were used in this study.

Flow Cytometry

Plots

Confirm that:

- [x] The axis labels state the marker and fluorochrome used (e.g. CD4-FITC).
- [x] The axis scales are clearly visible. Include numbers along axes only for bottom left plot of group (a 'group' is an analysis of identical markers).
- [x] All plots are contour plots with outliers or pseudocolor plots.
- [x] A numerical value for number of cells or percentage (with statistics) is provided.

Methodology

Sample preparation	Erythrocytes were lysed by washing samples 2x 10 ml in a hypotonic solution (150 mM NH4Cl, 0.1 mM EDTA, 12 mM NaHCO3, pH 7.2). Cells were resuspended in a FACS buffer (150 nM NaCl, 3.7 mM KCl, 2.5 mM CaCl22H2O, 1.2 mM MgSO4•7H2O, 0.8 mM K2HPO4, 1.2 mM KH2PO4, 11.5 mM HEPES, pH 7.4) supplemented with 2% foetal calf serum and stained with conjugated antibodies (Supplementary Table 11) for 1 h on ice. Samples were washed in 3-4 ml FACS buffer and analysed on a flow cytometer (BD LSRFortessaTM X-20, BD) at < 7500 events/sec. Data were analysed using flow cytometry analysis software (FlowJo version 10.7, Tree Star Inc.). Cell surface markers used to identify individual cell types are shown in Supplementary Table 12.
Instrument	BD LSRFortessaTM X-20, BD
Software	FlowJo version 10.7, Tree Star Inc
Cell population abundance	Cells were analysed not sorted
Gating strategy	Foetal liver donor (CD45.1+) cells vs. recipient (CD45.1/2+) are shown for the total live cell population and each peripheral blood cell population analysed.

- [x] Tick this box to confirm that a figure exemplifying the gating strategy is provided in the Supplementary Information.
