# Supplementary material for: KAT6B overexpression rescues embryonic lethality in homozygous null KAT6A mice restoring vitality and normal lifespan
Source: Nat Commun. 2025 Feb 25;16:1958. doi: 10.1038/s41467-025-57155-4 (PMC11861323; doi:10.1038/s41467-025-57155-4)
Supplement: Supplementary file 16 — Source Data [file 41467_2025_57155_MOESM16_ESM.pdf]

nature portfolio

Peer Review File

KAT6B overexpression rescues embryonic lethality in homozygous null KAT6A mice restoring vitality and normal lifespan

Corresponding Author: Professor Tim Thomas

This file contains all reviewer reports in order by version, followed by all author rebuttals in order by version.

Version 0:

Reviewer comments:

Reviewer #1

(Remarks to the Author)
Bergamasco et al address the important question of functional redundancy in vivo of KAT6A and KAT6B. Like any other homologous pairs of lysine acetyltransferases, the KATs share almost identical sequences, structures, and biochemical properties. They also incorporate into the same complexes, in a mutually exclusive way. However, it is not clear whether the pairs are functionally redundant in vivo. Mutations in mice generate different phenotypes, which could reflect either differences in expression levels in particular tissues at particular times, or differences in molecular functions. The question of redundancy is of increasing importance as small molecule inhibitors of these enzymes are developed as therapies for cancer and other maladies.

The authors develop mouse models that over express KAT6B (4X) in mice that are either WT or null for KAT6A. Their data clearly demonstrate that KAT6B expression can rescue the molecular and developmental defects caused by loss of KAT6A. Differences in the KAT6A and KAT6B phenotypes, then, likely reflect imbalances in expression of the two KATs in specific tissues, e.g.hematopoietic stems vs the brain.

The experiments are well executed and they support the conclusions drawn. The results are important, as they contribute to understanding similarities in human conditions associated with deficiencies in KAT6A and KAT6B, as well as inform strategies fro development of KAT6i. The paper will be of interest to wide audience, including those interested in developmental disorders, cancer, chromatin, and gene regulation.

A couple of suggestions regarding presentation of the data:

1. While the data in Figure 1b are interesting, they are a bit tangential to the story in this paper. It would be more informative for interpretation of the mouse studies to have a representation of the relative expression levels of KAT6A and B in different mouse tissues during development.

2. Fig 3 is dense and some panels are difficult to read, even after zooming in online. Could some of the lesser critical panels be moved to supplemental? The interior labels in Fig 3e,f are indecipherable.

Reviewer #2

(Remarks to the Author)
The study Bergamasco et al. demonstrates that overexpression of KAT6B in a KAT6A knockout mouse background is able to grossly reverse the KAT6A knockout phenotypes. This is an important and interesting finding, because it demonstrates that in this case a closely related paralog can almost fully compensate the loss of a protein. The authors show substantial

amount of experimental evidence that the KAT6B transgene indeed is able to reverse the effect of the KAT6A knockout at a cellular and organismal level. The major conclusions are supported by the presented experiments.
However, the manuscript falls short regarding the molecular mechanism how KAT6B is able to do this. Specifically, it would be important to show stronger evidence that KAT6B can reverse the chromatin landscape in the KAT6A knockout cells. Further, giving the high similarity between KAT6A and KAT6B it is in my view not surprising that overexpression of KAT6B can compensate for the genetic loss of KAT6A, although loss of each individual protein results in distinct phenotypic consequences. Genetic compensation in response to a gene knockout of highly related family members is known for many years (reviewed for instance in El-Brolosy and Stainier, PLOS Gen, 2017) and there are multiple examples where one paralogue compensates for the loss of a related protein family member by upregulation the endogenous protein level, for instance, RING1A and RING1B (Endoh et al., Dev., 2008), HDAC1 and HDAC2 (Yamaguchi et al., Gen&Dev, 2010) or MEF2A and MEF2D (Majidi et al., Cell Rep, 2019). It is quite an overstatement that this study is the first demonstration of compensation of highly related chromatin regulators, as stated by the authors in the introduction.
Therefore, I think the manuscript in its present form does not match the scope and requirements for publication in Nature Communications and is more suitable for a more specialized journal.

Major Points:
1. The author's claim that that the KAT6B transgene is 4-fold overexpressed bases only on a RT-qPCR experiment. This should also be shown on protein level.
2. Histone acetylation level has only been performed on bulk chromatin, which is not very informative (and often mis-leading). This should be shown at several specific genes via ChIP-qPCR in MEFs. Even better would be to investigate this aspect at a genome wide-level via ChIP-Seq.
How has the quantifications of the histone marks been performed? They Westerns appear to be much more variable compared to the quantification, even when normalized to pan-H3 (especially in 3e). In Figure 3e lanes 3 and 6 appear to be rather similar, yet in the quantification there is a big difference.
3. Recent work suggests that KAT6A and KAT6B likely target similar loci via a DNA binding domain at the N-terminus of the proteins (PMID: 36537216, 36754959). Analysis of the chromatin binding pattern of KAT6A and KAT6B in MEFs would be important to explain the ability to compensate each other. If this is not possible, this aspect should at least be discussed.
4. The claim that the KAT6B transgene reverses the expression of the KAT6A KO is not convincing. In the PCA in Figure 3a the dimension 1 only contributes to 22% of the total changes. Also, both the plots in 3b and 3c clearly demonstrate that the reversal is only partial. The text as well as the title "Kat6b overexpression reversed gene expression changes present in Kat6a–/– 5 E9.5 embryos" is therefore mis-leading and should be revised.
5. Regarding the altered GO terms in Figure 3e and 3e: I would recommend to also investigate these changes via GSEA, the results may become more compelling. Also, in Figure 3f something appears to be wrong (negative number of affected genes??). Have the X and Y-axis been swapped? Also, it is typically more common to use -log(p-value) and not log(p-value)
6. I may also be worth to have a closer look on the gene expression changes Kat6a+/+ Tg(Kat6b) vs. wildtype, to address the effect of KAT6B overexpression.
7. In my view, the discussion falls short to explain how KAT6B is able to compensate for KAT6A, and should be improved.

Minor points:
1. Figure 1a: Why are some of the exons (grey, white) thinner than the others? In the event the authors want to show that these regions do not have domains: Please check out the Alphafold database. There is a domain at the very N-terminus and neither the acidic nor the serine and methionine-rich regions are domains in the classical sense.
I would discourage the usage of the term "NEMM", because it is outdated and not very informative. Recent work suggests that the NEMM consists of two separate winged-helix domains (PMID: 36754959, 36537216). Showing these two domains in Figure 1a would be more useful.
The similarity between certain domains/regions are shown. What is the similarity of the acidic part? What is the similarity (and identity) globally?
2. "Overexpression of Kat6b in Kat6a–/– Tg(Kat6b) embryos reduced the number of differentially expressed genes compared to Kat6a+/+Kat6b+/+ embryos to 285 genes, 170 downregulated and 121 upregulated". 170 + 121 = 291, and not 285.
3. The title of the supplementary data is different from that of the main article. (Personally, I find the title of the supplementary better)
4. In many figures the fonts are very small and hard to read. I recommend increasing the fonts sizes.

Reviewer #3

(Remarks to the Author)
The manuscript by Bergamasco et al examines the role of closely related paralog function, Kat6a and Kat6b in hematopoietic stem cells and skeletal development. Specifically, the authors tested the idea that overexpression of one paralog, Kat6b, would be able to rescue the Kat6a developmental defects. Given the high similarity, the authors show that H3K23ac and H3K9ac are rescued to wildtype levels, as well as fewer numbers of gene expression changes between rescue and wildtype and normalized hematopoietic stem cells. In addition, all aspects of skeletal and palate development are rescued as well as viability. The paper describes a very interesting and novel phenomenon between closely related paralogs which is unique. It is well written and illustrated and is of interest to the readers of Nature Communications. There are a few suggestions that would strengthen the manuscript. My comments are below:

1.The overall acetylation and morphological rescue is interesting. Does Kat6b overexpression rescue Kat6b mutants? What

is the overexpression phenotype of Kat6b without Kat6a in the background? These are important control for functionality of Kat6b and that it is/ is not taking on a new function.

2. This brings up the interesting observation that in the Kat6a+/+; Tg(Kat6b) animlas there is a significant increase in H3K9 acetylation but seeming no phenotype in the embryos. Is this true of is there some phenotype in the mice morphologically? Quantification of the size for the skeletal elements would be important here to determine this. What do the authors think is the explanation for not having an overexpression phenotype? Is there a threshold that needs to be reached to cause an overexpression phenotype? The discussion section is a good start, but more information on this would be useful including references if available. It suggests that Kat6b can rescue when Kat6a is absent but is not able to add to the levels for overexpression. Similarly it would be interesting to know if this is true without Kat6a in the background (see point 1).

3. For the gene expression, the RNAseq clearly shows that gene expression changes are more normalized and similar to wildtype Kat6a and Kat6b. However from the heat map, there are a significant number of genes that remain upregulated and downregulated following the cross with Tg(Kat6b). It would be interesting to show what these genes are. And while validation of the sequencing results are not necessarily required, it would be very interesting to show the expression by in situ of some of the genes that are normalized and the ones that not rescued, especially in relation to the homeotic transformation data (ie Hox genes). It would be important to prove that this is indeed the case in the embryo by looking at gene expression at earlier time points.

4. The authors present that there elevated Cd8 and reduced Cd4 T cell levels in Kat6a+/+; Tg(Kat6b) animals. What is the significance of this? And why does it happen specifically in this population? Please expand the discussion around this.

Minor:
1. Would be very helpful to keep the same colors/position for each genetic condition in every graph.

Version 1:

Reviewer comments:

Reviewer #2

(Remarks to the Author)
The authors did an overall great job to improve the manuscript. They convincingly addressed all reviewers questions. I have only one minor point: please make the Cut&Tag data publicly available.

Reviewer #3

(Remarks to the Author)
The authors have carefully addressed the concerns of the previous review. The topic of genetic redundancy is of importance to the field, and they begin to tease out the mechanism of how this occurs. The addition of the new data strengthens the paper significantly and I appreciate the answers to my questions. A couple minor suggestions/clarification is needed on the new data.
The in situ hybridization is a nice addition to the figure. It would be important to include some higher magnification insets especially for the hoxc5 since it is difficult to see the difference here. Similarly, the Cut and Run data nicely shows changes in specific loci (Figure 6e-g). Interestingly the levels of the peaks are increased in the Kat6a+/+ Tg(kat6b)+/+, getting back to the question of why there is no phenotype in the overexpression. It would be great to add text to the discussion section around this issue of dose and/or lack of cofactors etc, as it will be important to determine to refine the mechanism.

Open Access This Peer Review File is licensed under a Creative Commons Attribution 4.0 International License, which permits use, sharing, adaptation, distribution and reproduction in any medium or format, as long as you give appropriate credit to the original author(s) and the source, provide a link to the Creative Commons license, and indicate if changes were made.
In cases where reviewers are anonymous, credit should be given to 'Anonymous Referee' and the source.
The images or other third party material in this Peer Review File are included in the article's Creative Commons license, unless indicated otherwise in a credit line to the material. If material is not included in the article's Creative Commons license and your intended use is not permitted by statutory regulation or exceeds the permitted use, you will need to obtain permission directly from the copyright holder.
To view a copy of this license, visit https://creativecommons.org/licenses/by/4.0/

Point-by-point responses to the reviewers' comments for Nature Communications manuscript NCOMMS-23-51506-T

We would like to thank the reviewers for their insightful comments and for the time and effort spent on reviewing our paper. We believe that in responding to the reviewers' comments with additional experiments and changes to the text of the manuscript, our study has been much improved.

The additional experimental data have resulted in 37 new figure panels displayed in Figures 1 and 4, as well as Supplemental Figures 1 and 7, and in 4 entirely new figures, namely Figure 6 and Supplemental Figures 4, 6 and 8, as well as 4 entirely new Supplemental Tables 9 to 12. We hope that the reviewers and the editor are satisfied with our additional work.

Reviewer #1 (Remarks to the Author):
Bergamasco et al address the important question of functional redundancy in vivo of KAT6A and KAT6B. Like any other homologous pairs of lysine acetyltransferases, the KATs share almost identical sequences, structures, and biochemical properties. They also incorporate into the same complexes, in a mutually exclusive way. However, it is not clear whether the pairs are functionally redundant in vivo. Mutations in mice generate different phenotypes, which could reflect either differences in expression levels in particular tissues at particular times, or differences in molecular functions. The question of redundancy is of increasing importance as small molecule inhibitors of these enzymes are developed as therapies for cancer and other maladies.

The authors develop mouse models that over express KAT6B (4X) in mice that are either WT or null for KAT6A. Their data clearly demonstrate that KAT6B expression can rescue the molecular and developmental defects caused by loss of KAT6A. Differences in the KAT6A and KAT6B phenotypes, then, likely reflect imbalances in expression of the two KATs in specific tissues, e.g.hematopoietic stems vs the brain.

The experiments are well executed and they support the conclusions drawn. The results are important, as they contribute to understanding similarities in human conditions associated with deficiencies in KAT6A and KAT6B, as well as inform strategies fro development of KAT6i. The paper will be of interest to wide audience, including those interested in developmental disorders, cancer, chromatin, and gene regulation.

A couple of suggestions regarding presentation of the data:

1. While the data in Figure 1b are interesting, they are a bit tangential to the story in this paper. It would be more informative for interpretation of the mouse studies to have a representation of the relative expression levels of KAT6A and B in different mouse tissues during development.

Response:
As the reviewer requested, we have removed Figure 1b, and placed it in a supplemental figure. We have created a new Figure 1b which shows *Kat6a* compared to *Kat6b* mRNA in whole mouse embryos during development from E8.5 to E10.5 and in heart development, pharyngeal arch development, as well as MEFs and hematopoietic stem and progenitor cells (LSK cells) from the foetal liver. These are the cells and tissues most relevant to the tissues and organs most strongly affected by the loss of KAT6A function. In addition, we have presented data comparing *Kat6a* and *Kat6b* mRNA levels from publicly accessible datasets in Supplemental Figure 1. In

order to allow us space for other new data requested, we have combined the original Figure 1 with the original Figure 2 together with the new Figure1b.

2. Fig 3 is dense and some panels are difficult to read, even after zooming in online. Could some of the lesser critical panels be moved to supplemental? The interior labels in Fig 3e,f are indecipherable.

Response:
As requested, we have enlarged the font size in the new Figures 2, 3 and 7, including the internal font size in the original Figure 3e,f, which is now Figure 2e,f.

*****

Reviewer #2 (Remarks to the Author):
The study Bergamasco et al. demonstrates that overexpression of KAT6B in a KAT6A knockout mouse background is able to grossly reverse the KAT6A knockout phenotypes. This is an important and interesting finding, because it demonstrates that in this case a closely related paralog can almost fully compensate the loss of a protein. The authors show substantial amount of experimental evidence that the KAT6B transgene indeed is able to reverse the effect of the KAT6A knockout at a cellular and organismal level. The major conclusions are supported by the presented experiments.

However, the manuscript falls short regarding the molecular mechanism how KAT6B is able to do this. Specifically, it would be important to show stronger evidence that KAT6B can reverse the chromatin landscape in the KAT6A knockout cells0.

Response:
In order to address this important point, we have performed an extensive CUT&Tag experiment to assay the histone modification catalysed by KAT6A, this involved the analysis of 4 independent samples from each of the 4 genotypes, *Kat6a*$^{+/+}$*Kat6b*$^{+/+}$, *Kat6a*$^{–/–}$ *Kat6b*$^{+/+}$, *Kat6a*$^{–/–}$*Tg(Kat6b)* and *Kat6a*$^{+/+}$*Tg(Kat6b*. This investigation has resulted in 1 new main figure (Figure 6), 1 new supplemental figure (Supplemental Figure 8), described in results section page 14 and discussed on 16. The experimental details are described in the methods section on page 23 and 24 and the statistical analysis on pages 25 and 26. We show that at key developmental genes relevant to the *Kat6a*$^{–/–}$ mutant phenotype, i.e. genes coding for HOX, TBX and DLX transcription factors, there is a substantial decrease in H3K23ac in the *Kat6a*$^{–/–}$ *Kat6b*$^{+/+}$ (*Kat6a* knockout) condition compared to *Kat6a*$^{+/+}$*Kat6b*$^{+/+}$ (wild type) samples and this is restored to wild type levels in the rescued *Kat6a*$^{–/–}$*Tg(Kat6b)* samples. Quite remarkably, in our opinion, even individual, minor peaks are restored so that the transgene-rescued cells have an H3K23ac pattern indistinguishable from the wild-type condition.

Further, giving the high similarity between KAT6A and KAT6B it is in my view not surprising that overexpression of KAT6B can compensate for the genetic loss of KAT6A, although loss of each individual protein results in distinct phenotypic consequences. Genetic compensation in response to a gene knockout of highly related family members is known for many years (reviewed for instance in El-Brolosy and Stainier, PLOS Gen, 2017) and there are multiple examples where one paralogue compensates for the loss of a related protein family member by upregulation the endogenous protein level, for instance, RING1A and RING1B (Endoh et al., Dev., 2008), HDAC1 and HDAC2 (Yamaguchi et al., Gen&Dev, 2010) or MEF2A and MEF2D (Majidi et al., Cell Rep, 2019). It is quite an overstatement that this study is the first demonstration of compensation of highly related chromatin regulators, as stated by the authors in the introduction. Therefore, I think the manuscript in its present form does not match the scope and

requirements for publication in Nature Communications and is more suitable for a more specialized journal.

Response:
We agree that the clause "*representing the first demonstration of complete compensation"* was not precisely worded and therefore may have appeared to be an overstatement. We have changed the wording to a more precise and more moderate statement of the key results: "*These data indicate that KAT6B can completely replace loss of essential KAT6A functions, if expressed at sufficiently high levels, despite KAT6B not normally regulating critical processes dependent on KAT6A. "*

We disagree with the reviewer's view that it is *"not surprising that overexpression of Kat6b can compensate for the genetic loss of Kat6a"* for two reasons:

(A) The literature on KAT6A and KAT6B to this point shows that these closely related proteins have acquired distinct functions during evolution, as indicated by their different knockout phenotypes and the nature of the human congenital disorders manifest when these genes are mutated. So, we regard it as surprising that over expression of KAT6B will rescue all major KAT6A knock out phenotypes examined. We go into more detail on the differences in KAT6A and KAT6B knockout phenotypes below the next section.

(B) We believe that whether overexpression of one chromatin modifier rescues the knockout phenotype of a related chromatin modifier has not been explored comprehensively. The examples that the reviewer listed in the statement above do not report a rescue of a lethal phenotype that results in a normal adult mouse, which is fertile and has a normal lifespan, by ectopic expression. Specifically:

(1) Knock out of *Ring1a* has a mild phenotype with mice surviving to adulthood. *Ring1b* has a very severe phenotype in which homozygous null embryos fail to progress through gastrulation. The authors, Endo et al, (Endoh et al., Dev., 2008) show that RING1A protein is upregulated in the absence of RING1B and this compensates for the loss of RING1A in ES cells. However, clearly, RING1A does not compensate for RING1B during gastrulation and these authors have not overexpressed *Ring1a* to determine if *Ring1a* overexpression is sufficient to rescue the gastrulation phenotype of *Ring1b*.

(2) HDAC1 knock out is mid-gestation lethal and *Hdac2* gene expression is induced in HDAC1 deficient cells but cannot compensate for the loss of this deacetylase, suggesting a unique function for HDAC1 (Lagger et al EMBOJ 2002). Whereas during cardiac development HDAC1 and HDAC2 have redundant functions (Mongomery et al G&D 2007). However, *Hdac2* has not been overexpressed to determine if it is capable of rescuing the mid-gestation lethal phenotype of *Hdac1*$^{-/-}$ mice.

(3) Majidi et al show that MEF2D is the predominant myocyte enhancer factor transcription factor family member in granule cell neurons. In the absence of MEF2D, MEF2A can partially compensate through binding to a subset of MEF2D target genes. However, *Mef2a* has not been overexpressed to determine if it is capable of rescuing all defects caused by lack of *Mef2d.*

(4) Furthermore, El-Brolosy and Stainier, Plos Genetics, state in their conclusion: *"Despite its role in maintaining an organism's robustness, the molecular mechanisms underlying genetic compensation remain poorly understood"* and *"Investigating the molecular mechanisms underlying genetic compensation may help us understand why some mutations cause disease while others do not".* This shows that there is continued interest in the topic of redundancy and compensation. Also noteworthy is that this review does not describe any mammalian study in which the authors specifically test functional redundancy between proteins by generating a transgenic and demonstrates complete rescue of severe phenotypes.

Similar to the studies cited by the reviewer, we have we have recently published a study showing that *Ing4* and *Ing5* have a high degree of redundancy (Mah et al Development 2024). *Ing4* KO mice have no abnormal phenotype during development and *Ing5* KO mice have a cardiac septum defect with 85% penetrance and perinatal lethality. In contrast, the *Ing4/Ing5* double KO causes mid-gestation lethality. So clearly this pair of proteins, like many others, are redundant in almost all their functions. However, in this case we have not tested if ING4 overexpression can rescue the 85% penetrant and perinatal lethal cardiac phenotype of *Ing5* KO mice.

These papers are fine illustrations of the well-established concept of redundancy between paralogs. In general, it is well accepted that paralogous genes arose through two rounds of genome duplication during evolution and that during the course of evolution paralogs have acquired novel functions while often retaining redundant functions, which is thought to contribute to the robustness of embryonic development. Of note, the acquisition of independent functions (being necessary either for completely new functions or for the total volume of function) is thought to be essential for the retention of the duplicated genes. In these examples, although it is clear that compensation takes place, none of these examples show that by manipulating the system it is possible to reverse all phenotypes to the point where the mouse has a normal lifespan.

The issue of redundancy and compensation deserves greater attention from cancer researchers. There are numerous examples of oncogenes and tumour suppressors regulating critical pathways of cell proliferation and differentiation common to many cell types, that, when mutated, produce characteristic cancers with a strong tissue bias.

Here we briefly summarise the findings on KAT6A and KAT6B that show that these closely related proteins have acquired different functions during evolution, as indicated by their knock-out phenotypes.

Please note that the following severe *Kat6a*$^{-/-}$ mutant phenotypes are not present in *Kat6b*$^{-/-}$ mutant mice, but nevertheless rescued in the *Kat6a*$^{-/-}$*Tg(Kat6b)* mice:

(1) 100% of the *Kat6a*$^{-/-}$ mice die in the foetal to perinatal phase on several inbred and mixed genetic backgrounds tested (C57BL/6, FVB, BALB/c, DBA and mixed backgrounds of these). Even 30% of *Kat6a*$^{+/-}$ mice die before weaning. In contrast, *Kat6b*$^{-/-}$ mice are viable and fertile on an outbred background and *Kat6b*$^{+/-}$ are also fully viable and fertile on an inbred background (C57BL/6).

(2) KAT6A is essential for the formation and maintenance of hematopoietic stem cells. No KAT6A, no stem cells. Whereas KAT6B has a comparatively minor role in the hematopoietic stem cell compartment.

(3) Loss of KAT6A results in an extensive anterior homeotic transformation of 19 axial skeleton segments with 100% penetrance. This is a result of the essential function of KAT6A in regulating *Hox* genes expression. In contrast, KAT6B has no discernible role in regulating *Hox* genes and loss of KAT6B has no effect on axial skeleton patterning.

(4) Loss of KAT6A results in an interrupted aortic arch type B and a ventricular septal defect with 100% penetrance on a C57BL/6 background. This is never seen in KAT6B knockout mice.

(5) Loss of KAT6A leads to fibroblast senescence even when grown in 4% O_2. $Kat6b^{-/-}$ fibroblasts grow normally.

(6) Loss of KAT6A results in a global reduction in H3K23ac with decreases in H3K9ac at specific loci. Loss of KAT6B results in reduction in H3K9ac in the developing brain with minor effects on H3K23ac.

Conversely, $Kat6b^{-/-}$ are not observed in $Kat6a^{-/-}$ mice:

(7) Loss of KAT6B results in abnormal growth of the cerebral cortex, which is not seen in $Kat6a^{-/-}$ mice.

We were prompted to perform these experiments as a result of our observations and the results published by others summarized above, concerning KAT6A and KAT6B mutant phenotypes. One paralogue is clearly important for a particular cell type but not at all or less so for other cells or organs. This cannot be explained by the presence or absence of expression, (although there are differences in expression levels) since they are both ubiquitously expressed. As a result of these observations and many others in the literature we decided to formally test the ability of KAT6B (which could be argued to have a milder phenotype during embryogenesis) to compensate for the loss of KAT6A function, since loss of KAT6A completely eliminates key developmental processes listed above. Thus, this is a very stringent test of compensation.

To reiterate, typically, where pairs of paralogs have evolved one of the pair is functionally more important during embryonic development. In none of the examples given has it been shown that at the appropriate level of expression the “minor” paralog can completely rescue the “major” paralog as we have shown in this manuscript where, unexpectedly, overexpression of KAT6B completely rescues the lethal phenotypes resulting from KAT6A knock out.

This is not to say that there aren’t also redundant functions between this pair of paralogs. Nevertheless, these points show that there are substantial differences in physiological function of KAT6A and KAT6B in adults and during embryonic development, in addition to any redundant functions. As a result, we consider it remarkable and surprising that overexpression of KAT6B will completely rescue all major and severe phenotypic consequences of loss of KAT6A.

We agree that we may have poorly worded our statement "first demonstration of compensation ….", and as noted above have changed this statement. We would like to make the distinction between "redundancy" between related pairs of proteins and a rescue of mutant phenotypes by overexpression. While the concept of two closely related proteins being redundant is well established, we are not aware that such a rescue has been demonstrated in a mammalian system and the papers cited by the reviewer do not reveal any incidences of a rescue of mutant phenotypes by overexpression of a "minor paralog", which does not appear to be to be major player in the in the rescued developmental processes.

Major Points:
1. The author's claim that that the KAT6B transgene is 4-fold overexpressed bases only on a RT-qPCR experiment. This should also be shown on protein level.

Response:
We would have loved to examine murine KAT6B and KAT6A at the protein level and have tried this for decades. We have extensively tested all commercially available antibodies and none of them specifically detect the KAT6B protein. In. addition we have attempted to generate polyclonal and monoclonal antibodies against various regions of the KAT6B protein and none of them were specific when tested by western blots and immunocytochemistry using material from KAT6B wild type vs. KAT6B knockout neural stem cells, embryonic brain, MEFs and whole embryos.

2. Histone acetylation level has only been performed on bulk chromatin, which is not very informative (and often mis-leading). This should be shown at several specific genes via ChIP-qPCR in MEFs. Even better would be to investigate this aspect at a genome wide-level via ChIP-Seq.

Response:
As the reviewer requested, we have performed an extensive CUT&Tag experiment to assay the histone modification catalyses by KAT6A, this involve the analysis of 4 independent samples from each of the 4 genotypes, *Kat6a*$^{+/+}$*Kat6b*$^{+/+}$, *Kat6a*$^{-/-}$ *Kat6b*$^{+/+}$, *Kat6a*$^{-/-}$*Tg(Kat6b)* and *Kat6a*$^{+/+}$*Tg(Kat6b*. This investigation has resulted in 1 new main figure (Figure 6), 1 new supplemental figure (Supplemental Figure 8), described in results section page 14 and discussed on 16. The experimental details are described in the methods section on page 23 and 24 and the statistical analysis on pages 25 and 26. We show that at key developmental genes relevant to the *Kat6a*$^{-/-}$ mutant phenotype, i.e. , HOX, TBX and DLX genes, there is a substantial decrease in H3K23ac in the *Kat6a*$^{-/-}$ *Kat6b*$^{+/+}$ condition compared to *Kat6a*$^{+/+}$*Kat6b*$^{+/+}$ samples and this is restored to wild type levels in the rescued *Kat6a*$^{-/-}$*Tg(Kat6b)* samples. Quite remarkably, in our opinion, even individual, minor peaks are restored so that the transgene-rescued cells have an H3K23ac pattern indistinguishable from the wild type condition.

How has the quantifications of the histone marks been performed? They Westerns appear to be much more variable compared to the quantification, even when normalized to pan-H3 (especially in 3e). In Figure 3e lanes 3 and 6 appear to be rather similar, yet in the quantification there is a big difference.

Response:

The original Figure 3 did not contain westerns. We gather the reviewer is referring to Western blots that were displayed in Figure 2, which are now displayed in Figure 1 in the revised version.

As described in the methods section in detail, Western blots were run on an automated Western blotting system that also automates the analysis of the signals. It is a fluorescence-based system using proprietary infra-red dyes, which are also converted colours in the visible spectrum to produce an image output as well as quantitative output. As we are sure the reviewer is aware, automated analysis is considered superior, because it prevents any operator biases. The system used here is the Odyssey Imager system, LI-COR Biosciences, which apart from the machine provides all reagents needed, e.g., nitrocellulose membranes (LI-COR, 926-31090), blocking buffer (Intercept® (PBS); LI-COR, 927-70001), secondary antibodies: goat anti-mouse IgG secondary (IRDye® 800 CW; LI-COR Biosciences 926-32210; 1:10,000), goat anti-rabbit IgG (IRDye®; LI-COR Biosciences, 926-68071; 1:10,000). The quantitation is from the output of the image analysis software. i.e., the signal intensity of the acetylated H3 lysine detected divided by the signal intensity detected using the pan H3 antibody. No additional manipulation of the output was made. The infrared dyes used by the Odyssey Imager system can be found at https://www.licor.com/bio/reagents/irdye-infrared-dyes.

Nevertheless, to alleviate any lingering concerns that the reviewer may have about the Western blot in the previous Figure 2e, we have repeated this particular Western blot. The repeat Western blot is now displayed in Figure 1g.

3. Recent work suggests that KAT6A and KAT6B likely target similar loci via a DNA binding domain at the N-terminus of the proteins (PMID: 36537216, 36754959). Analysis of the chromatin binding pattern of KAT6A and KAT6B in MEFs would be important to explain the ability to compensate each other. If this is not possible, this aspect should at least be discussed.

Response:
We are unable to do this experiment because there are no suitable antibodies to detect mouse KAT6A and KAT6B. As noted in point 1, we have extensively tested all commercially available antibodies and none of them specifically detect the KAT6B protein. In. addition we have attempted to generate polyclonal and monoclonal antibodies against various regions of the KAT6B protein and none of them were specific when tested by western blots and immunocytochemistry using material from KAT6B wild type vs. KAT6B knockout neural stem cells, embryonic brain, MEFs and whole embryos.

As the reviewer requested, we have now included a sentence stating that it would be desirable to examine the genomic distribution of KAT6A and KAT6B, but that this has not been possible with the available antibodies.

4. The claim that the KAT6B transgene reverses the expression of the KAT6A KO is not convincing. In the PCA in Figure 3a the dimension 1 only contributes to 22% of the total changes. Also, both the plots in 3b and 3c clearly demonstrate that the reversal is only partial. The text as well as the title "Kat6b overexpression reversed gene expression changes present in Kat6a–/– 5 E9.5 embryos" is therefore mis-leading and should be revised.

Response:

To make the data in Figure 3a clearer, we will explain them here in detail. In Figure 3a, we display a multidimensional scaling (MDS) plot. Distances on the MDS plot represent leading $\log_2$ fold-change, which is the root-mean-square average of the largest 500 $\log_2$ fold-changes, between each pair of RNA samples, irrespective of their genotype. The percentages stated for dimension 1 (22%) and dimension 2 (15%) do no refer to a percentage of genes differentially expressed between genotypes, but instead to the percentage of the total variation between all samples. The total variation comprises genes that are differentially expressed between genotypes and also genes with RNA levels that happen to be different between any one pair of two samples. Further percentages could be calculated for dimensions 3, 4 etc. What we can derive the following information from the MDS plot: (1) The samples of the same genotype cluster together. This indicates repeatability within genotype. (2) The wild-type samples and the ***Tg(Kat6b)*** are in close proximity, i.e., not very different. (3) The ***Kat6a***$^{-/-}$ samples segregate from all other samples in dimension 1. (4) The *Kat6a*$^{-/-}$*Tg(Kat6b)* samples are not far from the wild-type and ***Tg(Kat6b)*** samples in dimension 1. This represents the rescue. (5) *Kat6a*$^{-/-}$ *Tg(Kat6b)* samples are segregated from all other samples in dimension 2, which represent a gain of new characteristics of the *Kat6a*$^{-/-}$*Tg(Kat6b)* samples, which we have now investigated in more detail (see below).

To address the reviewer's comment we (1) changed the title of this section to "*Kat6b overexpression reversed most gene expression changes present in Kat6a*$^{-/-}$ *E9.5 embryos",* (2) provided more information on the number, percentages and characteristics of the genes that are and are not restored to normal gene expression in the *Kat6a*$^{-/-}$*Tg(Kat6b)* vs. *Kat6a*$^{+/+}$*Kat6b*$^{+/+}$ embryos (new Supplementary Figure 4a to 4f) and (3) provide the number, percentages and characteristics of the genes that are uniquely changed in *Kat6a*$^{-/-}$*Tg(Kat6b)* vs. *Kat6a*$^{+/+}$*Kat6b*$^{+/+}$ embryos and unaffected in *Kat6a*$^{-/-}$ *Kat6b*$^{+/+}$ vs. *Kat6a*$^{+/+}$*Kat6b*$^{+/+}$ embryos (new Supplementary Figure 4g to 4i).

The expression levels of an overwhelming number of genes, 439 of the 482 genes, that are differentially expressed in *Kat6a*$^{-/-}$ *Kat6b*$^{+/+}$ vs. *Kat6a*$^{+/+}$*Kat6b*$^{+/+}$ embryos are not differentially expressed in *Kat6a*$^{-/-}$*Tg(Kat6b)* vs. *Kat6a*$^{+/+}$*Kat6b*$^{+/+}$ embryos. This means 91% of gene expression is restored. To make this clearer, we are now displaying the numbers and percentages of rescued genes in the new Supplementary Figure 4.

The expression of many of these genes in restored completely (see Figure 2g, h, i: previously Figure 3g, h, i), and other genes are restored to a level that is not significantly different from the wild-type embryos. It is important to keep in mind that this level of rescue of gene expression had profound effects. *Kat6a*$^{-/-}$ *Kat6b*$^{+/+}$ foetuses die between E13.5 and birth, depending on the genetic background. In contrast, *Kat6a*$^{-/-}$*Tg(Kat6b)* vs. *Kat6a*$^{+/+}$*Kat6b*$^{+/+}$ mice are viable and fertile. The functional outcome is remarkable. The following three major phenotypic anomalies are rescued. The extensive anterior homeotic transformation affecting 19 body segments is rescued. HOX genes are relevant for this, and their expression is overwhelmingly rescued to normal levels (Figure 2g). The cleft palate is rescued. DLX genes are all rescued to normal levels (Figure 2h). The cardiac septum defects are rescued. The expression of a TBX gene associated with cardiac septum defects, *Tbx5* is rescued (Figure 2i). In contrast, the expression of other TBX genes is not rescued. I think it is important to keep the focus on the developmental

mechanisms that are restored and the rescue of the expression of genes that are relevant to this remarkably complete phenotypic rescue.

To make this point clearer, we have generated a new Supplementary Figure 4. We show that 91% of the genes differentially expressed in *Kat6a*$^{-/-}$ *Kat6b*$^{+/+}$ vs. *Kat6a*$^{+/+}$*Kat6b*$^{+/+}$ embryos are rescued in *Kat6a*$^{-/-}$*Tg(Kat6b)* vs. *Kat6a*$^{+/+}$*Kat6b*$^{+/+}$ embryos (89% of the downregulated genes and 94% of the upregulated genes, Supplemental Figure 4a,b,). In Supplemental Figure 4c,d, we have plotted the few genes that are differentially expressed *Kat6a*$^{-/-}$ *Kat6b*$^{+/+}$ vs. *Kat6a*$^{+/+}$*Kat6b*$^{+/+}$ embryos and not restored to normal levels in *Kat6a*$^{-/-}$*Tg(Kat6b)* vs. *Kat6a*$^{+/+}$*Kat6b*$^{+/+}$ embryos. Only 28 of 245 genes that were downregulated in *Kat6a*$^{-/-}$ *Kat6b*$^{+/+}$ vs. *Kat6a*$^{+/+}$*Kat6b*$^{+/+}$ embryos were not rescued and only 15 of 237 genes that were upregulated in *Kat6a*$^{-/-}$ *Kat6b*$^{+/+}$ vs. *Kat6a*$^{+/+}$*Kat6b*$^{+/+}$ embryos were not rescued. We also provided GO term analyses of the genes that are not rescued, but, note that these analyses are limited by the small number of genes that are not rescued.

In addition, we display in Supplementary Figure 4g to 4i the numbers and characteristics of genes that are differentially expressed uniquely between *Kat6a*$^{-/-}$*Tg(Kat6b)* vs. *Kat6a*$^{+/+}$*Kat6b*$^{+/+}$ embryos. These gene expression differences contribute to the segregation of the *Kat6a*$^{-/-}$ *Tg(Kat6b)* samples in dimension 2 of the MDS plot in Figure 3a. GO term and KEGG pathway analyses identified a small number of molecular or biological signatures that were, however, supported only by changes in a small number of genes, so that statements regarding the function of these genes would not be appropriate.

Lastly, we also display numbers, percentages and characteristics of genes that are differentially expressed between *Kat6a*$^{+/+}$*Tg(Kat6b)* vs. *Kat6a*$^{+/+}$*Kat6b*$^{+/+}$ embryos (Supplementary Figure 4j to 4l). Only 74 genes were differentially expressed between *Kat6a*$^{+/+}$*Tg(Kat6b)* vs. *Kat6a*$^{+/+}$*Kat6b*$^{+/+}$ embryos. Limited pathway information can be derived from these, as the GO term annotation was supported by only a small number of genes.

5. Regarding the altered GO terms in Figure 3e and 3e: I would recommend to also investigate these changes via GSEA, the results may become more compelling. Also, in Figure 3f something appears to be wrong (negative number of affected genes??). Have the X and Y-axis been swapped? Also, it is typically more common to use -log(p-value) and not log(p-value)

Response:
We thank the reviewer for noting the x axis labelling mistake. The axis of Figure 3f have been corrected. This figure element is now in Figure 2f.

As the reviewer requested, we have now included unbiased gene set enrichment analyses in Supplemental Tables 2 and 3, which display the data for the comparisons *Kat6a*$^{-/-}$ *Kat6b*$^{+/+}$ vs. *Kat6a*$^{+/+}$*Kat6b*$^{+/+}$ embryos and *Kat6a*$^{-/-}$*Tg(Kat6b)* vs. *Kat6a*$^{+/+}$*Kat6b*$^{+/+}$ embryos.

To illustrate the major effects of loss of KAT6A n *Kat6a*$^{-/-}$ *Kat6b*$^{+/+}$ vs. *Kat6a*$^{+/+}$*Kat6b*$^{+/+}$ embryos. and restoration of expression in *Kat6a*$^{-/-}$*Tg(Kat6b)* vs. *Kat6a*$^{+/+}$*Kat6b*$^{+/+}$ embryos on the expression levels of the three important gene families that are relevant to the phenotypic anomalies observed in *Kat6a*$^{-/-}$ *Kat6b*$^{+/+}$ embryos, namely the HOX, DLX and TBX gene

families, we are now illustrating GSEA results in barcode plots for these gene families specifically in Supplementary Figure 3d and 3e.

In this context it is important to note that, while gene set enrichment analyses are an excellent tool for the discovery of disease processes, such as inflammation and immune response, they are less useful for understanding mechanisms of embryonic development. This is because disruption of embryonic development can occur when a single gene or a small subset of genes fail to be expressed. It does not have to be the majority of a gene set.

We can further illustrate this point using the data under consideration here. The GO term "GO:000738 pattern specification process" comprises 467 genes, of which 54 are downregulated in *Kat6a*$^{-/-}$ *Kat6b*$^{+/+}$ vs. *Kat6a*$^{+/+}$*Kat6b*$^{+/+}$ embryos. It is the GO term most significantly enriched in the genes downregulated in *Kat6a*$^{-/-}$ *Kat6b*$^{+/+}$ vs. *Kat6a*$^{+/+}$*Kat6b*$^{+/+}$ embryos (position 1; Supplementary Table 2, Tab 2), yet it only makes it to position 71 in the GSEA (position 71; Supplementary Table 2, Tab 3). Nevertheless, this GO term is of utmost importance, as it comprises the genes that underlie the three major phenotypic anomalies in *Kat6a*$^{-/-}$ *Kat6b*$^{+/+}$ embryonic development: HOX genes for the extensive anterior homeotic transformation affecting 19 body segments, DLX genes for the craniofacial and cleft palate and TBX1 for the aortic arch and cardiac septum defects. Of note, the latter are lethal phenotypes. Therefore, relegating this GO term position 71 instead of 1 would not help the discovery of the important changes in gene expression, if one did not already know of the importance of HOX, DLX and TBX genes. Furthermore, the GO term that is on position 1 in the GSEA, "GOBP positive regulation of acute inflammatory response to antigenic stimulus" is much less relevant to embryonic development, because the adaptive immune system only begins to develop at birth.

6. I may also be worth to have a closer look on the gene expression changes Kat6a+/+ Tg(Kat6b) vs. wildtype, to address the effect of KAT6B overexpression.

Response:
As the reviewer requested, we have now also examined the RNA-seq results of the *Kat6a*$^{+/+}$*Tg(Kat6b* vs. *Kat6a*$^{+/+}$*Kat6b*$^{+/+}$ embryos in more detail. The new data are shown in the new Supplemental Figure 4j-l). It is important to note that there are only 74 differentially expressed genes between *Kat6a*$^{+/+}$*Tg(Kat6b* vs. *Kat6a*$^{+/+}$*Kat6b*$^{+/+}$ embryos. The majority are down regulated, these are not consistently associated with a specific KEGG pathway, as can be seen in l only 3 KEGG pathways are enriched and this driven by less than 10% of the DE genes. It might seem surprising that overexpressing of KAT6B does not have a more substantial effect. However, it needs to be taken into consideration that KAT6B requires its protein complex partners (BRPF1, 2 or 3; ING4 or 5 and MEAF6) to function and that the genes encoding these proteins are not overexpressed. This limits the effects that overexpression of KAT6B in isolation can have. In contrast, as KAT6A and KAT6B share these protein complex partners, in the absence of KAT6A, more of these protein complex partners are likely to be available and so the effects of overexpressing KAT6B are likely to be more profound in the absence than in the presence of KAT6A.

7. In my view, the discussion falls short to explain how KAT6B is able to compensate for KAT6A, and should be improved.

Response:
To address the reviewers’ comment, we have added to the discussion without engaging in any speculation, which would result in an excessively long discussion section, to augment the pre-existing discussion paragraphs 1 and 2 page 17. The changes can be found on page 16.

Minor points:
1. Figure 1a: Why are some of the exons (grey, white) thinner than the others? In the event the authors want to show that these regions do not have domains: Please check out the Alphafold database. There is a domain at the very N-terminus and neither the acidic nor the serine and methionine-rich regions are domains in the classical sense.
I would discourage the usage of the term "NEMM", because it is outdated and not very informative. Recent work suggests that the NEMM consists of two separate winged-helix domains (PMID: 36754959, 36537216). Showing these two domains in Figure 1a would be more useful.

Response:
As the reviewer requested, we have changed Figure 1. We removed NEMM, included the winged-helix domains, and removed the term domain in association of the acidic, serine-rich and methionine-rich regions. The thinner, grey exons are non-protein-coding parts of the mRNA, which is now also indicated in the colour key within the figure.

The similarity between certain domains/regions are shown. What is the similarity of the acidic part? What is the similarity (and identity) globally?

Response:
The amino acid similarity in the acidic region is not high. However, the acidic property of this region is conserved.

2. "Overexpression of Kat6b in Kat6a–/– Tg(Kat6b) embryos reduced the number of differentially expressed genes compared to Kat6a+/+Kat6b+/+ embryos to 285 genes, 170 downregulated and 121 upregulated". 170 + 121 = 291, and not 285.

Response:
We thank the reviewer for noting this mistake. The numbers of genes changed have now been corrected in the figure.

3. The title of the supplementary data is different from that of the main article. (Personally, I find the title of the supplementary better)

Response:
We thank the reviewer for noting this mistake. We have now ensured that the title is the same in both documents.

4. In many figures the fonts are very small and hard to read. I recommend increasing the fonts sizes.

Response:
We agree with the reviewer and have increase the font sizes in Figures 2, 3 and 7 and in Supplemental Figure 3.

*****

Reviewer #3 (Remarks to the Author):

The manuscript by Bergamasco et al examines the role of closely related paralog function, Kat6a and Kat6b in hematopoietic stem cells and skeletal development. Specifically, the authors tested the idea that overexpression of one paralog, Kat6b, would be able to rescue the Kat6a developmental defects. Given the high similarity, the authors show that H3K23ac and H3K9ac are rescued to wildtype levels, as well as fewer numbers of gene expression changes between rescue and wildtype and normalized hematopoietic stem cells. In addition, all aspects of skeletal and palate development are rescued as well as viability. The paper describes a very interesting and novel phenomenon between closely related paralogs which is unique. It is well written and illustrated and is of interest to the readers of Nature Communications. There are a few suggestions that would strengthen the manuscript. My comments are below:

1.The overall acetylation and morphological rescue is interesting. Does Kat6b overexpression rescue Kat6b mutants?

Response:

Yes. We have shown that the transgenic expressing *Kat6b* rescues the hematopoietic defect in *Kat6b*$^{-/-}$ mice Bergamasco et al Stem Cell Reports 2024.

What is the overexpression phenotype of Kat6b without Kat6a in the background?
These are important control for functionality of Kat6b and that it is/ is not taking on a new function.

Response:

Briefly, overexpressing the *Kat6b* gene does result in a phenotype prominent in adult mice. Adult transgenic mice show aggression, anxiety, and spontaneous epilepsy. *Kat6b* overexpression caused an upregulation of genes driving neural stem cell proliferation and neurogenesis. *Kat6b* overexpression additionally promoted neural stem cell proliferation and favored neuronal over astrocyte differentiation in vivo and in vitro. In addition, transgenic mice have increased rates of cancer. In particular female transgenic mice have a propensity to develop uterine tumors.

This is the topic of a publication under review.

2. This brings up the interesting observation that in the Kat6a+/+; Tg(Kat6b) animls there is a significant increase in H3K9 acetylation but seeming no phenotype in the embryos. Is this true of is there some phenotype in the mice morphologically? Quantification of the size for the skeletal elements would be important here to determine this. What do the authors think is the explanation for not having an overexpression phenotype?

Response:

To address the reviewer's question regarding skeletal elements, we have generated a new supplemental figure which shows the dissected cervical vertebrae. These are of normal size and morphology in the *Tg(Kat6b)* and *Kat6b*$^{-/-}$ *Tg(Kat6b)* compared to wild type.

There is an overexpression phenotype primarily affecting the brain which is most obvious in adult animals. On an inbred C57BL/6 and other inbred backgrounds 100% of the *Kat6b*

transgenic heterozygous mice are die perinatally. No pups reach weaning if they are more than 75% C57BL/6. So, it is somewhat surprising that the gene expression at E9.5 in the transgenic embryos is so similar to the wild type animals (new Figure 2a-c). In the hematopoietic system (Bergamasco et al Stem Cell Reports 2024) the presence of the *Kat6b* transgene has a very minor effect of haematopoiesis, yet is able to completely rescue the loss of HSCs in *Kat6a* KO mice.

A possible explanation for the lack of an over phenotype during development is that "KAT6" proteins do not act in isolation but form a complex with other proteins, notably BRPF and ING proteins. So, in the absence of additional cofactors, it is likely that the level of KAT6B in a functional complex does not increase that much. Indeed, when we have overexpressed FLAG-tagged KAT6B in cell lines we have found that it is degraded in the absence of sufficient quantities of these accessory proteins.

Is there a threshold that needs to be reached to cause an overexpression phenotype?
The discussion section is a good start, but more information on this would be useful including references if available. It suggests that Kat6b can rescue when Kat6a is absent but is not able to add to the levels for overexpression. Similarly it would be interesting to know if this is true without Kat6a in the background (see point 1).

Response:
As noted in our answer to the previous question, we hypothesise that a key element in an overexpression phenotype will be the quantity of co-factor proteins available. Relevant to this question is that mice with a combination of loss of KAT6A and gain of KAT6B are viable, survive to adulthood and breed. In contrast, *Kat6a*$^{-/-}$ mice are prenatally to perinatally lethal on any background and *Tg(Kat6b)* heterozygous mice are perinatally lethal on the C57BL/6 background*, except,* as listed below, in combination of loss of KAT6A and gain of KAT6B:

Viable and breeding are:
Kat6a$^{-/-}$*Tg(Kat6b)/+; Kat6a* homozygous null, transgenic heterozygous; namely the work presented in this manuscript.
Kat6a$^{-/-}$, *Kat6b*$^{-/+}$*Tg(Kat6b)/+,* i.e*., Kat6a* homozygous null, *Kat6b* heterozygous, transgenic heterozygous.
Kat6a$^{-/-}$, *Kat6b*$^{-/+}$*Tg(Kat6b)/Tg(Kat6b);* i.e., *Kat6a* homozygous null, *Kat6b* heterozygous, transgenic homozygous.
Kat6a$^{-/-}$, *Kat6b*$^{-/-}$*Tg(Kat6b)/Tg(Kat6b);* i.e., *Kat6a* homozygous null, *Kat6b* homozygous null, transgenic homozygous.

So, it can be seen that not only does the transgene rescue the *Kat6a/Kat6b* phenotype but that loss of *Kat6a* and *Kat6b* rescues the transgenic phenotype of perinatal death.

3. For the gene expression, the RNAseq clearly shows that gene expression changes are more normalized and similar to wildtype Kat6a and Kat6b. However from the heat map, there are a significant number of genes that remain upregulated and downregulated following the cross with Tg(Kat6b). It would be interesting to show what these genes are. And while validation of the sequencing results are not necessarily required, it would be very interesting to show the expression by in situ of some of the genes that are normalized and the ones that not rescued, especially in relation to the homeotic transformation data (ie Hox genes). It would be important to prove that this is indeed the case in the embryo by looking at gene expression at earlier time points.

Response:
As the reviewer requested, we have examined the genes not rescued in the *Kat6a*$^{-/-}$ *Tg(Kat6b)* embryos compared to wild-type embryos and, in addition, differences in gene expression between the *Tg(Kat6b)* alone compared to wild type. The new data can be found in Supplemental Figure 3 and Supplemental Tables 3 and 4.

As the reviewer requested, we have performed in situ hybridization on representative *Hox* genes. The RNA-seq experiment shows that in *Kat6a*$^{-/-}$ embryos, the level of *Hox* gene expression is reduced as we have published previously, and that in the *Kat6a*$^{-/-}$ *Tg(Kat6b)* embryos the level of expression is restored. However, as the reviewer has implied, the RNA-seq experiment is not able to establish if the wild type anterior boundary of *Hox* gene expression is restored in the transgenic rescue embryos. We have previously shown (Voss 2009) that the anterior boundary of *Hox* gene expression is shifted in the posterior direction leading to an anterior homeotic transformation in *Kat6a*$^{-/-}$ embryos.

To address this point we performed in whole-mount situ hybridization on 3-5 embryos of each of the 4 relevant genotypes; *Kat6a*$^{+/+}$*Kat6b*$^{+/+}$, *Kat6a*$^{-/-}$ *Kat6b*$^{+/+}$, *Kat6a*$^{-/-}$*Tg(Kat6b)* and *Kat6a*$^{+/+}$*Tg(Kat6b),* repeated twice, probing for *Hoxa5, Hoxc5* and *Hoxa3*. This showed that the normal anterior boundary is restored in the *Kat6a*$^{-/-}$*Tg(Kat6b)* animals along with the level of expression and, as a result, the segmental patterning of the embryo is restored to normal. The new data are displayed in main Figure 4 and Supplemental Figure 7.

Note: key to the function of *Hox* genes in patterning the axial skeleton is the position of the anterior boundary of the *Hox* genes expression. The posterior HOX proteins are dominant over the more anterior expressed HOX proteins, ie the specific effect of a given *Hox* gene is “prevalent” in the anterior part of its expression domain where fewer competing (posterior) *Hox* genes are expressed. If this expression domain is shifted posteriorly then the segment acquires an anterior phenotype.

4. The authors present that there elevated Cd8 and reduced Cd4 T cell levels in Kat6a+/+; Tg(Kat6b) animals. What is the significance of this? And why does it happen specifically in this population? Please expand the discussion around this.

Response:
We have previous published a paper KAT6A role in CD8 T cells (*Acetylation of the Cd8 Locus by KAT6A Determines Memory T Cell Diversity;* Newman DM et al Cell Reports, 2016) in which we show that KAT6A is required for the activity of an upstream enhancer regulating the expression of CD8. Since single positive T cells (either CD4^{+} or CD8^{+} positive) are derived from CD4^{+}CD8^{+} double positive progenitors, we can speculate, based on our previous publication Newman et al and on the data presented in this manuscript, that the level of *Kat6a/Kat6b* could shift the balance during differentiation. In this context it is interesting to note that the *Kat6b* transgene leads to a highly significant upregulation of CD8^{+} T cells (old Figure 4g,h new Figure 3g,h). This is potentially an interesting topic for a further project since the balance between CD4^{+} regulatory T cells and CD8^{+} killer T cells is very much an active area of research because of the role of CD8^{+} T cells in protecting against cancer.

Minor: 1. Would be very helpful to keep the same colors/position for each genetic condition in every graph.

Response:
We thank the reviewer for this request. We have changed the colours to associate blue only with the *Kat6a*$^{-/-}$*Kat6b*$^{+/+}$ samples and purple only with the *Kat6a*$^{-/-}$*Tg(Kat6b)* samples, with the addition of further colours. We hope this addresses this request.

REVIEWERS' COMMENTS

Reviewer #2 (Remarks to the Author):

The authors did an overall great job to improve the manuscript. They convincingly addressed all reviewers questions. I have only one minor point: please make the Cut&Tag data publicly available.

Response: Thank you for the positive comments. The Cut&Tag and RNAseq data has now been uploaded to the NCBI GEO database.

Reviewer #3 (Remarks to the Author):

The authors have carefully addressed the concerns of the previous review. The topic of genetic redundancy is of importance to the field, and they begin to tease out the mechanism of how this occurs. The addition of the new data strengthens the paper significantly and I appreciate the answers to my questions. A couple minor suggestions/clarification is needed on the new data.
The in situ hybridization is a nice addition to the figure. It would be important to include some higher magnification insets especially for the hoxc5 since it is difficult to see the difference here.

Response: Thank you for the positive comments. A higher magnification of the Hoxc5 WMISH has been added to the supplementary data.

Similarly, the Cut and Run data nicely shows changes in specific loci (Figure 6e-g). Interestingly the levels of the peaks are increased in the Kat6a+/+ Tg(kat6b)+/+, getting back to the question of why there is no phenotype in the overexpression. It would be great to add text to the discussion section around this issue of dose and/or lack of cofactors etc, as it will be important to determine to refine the mechanism.

Response. The level of the peaks are not significantly increased in the Kat6a+/+ Tg(kat6b)+/+ across the genome MEFs. Please see supplemental figure 8. Please note also, that is not the case that the Tg(kat6b)+/+ mice have no phenotype at all. As stated in the methods section under mouse alleles in the previous version and now in the second paragraph of the results section, the transgene is lethal on all inbred mouse strains tested and they only survive to adulthood on an FVBxBALBc hybrid strain. While we consider that it is a very interesting that we do not see a phenotype in the transgenics in the systems acutely regulated by KAT6A, we feel that a discussion of this phenomena would be rather speculative and so better suited to a review article.
